# Implementation of an Open-Source Multiplexing Ion Gate Control for High Kinetic Energy Ion Mobility Spectrometry (HiKE-IMS)

**DOI:** 10.1021/jasms.3c00013

**Published:** 2023-06-05

**Authors:** Cameron N. Naylor, Brian H. Clowers, Florian Schlottmann, Nic Solle, Stefan Zimmermann

**Affiliations:** † Institute of Electrical Engineering and Measurement Technology, Department of Sensors and Measurement Technology, Leibniz University Hannover, 30167 Hannover Germany; ‡ Department of Chemistry, Washington State University, Pullman, Washington 99164, United States

**Keywords:** ion mobility spectrometry, multiplexing, Hadamard transform, open-source

## Abstract

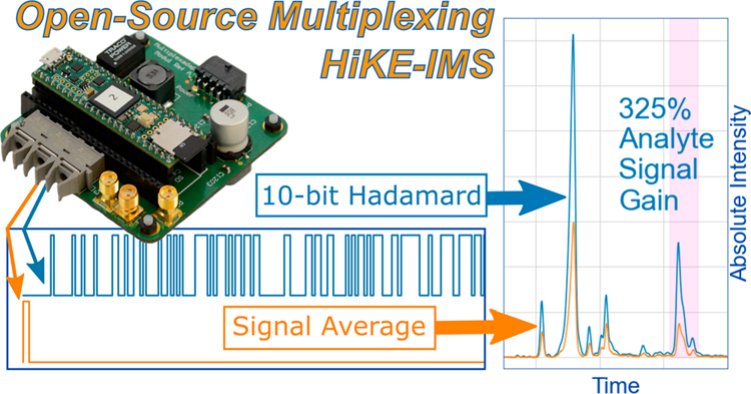

With ion mobility spectrometry increasingly used in mass spectrometry to enhance separation by increasing orthogonality, low ion throughput is a challenge for the drift-tube ion mobility experiment. The High Kinetic Energy Ion Mobility Spectrometer (HiKE-IMS) is no exception and routinely uses duty cycles of less than 0.1%. Multiplexing techniques such as Fourier transform and Hadamard transform represent two of the most common approaches used in the literature to improve ion throughput for the IMS experiment; these techniques promise increased duty cycles of up to 50% and an increased signal-to-noise ratio (SNR). With no instrument modifications required, we present the implementation of Hadamard Transform on the HiKE-IMS using a low cost, high-speed (600 MHz), open source microcontroller, a Teensy 4.1. Compared to signal average mode, 7- to 10-bit pseudorandom binary sequences resulted in increased analyte signal by over a factor of 3. However, the maximum SNR gain of 10 did not approach the theoretical  gain largely due to capacitive coupling of the ion gate modulation with the Faraday plate used as a detector. Even when utilizing an inverse Hadamard technique, capacitive coupling was not completely eliminated. Regardless, the benefits of multiplexing IMS coupled to mass spectrometers are well documented throughout literature, and this first effort serves as a proof of concept for multiplexing HiKE-IMS. Finally, the highly flexible Teensy used in this effort can be used to multiplex other devices or can be used for Fourier transform instead of Hadamard transform.

## Introduction

As ion mobility spectrometry (IMS) becomes more commonly coupled prior to other separation techniques, namely mass spectrometry, and used for applications such as trace detection, challenges are apparent with regards to efficient ion transmission. Specifically, in signal average (SA) drift tube ion mobility spectrometry (DT-IMS), the ion gate pulses once for a span of a few microseconds before the gate closes again.^1^ When comparing the time the ion gate opens (tens of microseconds) to the duration of the ion mobility experiment (tens of milliseconds), the resulting duty cycle of the SA-IMS experiment is less than 0.1%.^2−4^ This low duty cycle causes significant analyte loss in cases of trace detection where the amount of sample may be limited, or when coupling the IMS to a mass spectrometer, where the low duty cycle results in lower ion transmission. The majority of IMS-MS experiments utilize time-of-flight (TOF) mass spectrometers because the time scales of separation are compatible.^5^ However, coupling an IMS to some types of mass spectrometers (MS) (i.e., Orbitrap, linear ion traps, FTICR) also introduces the additional challenge of a time scale mismatch since these mass analyzers are slower than the traditional IMS experiment.^6−9^ Since these mass analyzers are slower than the IMS, an arrival time distribution from the IMS cannot be constructed by traditional signal averaging and nesting of the mass spectrum in the IMS time domain. In fact, if a strict focus on the time domain and signal averaging is maintained the entire arrival time distribution is only accessible by sequential summation of thin slices of the drift time space using a second ion gate.^10^ As a result, the IMS-MS experiment becomes significantly lengthened, taking hours to obtain full two-dimensional spectra.^10^

One solution to both these issues, low ion throughput and the mismatch in experiment time scale, lies in increasing the duty cycle of the ion gate through multiplexing. The two most commonly implemented multiplexing techniques in IMS are the Fourier transform (FT) and the Hadamard transform (HT), a specialized subset of the Fourier transform.^2^ Both techniques use an array of ones and zeros that correspond to the state of the ion gate of opened and closed, respectively, to modulate its pulsing sequence, but the practical implementation of both of these techniques differ slightly from each other. Fourier transform is most commonly used on IMS-MS instruments with two ion gates since two gates are pulsed in tandem at increasing frequency over time called the sweep rate.^6,11,12^ The sweep rate of the FT experiment must be carefully defined to ensure a Gaussian peak shape (i.e., longer experiments ensure at the bare minimum that the Nyquist frequency is preserved) and can be further fine-tuned to increase resolution and accuracy of ion mobilities.^13^ One major problem is the interpolation of the exact frequency the gates are operating at to the ion signal, although recent efforts show changing the sweep function can mitigate this issue.^13−15^ The other multiplexed technique is Hadamard transform as first shown by Clowers et al.^16^ and shortly followed by Szumlas and Hieftje.^17,18^ The HT experiment only requires one ion gate and uses a set of pseudorandom binary sequences (PRBS) to pulse the ion gate promising a duty cycle approaching 50%.^2^ The PRBS can be generated either through a series of algorithms, such as those from Harwit and Sloane, Barker codes, almost-perfect sequences, or a random number generator.^19−24^

In addition to increased duty cycle, another benefit comes with HT-IMS: increasing signal-to-noise ratio. For HT-IMS, the theoretical SNR gain is a function of the number of bits in the sequence, and in the literature, SNR improvement has been reported to be improved over a factor of 12 compared to signal average mode.^25−27^ Because of the theoretical SNR gains and the ability to couple IMS to higher resolution mass analyzers, multiplexing is an attractive idea, but practical implementation can be a challenge. For example, instrumental design between different IMS platforms can vary significantly, where the electronics that operate the ion gating mechanism may not be physically accessible. Some commercial platforms, such as the Excellims 3100 and Agilent 6560, are designed to be a closed environment that take no external gate trigger and are limited by software.^6,28^ Whereas for in-lab-built instruments, often the gate controller is more accessible, but not all laboratories that use IMS may opt to build their own instrument.^14^ Additionally, traditional multiplexing requires expensive waveform generators and software to operate the waveform generator to send sequences to the ion gate and synchronize the measurements.^6,7,15,29^ Both accessibility to modify instruments and the cost of waveform generators complicate implementing multiplexing for a broad range of users.

With the promise of the potential SNR gain and improved ion throughput, here we present an open-source Teensy 4.1 microcontroller to control the ion gate driver to implement Hadamard multiplexing on the High Kinetic Energy IMS (HiKE-IMS) with no additional instrument modifications. The Teensy microcontroller is inexpensive and capable of fast, accurate pulses well-suited not just for HiKE-IMS, but for any IMS platform. The HiKE-IMS is already capable of high SNR due to the instrumental design including the electronics, high ion current, and thousands of averages over a few seconds obtained for each spectra.^3,30−33^ Furthermore, the fundamental operating parameters of the HiKE-IMS present interesting challenges and opportunities for implementing multiplexing with regards to the ion gating event. The performance of the Teensy coupled to the HiKE-IMS is evaluated by comparing Hadamard spectra with signal-averaged spectra for a number of small, volatile, flavor compounds. These compounds originate from a number of natural sources, including wood and various food stuffs, and may require an additional separation technique to IMS to identify in those complex matrices.^34−37^ Various sequences and Hadamard parameters are examined and establish the groundwork, including discussion of assumptions and potential pitfalls, for implementing multiplexing on IMS platforms.

## Materials and Methods

### Chemicals

The following small, volatile flavor compounds were purchased from Sigma-Aldrich (Taufkirchen, Germany) and inserted into the permeation oven (Dynacalibrator Model 150, Vici Metronics Inc.) at 30 °C without further purification: α-pinene (Sigma: 147524), limonene (Sigma: 183164), linalool (Sigma: L2602), cinnamaldehyde (Sigma: W228613), and ethyl butyrate (Sigma: E15701). The Bronkhorst flow controllers (IQF-200C-ABD-00-V-S; F-201DV-RBD-33-V; FS-201CV-500-RBD-33-V; F-201DV-2k0-RBD-33-V; P-502C-100R-RBD-93V) used to control drift and analyte gas flow are calibrated to mass flow at reference conditions (20 °C and 1013.25 mbar) for milliliter standard per minute.

### Instrumentation

The HiKE-IMS has been described thoroughly elsewhere, but briefly, the HiKE-IMS is a drift tube IMS with a few key differences from other DT-IMS instruments.^3,32,33^ Briefly, the HiKE-IMS contains a reaction region immediately before the tristate ion gate, which allows for the formation of reactant ions via corona discharge with the background gas and ionization of analyte molecules via the formed reactant ions.^38,39^ For the HiKE-IMS used in this effort, the drift region is 101.5 mm in length ending with a Faraday plate detector.^40^ The HiKE-IMS is operated at reduced pressures between 7 and 60 mbar which allows for operation at high reduced electric field strengths (*E*/*N*) up to 120 Td.^3,32^ For these experiments, this HiKE-IMS is operated at the conditions stated in Table 1 unless specified otherwise. More detailed information about the construction of this HiKE-IMS is given by Schlottmann et al.^40^

**Table 1:** Experimental Variables Used to Acquire the Data for Each Figure^a^

experimental variable	value
temperature	40 °C
pressure	40 mbar
*E*_DR_/*N*^a^	80 Td
*E*_RR_/*N*^a^	20 Td
gate pulse width (GPW) (bin size)	1 μs
drift region length	101.5 mm
reaction region length	34.8 mm
corona voltage	1450 V
drift gas (air) flow rate	10 mL_s_/min
analyte gas (air) flow rate	10 mL_s_/min
amplifier bandwidth	248 kHz
amplifier gain	45 MΩ

a Unless indicated otherwise.

**Figure 1:**
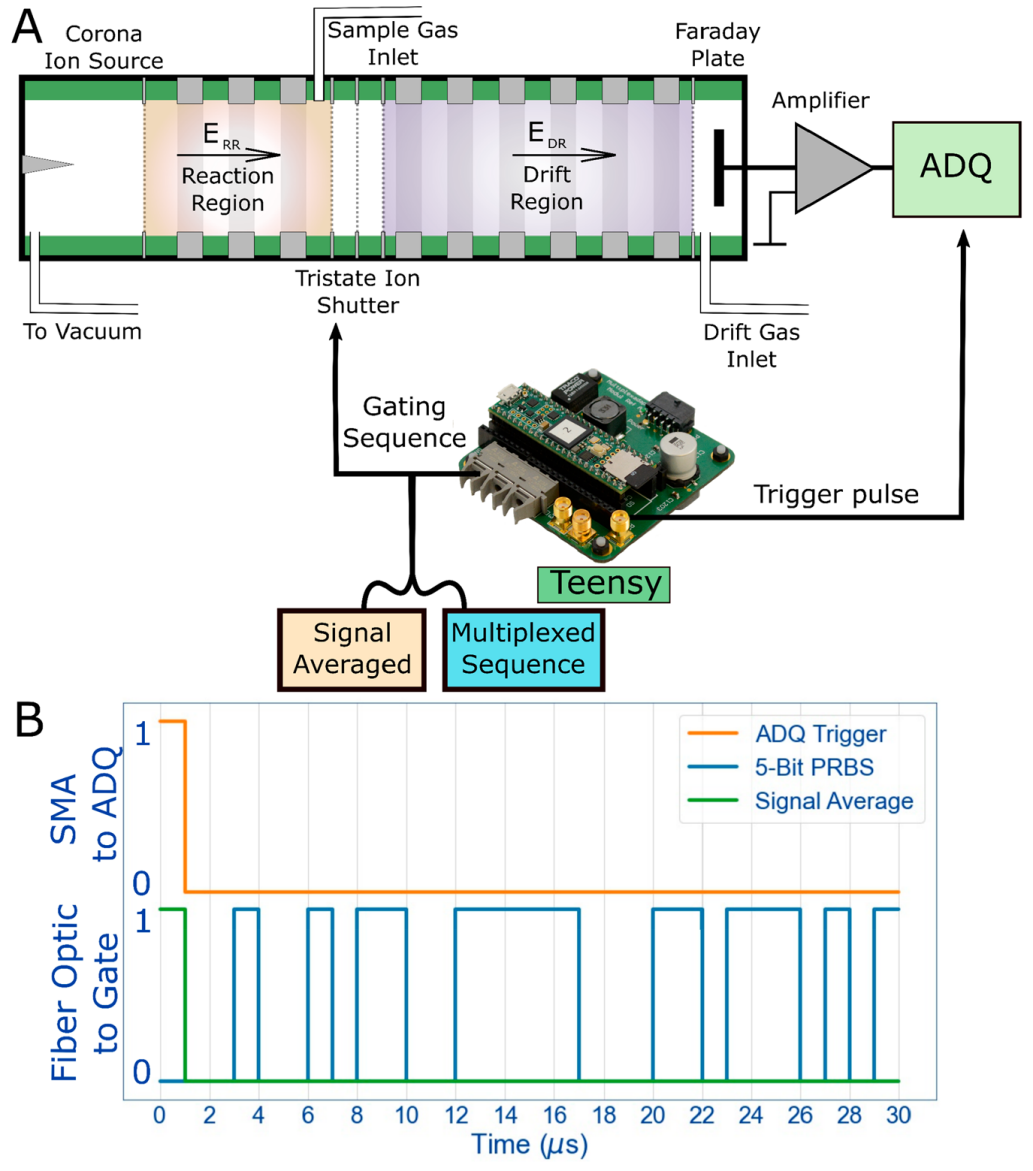
Instrumental diagram. (A) A simple diagram of the HiKE-IMS with the addition of the Teensy interfaced to the IGC (ion gate controller) and analog/digital converter. The signal sent from the Teensy to the IGC can be either signal average mode or a multiplexed sequence. (B) The waveforms sent from the Teensy to both the IGC via fiber optic and the ADQ via SMA are illustrated as a series of pulses.

To implement multiplexed ion gating on the HiKE-IMS, a Teensy 4.1 (https://www.pjrc.com/store/teensy41.html) was purchased and mounted to an in-house designed PCB board (Figures 1 and S4). The PCB board mount (Table S1, Figure S4), requires 24 V to operate at approximately 21 mA (0.5 W). The chosen sequence from the SD-card is transmitted via fiber optic transmitters (Avago Technologies, AFBR-1624Z) to either one or two ion gates. The mounting board also has SMA connectors to either receive an input signal and trigger the sequence output event, or output a pulse to trigger the data acquisition. The only modifications to the Teensy itself was the addition of 16 MB of RAM, the insertion of an SD-card containing the PRBSs in a csv format, and a micro-USB cable to connect the Teensy with a computer. The sequences were selected by using either Arduino software (Version 1.8.19, Teensyduino 1.56) or PuTTY (version 0.78.) to communicate via COM port to the Teensy. Once selected, the sequence is loaded from the SD card into RAM where, upon instrument trigger or user input, the sequence is sent to the fiber optics to trigger the ion gate controller (IGC) to pulse. Simultaneously, a single starting pulse is sent to the SMA to trigger data collection on the analog digital converter data acquisition device (ADQ, Teledyne SP Devices ADQ14DC-2A-USB) (Figure 1). All code for generating the PRBS, data analysis, and the Teensy were custom generated in Python and Arduino and freely available in the following GitHub repository (github.com/bhclowers/DAMS). Step-by-step pictures for Teensy operation are in the Supporting Information.

It should also be noted that due to the nature of using a microcontroller that is reading from RAM, there is a minimal offset in outputting the trigger and gate pulses which is negligible compared to the ion gate controller electronics receiving the trigger. Precisely, the timing of the pulse (length of 1 μs) was accurate with a jitter of less than 200 ps and a failure rate of 0.0125% for 8000 spectra measured on a Keysight Infiniivision 4000 X-Series (DSOX4104A, 1 GHz, 5GSa/s) oscilloscope. In our application and for most IMS instruments, any possible offset in the time scale of hundreds of picoseconds is insignificant to the time scale of the experiment.

### Theory

Like in any Hadamard IMS experiment, the length of the Hadamard sequence must be on a sufficient time scale for the ion mobility separation to occur. The length of the Hadamard sequence is defined by the number of bits (*n*) as 2^*n*^ – 1. Each unit of the sequence is called a bin and corresponds to how long each gate pulse width (GPW) occurs, resulting in the measurement time (*t*_meas_) as a function of gate pulse width and number of bits in the sequence below:
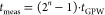
1

As a simple example calculation, on standard ambient pressure DT-IMS instruments, each bin (gate pulse) can last anywhere between 50 to 500 μs, depending on the instrument and experiment. For a 5-bit sequence, this means the measurement time for one spectrum lasts between 1.55 to 15.5 ms for each aforementioned gate pulse width respectively. Although 1.55 ms is too short of a measurement time for most ion mobility measurements for ambient pressure IMS instruments, 15.5 ms is more reasonable depending on analyte. However, such a large gate pulse width, like 500 μs, introduces other issues such as non-Gaussian peaks.^4,41−43^

**Figure 2:**
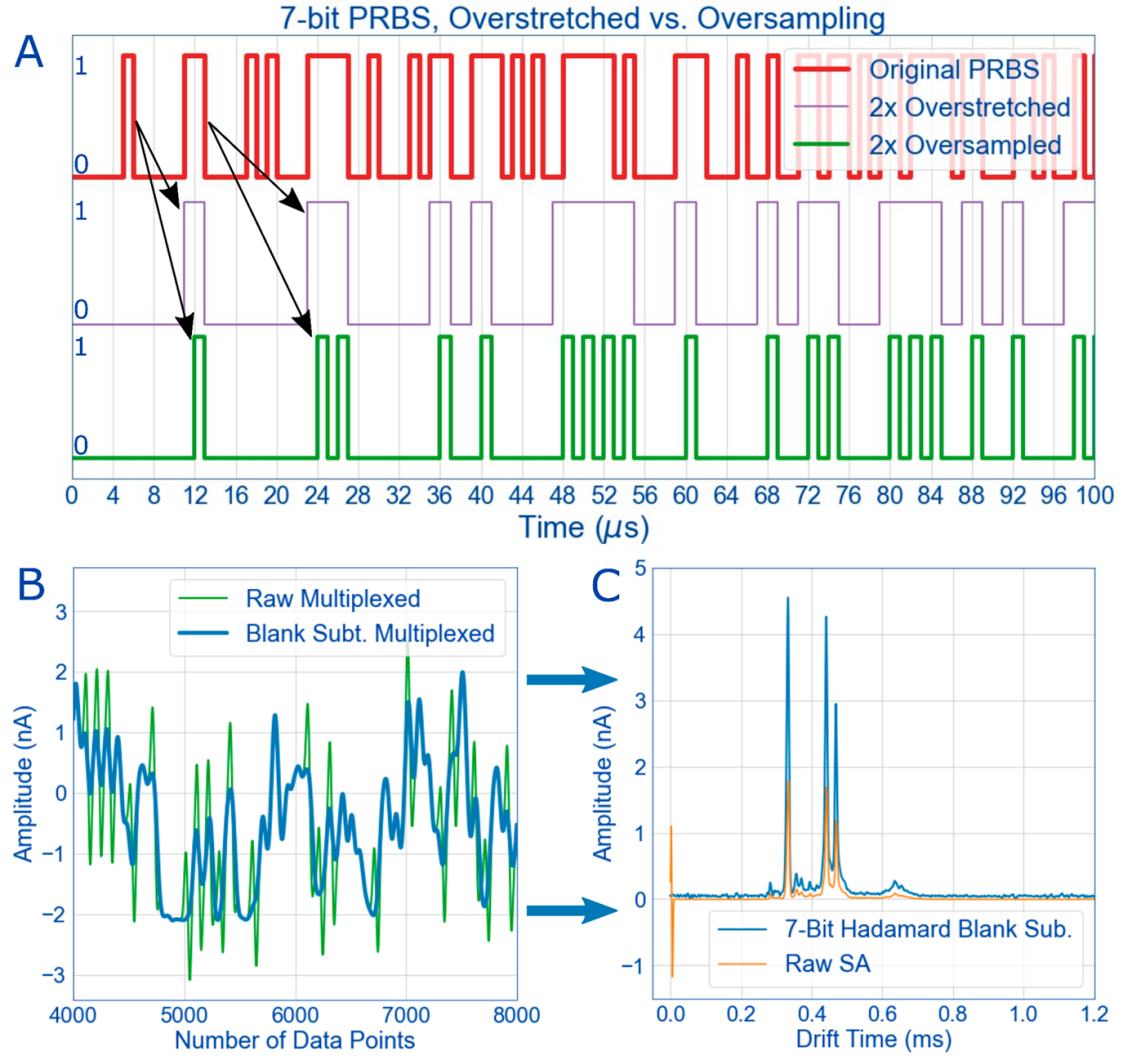
(A) To accommodate the Hadamard sequences to the required length of an ion mobility experiment, two techniques have been introduced in the literature: overstretching and oversampling.^19,20,25^ Overstretching (purple) simply multiplies each bin in the PRBS by the chosen factor (here, 2). Oversampling (green), instead, inserts additional spaces in front of each bin to extend the sequence by the chosen factor. The resulting matrix from these modifications can be accommodated in deconvolution. (B) Because the HiKE-IMS has a Faraday plate, the excess noise must be subtracted before deconvolution. (C) Once the excess noise from the Faraday plate is gone, deconvoluted Hadamard data gives a significant increase in signal compared with signal-averaged spectra (SA) of limonene recorded using the same measurement time.

However, because the HiKE-IMS is operated with the tristate ion gating mechanism, care must be taken in defining the GPW for Hadamard to work properly. First, the tristate, when implemented on HiKE-IMS, is restricted to 1–3 μs gate pulse widths, because larger pulse widths cause additional problems. One example is the ion packet experiences two different electric field strengths with longer pulse times by the different pulsing phases and the peaks becoming non-Gaussian. The fixed range of gate pulse widths between 1 and 3 μs means for the aforementioned 5-bit sequence, the mobility experiment would last 31–93 μs respectively. This experiment time frame is too short for ions with an expected drift time between 400 and 600 μs or reduced mobilities between 1.8 cm^2^ V^–1^ s^–1^ and 1.4 cm^2^ V^–1^ s^–1^ respectively.^3^ However, two strategies (other than increasing the number of bits in the sequence) are present in the literature which extends the length of the PRBS to accommodate an IMS experiment: overstretching and oversampling.^19,20,25^ Overstretching is when each bin is multiplied by a user-defined factor to extend the length of the sequence and has the same effect as increasing the gate pulse width (Figure 2A, purple trace) whereas oversampling inserts additional 0’s into the sequence to pad the length by some factor (Figure 2A, green trace). To be abundantly clear, although oversampling is a common term applied to analog digital converters, oversampling here means something completely different and is consistent with existing multiplexing literature.^19,25^ Although overstretching preserves the duty cycle of the original PRBS (approaching 50%), operating parameters of the ion gate controller are incompatible with the increased gate pulse width size of overstretching for two reasons. Overstretching results in an effectively larger gate pulse width, which causes decreased resolving power and possible oversaturation of the detector (i.e., peaks with the tops cut off). Additionally, the tristate mechanism requires a recovery time of a few microseconds that allows ions to fill the trap within the gate.^3^ For these reasons, oversampling was chosen as the method of sequence length extension to allow the tristate gating mechanism to work properly, preserving the high resolving power and at the cost of duty cycle (reducing the duty cycle from ∼50% for the original PRBS and overstretched to either 10% or 5% oversampled in the following experiments).

Additional considerations for HiKE-IMS multiplexing is the Faraday plate detector. With Faraday plate detectors, each time the ion gate pulses, an effect of capacitive coupling of the pulse is recorded on the Faraday plate (Figure 2C, orange trace at time 0), as there is a parasitic capacitance between the ion gate and the detector. This usually is not a problem for signal-averaged experiments as it appears within the first microsecond of the ion mobility spectra before any ion packet arrives at the detector. However, multiplexing involves multiple gate pulses during the course of the experiment while multiple ion packets move through the drift tube, the gate pulse significantly interferes with the ion signal (Figure 2B, Figure S1). A recent effort has addressed a way to mitigate this effect by changing the ion source to an orthogonal pulsed X-ray source and eliminating the ion gate altogether to reduce the effect of the capacitive coupling between the ion gate and Faraday plate.^44^ However, for our purposes, we cannot change the ion source or eliminate the ion gate, a blank spectrum (i.e., without ions) must be taken in addition to the Hadamard spectra and subtracted from the Hadamard spectra with ions present (Figure 2B). This treatment of the data partially mitigates the noise, but the noise from the ion gate will be addressed in more detail later.

Deconvolution of the Hadamard spectra is achieved with matrix algebra or a simple cross correlation. The overstretched or oversampled Hadamard sequence must match the number of data points collected in the spectra to achieve deconvolution. This exact number of data points can be achieved with interpolation in postprocessing to achieve the correct number of points, but for each experiment here, we specified the number of points (*p*) collected by our Teledyne ADQ to be a factor evenly divisible by the sequence length (2^*n*^ – 1). For example, in the 7-bit sequence oversampled 10 times (length = 1270), 10 data points were collected for each bin, resulting in a spectrum of 12700 points over 1.27 ms. The extended PRBS was then used to generate a matrix, the *A* matrix (*p* × *p*; in this case a 12700 × 12700 matrix) in eq 2 below, where *m* is the encoded ion signal at the detector and *x* is the ion mobility spectrum resulting from one gate pulse (bin):
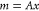
2

The inverse of the *A* matrix is then taken, and the first row of the matrix is used in circular deconvolution of the encoded ion signal, *m*, to obtain the demultiplexed spectra, *x*, in eq 3:
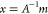
3

An interactive example of the data analysis and deconvolution is freely available at github.com/bhclowers/DAMS. Additionally, readers are encouraged to examine the work from Zare et al. for an in-depth walk-through of the theory and data deconvolution of Hadamard TOF-MS because the same principles apply here to Hadamard IMS.^26^ With a wide variety of experimental parameters to change in HiKE-IMS and multiplexing parameters, a number of different combinations can be explored to fully characterize the effect of multiplexing for HiKE-IMS.

## Results and Discussion

### Implementation of HT-IMS

**Figure 3:**
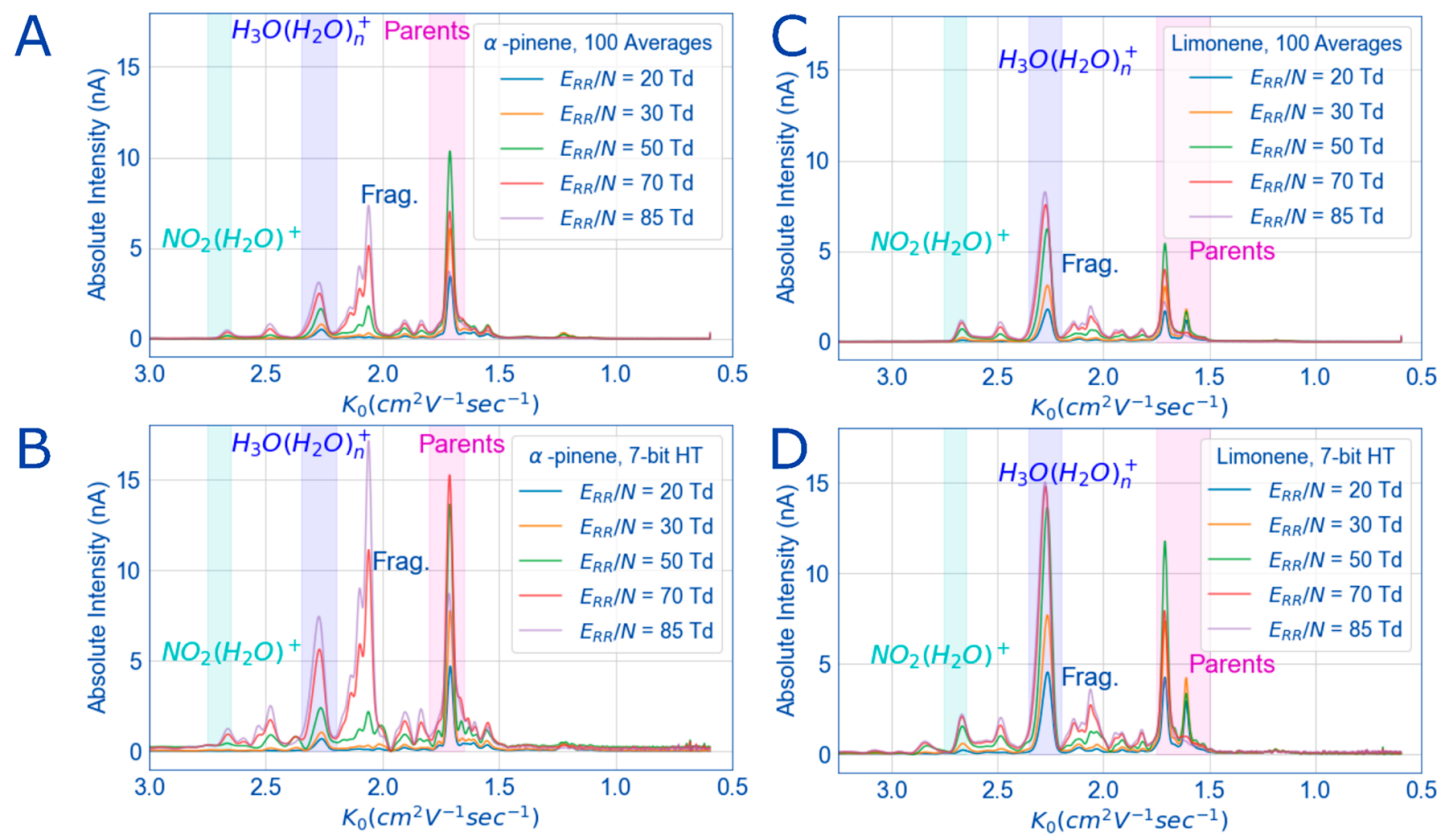
For both α-pinene (3.4 ppm_v_) and limonene (5.5 ppm_v_), when the *E*_DR_/*N* (50 Td) is kept the same and *E*_RR_/*N* is scanned from 20 to 85 Td, differences in signal between signal averaged spectra (A and C) and 7-bit multiplexed Hadamard (B and D) can be compared. For both compounds, multiplexing increases the signal of all peaks: reactant ions (cyan, blue boxes), parent peaks (magenta), and fragment peaks (all others). All relevant experimental details (except *E*_DR_/*N* and *E*_RR_/*N*, which are in the figure legend) are in Table 1.

By changing ionization conditions by varying the reduced electric field strength in the reaction region (*E*_RR_/*N*), the traditional HiKE-IMS experiment can be compared between signal average and multiplexed in terms of peak intensities. To contextualize the comparison, spectra were collected for the same number of signal averages (100) both in multiplexed mode and signal average mode. In Figure 3 for α-pinene and limonene, the absolute signal of all peaks more than doubles when the 7-bit, oversampled by 10 times, PRBS is used and compared with the signal averaged spectra. The characteristics of *E*_RR_/*N* scans in HiKE-IMS are present for both compounds in both gating modes. For example, many ions grow in intensity with *E*_RR_/*N* either from increased signal or fragmentation. For the water cluster and coeluting O_2_^+^ reactant ions (dark blue box, *K*_0_ = 2.3 cm^2^ V^–1^ s^–1^), this means growing in intensity with a noncentered peak, which has been shown before by Langejuergen et al.^30^ Parent analyte ions (magenta box, *K*_0_ = 1.5–1.8 cm^2^ V^–1^ s^–1^) also grow in intensity, before reaching a maximum, then decreasing due to fragmentation (*K*_0_ = 1.7–2.2 cm^2^ V^–1^ s^–1^). Because of increased ion signal, the multiplexing spectra also shows additional peaks that are not present in the signal-averaged spectra. This includes additional peaks are visible at *K*_0_ = 2.8 cm^2^ V^–1^ s^–1^ for limonene and *K*_0_ = 2.5 cm^2^ V^–1^ s^–1^ for α-pinene. These peaks are too broad and well-defined to be an artifact (see discussion below), and possibly might only be visible from the increased signal by multiplexing. The prominent peaks for both limonene and pinene are consistent with what have been shown by Vautz et al. although their IMS is a low field instrument coupled to a GC, and they do not structurally identify peaks other than naming them numerically as they appear in the spectra.^35^ Identification of the fragment ions would require additional analysis with mass spectrometry following HiKE-IMS separation, which may be interesting for a future effort but is outside the scope of this publication. Finally, for both signal average mode and 7-bit Hadamard the mobilities of all peaks are the same in both modes and thus comparable. While this first effort shows multiplexing is capable of increasing ion throughput compared to signal average and preserves the features in the HiKE-IMS reduced electric field strength sweeps, what multiplexing parameters can be changed to further increase throughput?

**Figure 4:**
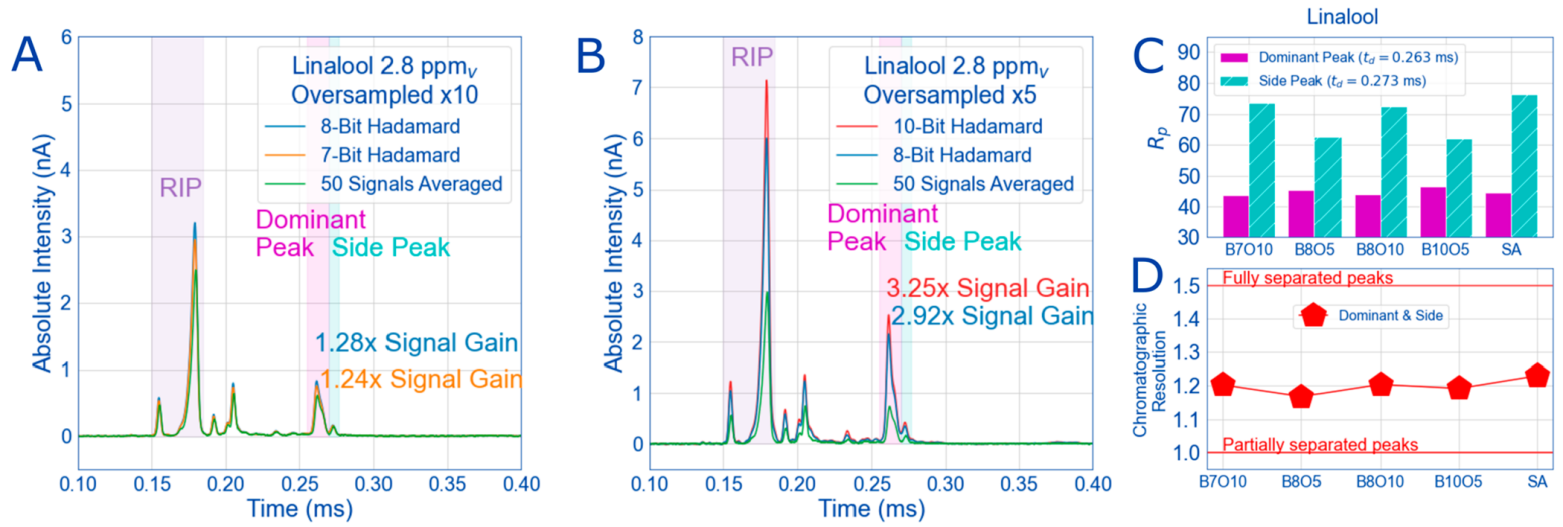
With 2.8 ppm_v_ linalool as the analyte gas and reactant ions present (RIP), the following oversampled Hadamard spectra are compared to an averaged 50 times spectra (green) (A and B). By reducing the number of times the sequence is oversampled by a factor of 2 (B), the increase in signal for the linalool peaks is over a factor of 2 when comparing the 8-bit Hadamard sequences (blue trace) between A and B. Even oversampling 10 times (A) at lower bits gives a small signal gain over signal averaged spectra (green traces). However, the gains in signal means increased fwhm for analyte peaks (pink and cyan boxes), reducing resolving power for all multiplexed spectra (C) and decreasing chromatographic resolution (separation) between peaks compared to signal average spectra (D). All relevant experimental details are in Table 1 and sequence abbreviations are in Table 2.

For multiplexing parameters, both the number of bits and the factor of oversampling greatly impact the increase in signal compared to traditional signal average. Increasing the number of bits, increases the number of ion gating events (i.e., more ions in the drift tube, more ion throughput), and decreasing the oversampling will also increase the duty cycle.^2^ In Figure 4A and 4B, linalool spectra are separated into two graphs based on the number of bits and the number of times the PRBS was oversampled (Table 2). When oversampled 10 times (Figure 4A), the further increase in signal compared to signal average is only 4% by increasing the number of bits in sequence from 7 to 8. When decreasing the oversampling factor from 10 to 5, for the 8-bit sequence the resulting increase in signal compared to SA is just over 200%, unsurprisingly from doubling the duty cycle. Unfortunately, due to the limitations of the IGC designed specifically for signal averaging IMS, oversampling by a factor of 5 is the lowest possible at this time. The IGC was left as-is to preserve the spirit of operating the Teensy without additional system modifications.

**Table 2:** Abbreviations for the Sequences Presented in Figures 4–6^a^

sequence abbreviation	number of bits	times oversampled	length of sequence (bins)	1 spectrum acquisition time (ms)
B7O10	7	10	1270	1.27
B8O10	8	10	2550	2.55
B8O5	8	5	1275	1.275
B10O5	10	5	5115	5.115

a The abbreviations also serve as file names of the sequences used in the Supporting Information.

When combining this oversample factor with the highest bit sequence, the maximum gain compared to signal average is by a factor of 3.25 for linalool. The theoretical gains in SNR compared to signal average from these settings should correspond to , as outlined by Zare et al.^26^ However, these theoretical gains are for a nonoverstretched sequence with a duty cycle approaching 50% and the actual gains will be significantly less due to decreased duty cycle. The gain in experimental SNR for this sequence (10-bit, 5× oversample) is 2.7, which is less than 1/10th of the gain predicted by theory. However, when looking at the 8-bit sequence, the experimental SNR gain is significantly better for both oversampling experiments (factor of 3.7 for 10× oversample, factor of 3.6 for 5× oversample). While it is promising that SNR is slightly improved from multiplexing, there are clearly other factors involved in the 10-bit experiment causing a reduction of SNR gain compared to the 8-bit sequences, which prompts a more thorough investigation.

It should also be emphasized here that Hadamard multiplexing only increases the signal of ions in the drift tube by increasing the number of ion packets in the drift tube at one time. Separation of those ion packets in the drift tube will be determined by the same conditions that affect separation in a normal signal averaged mobility experiment (i.e., electric field strength, diffusion, etc.).^4,41^ This means multiplexing does not cause better separation between the peaks and does not give better resolution compared to signal average without additional data treatment as some recent literature claims.^28,45,46^ This is evident in Figure 4C and 4D where the highlighted peaks of linalool in Figure 4A and B are plotted as their respective resolving power (Figure 4C) and the chromatographic resolution (Figure 4D) between these peaks as a function of the multiplexed sequence used. In every case, the signal-averaged spectra have no significant difference in resolving power than the oversampled 10 times multiplexed spectra in terms of resolving power and chromatographic resolution. In cases where oversampling is the lowest (5 times) and duty cycle the highest (10% in Figure 4B), the chromatographic resolution and resolving power are the lowest out of all tried sequences.

Notably, these resolving powers for the dominant peak of linalool are uncharacteristically low for the HiKE-IMS. When comparing the resolving powers (both multiplexed and signal averaged) for other multiple peaks of other compounds (cinnamaldehyde and ethyl butyrate, spectra in Figure S10), the majority average between 60 and 80. For reference, the highest resolving power so far being *R*_*p*_ = 140.^3^ The reason the dominant peak of linalool at 0.263 ms has a lower resolving power is there is a distinctive shoulder that indicates there is an additional linalool peak underneath artificially widening the Gaussian peak used during peak fitting. Therefore, there are three total linalool peaks under the highlighted areas in Figure 4, not just two, and they are not better separated by multiplexing. A similar phenomenon happens for peak 1 of ethyl butyrate with a resolving power of 40, but this peak is much smaller in intensity which further lowers the resolving power due to the shoulder peak. As shown with Figure 4 and Table 3, multiplexing by itself cannot increase resolving power; however, approaches exist across disciplines that leverage the multiplexed experiment to sharpen peak shapes through solely algorithmic means. While graphically appealing, caution is warranted using these approaches to avoid over interpreting analytical results. Therefore, if we wanted to increase resolving power and resolution of these peaks, multiplexing is not the answer, but instead changing other instrument parameters such as pressure and reduced electric field strength.^3,41^

**Table 3:** Resolving Powers, Absolute Signal (nA), and SNR for Multiple Peaks of Linalool, Cinnamaldehyde, and Ethyl Butyrate, for Two of the Tested Sequences Plus the 50 Signal Average Measurement^a^

			B8O10			B10O5			50 signal averages		
analyte	peak	*K*_0_ (cm^2^ V^–1^ s^–1^) at 80 Td	*R*_*p*_	absolute signal (nA)	SNR	*R*_*p*_	absolute signal (nA)	SNR	*R*_*p*_	absolute signal (nA)	SNR
linalool	dominant	1.80 ± 0.01	45	1.86	509	46	2.18	521	45	0.60	145
	side	1.73 ± 0.01	63	0.34	93	62	0.41	98	72	0.133	30
cinnamaldehyde	peak 1	2.044 ± 0.02	69	0.35	49	89	0.15	8.8	73	0.31	66
	peak 2	1.902 ± 0.02	97	0.52	93	95	0.67	39	56	0.60	100
	peak 3	1.751 ± 0.02	79	3.00	412	67	3.69	213	83	2.7	577
ethyl butyrate	peak 1	1.895 ± 0.001	41	0.21	57	46	0.27	25	66	0.22	50
	peak 2	1.811 ± 0.001	77	2.73	543	66	3.23	305	83	2.1	631

a The peaks used to obtain these values are shown in Figure S10.

### SNR, Artifacts, and the Ion Gate/Faraday Plate Relationship

**Figure 5:**
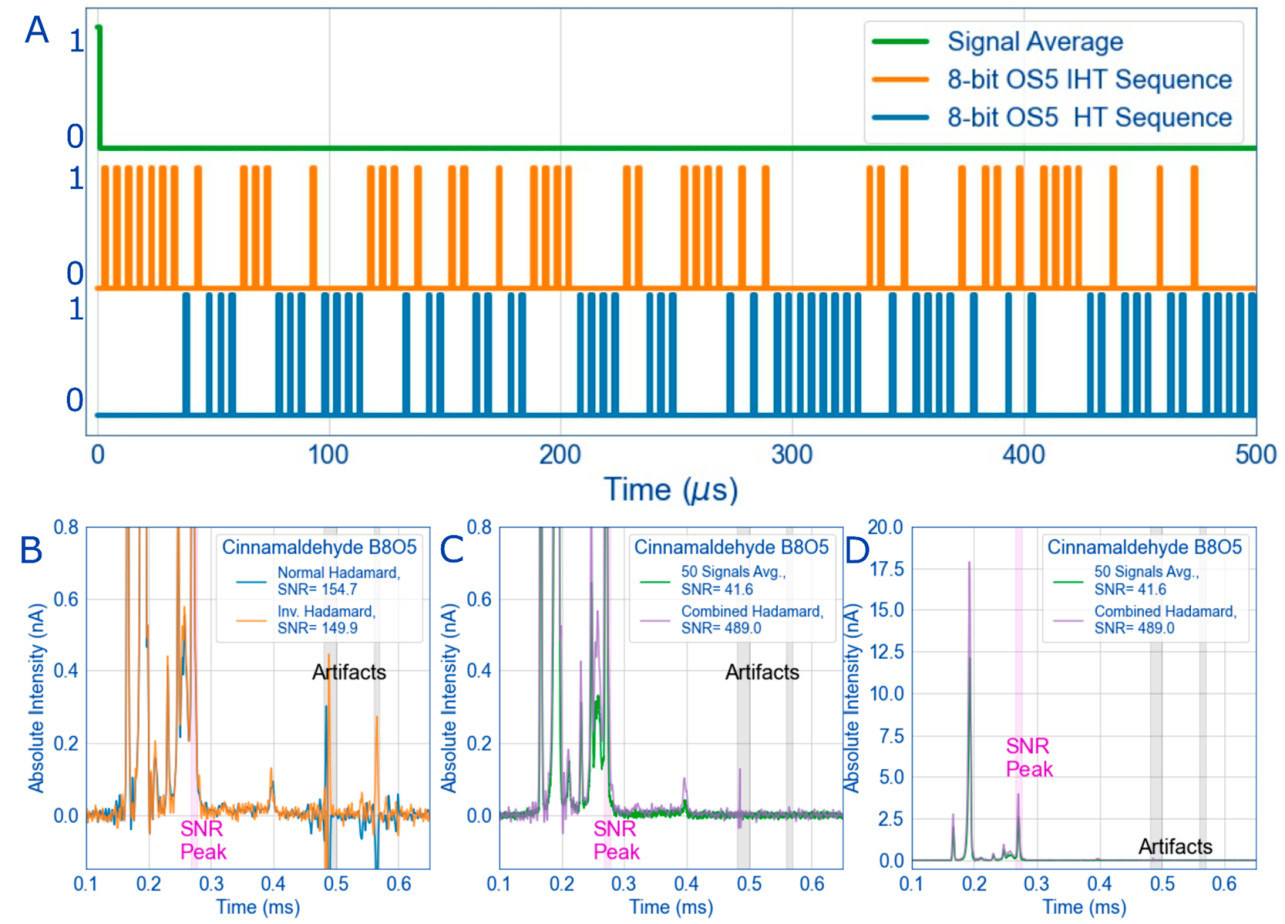
One method to eliminate artifacts, as described by Hong et al., is the implementation of inverse Hadamard in addition to normal Hadamard.^47^ This experiment takes the original PRBS and replaces all the 0’s with 1’s and vice versa; an example of this is observed in the top figure for the first 500 points of the sequence. Oversampling or overstretching is then applied after the inversion (A). Then the Hadamard experiment with 70 ppb_v_ cinnamaldehyde is performed twice: once with the normal HT sequence and again with the IHT sequence. Artifacts in the spectra then mirror each other in the spectra along the baseline when comparing the HT and IHT deconvoluted spectra (B). By combining the HT and IHT spectra in the combined HT spectra (purple dashed), the artifacts are eliminated and the SNR is greatly improved to the signal averaged spectra (C and D). Experimental instrument parameters are listed in Table 1 and sequence abbreviations are in Table 2.

While the signal gains from multiplexing on the HiKE-IMS are significant, in some spectra (Figure 5) the usual drawback of the Hadamard transform technique appears in the form of artifacts that severely impact the SNR. It should be emphasized that artifacts are somewhat of a misnomer, and arise from misplaced expectations between matrix algebra and real data.^2^ In deconvolution, the inverted matrix and circular deconvolution algorithm expects a perfect matrix of 0’s and 1’s, but real data (when normalized) is often a range between these values. There is a rich history in the field of spectroscopy characterizing these sorts of errors, including those named after the authors Tai and Tate,^48−50^ but in IMS, these sorts of errors arise from situations such as when the ion current is unstable or situations that cause imperfect Gaussian peaks.^2^ In HiKE-IMS, the corona current is stable during the mobility experiments and the 1 μs gate pulse width results in highly ideal Gaussian peaks. Therefore, the most likely cause of these artifacts is due to imperfect removal of the effect of capacitive coupling of the ion gate on the Faraday plate. Careful examination of the artifacts in Figure 5 looks suspiciously similar to the effects of gate pulse capacitive coupling in the signal average spectra at 0 ms. Luckily, a few different techniques have been developed to mitigate the errors caused by artifacts.^22,47,51^ The most recent effort is from Clowers et al. using a masked multiplexed approach, where random errors are seeded into the A matrix before deconvolution.^51^ Upon transform, the errors are propagated throughout the spectra and the real ion signals are immediately apparent. However, this method can be computationally expensive, especially as bit size and matrix size increase.^51^

The method we chose to eliminate these so-called artifacts is described by Hong et al. using inverted Hadamard sequences (IHT).^47^ With this method, the original PRBS is inverted then the IMS experiment is performed twice: once with normal HT, and once with inverted HT (Figure 5A). Upon transform, the artifacts mirror each other in the spectra whereas the real ion signal is constant. In Figure 5B, some “artifacts” are visible at longer drift times for the spectra of cinnamaldehyde and when the IHT method is used, the artifacts are obvious because they are mirrored at the baseline. By averaging the normal Hadamard spectra from the inverted spectra, the artifacts are “subtracted out” and eliminated, leaving the ion signal and further increasing the SNR of the demultiplexed Hadamard spectra. In Figure 5, the SNR gain after this data treatment is a factor of 6.2. While using this method best maximizes SNR gains from multiplexing, the obvious drawback is the need to perform the experiment twice (once with IHT and HT), effectively doubling the length of the experiment.

**Figure 6:**
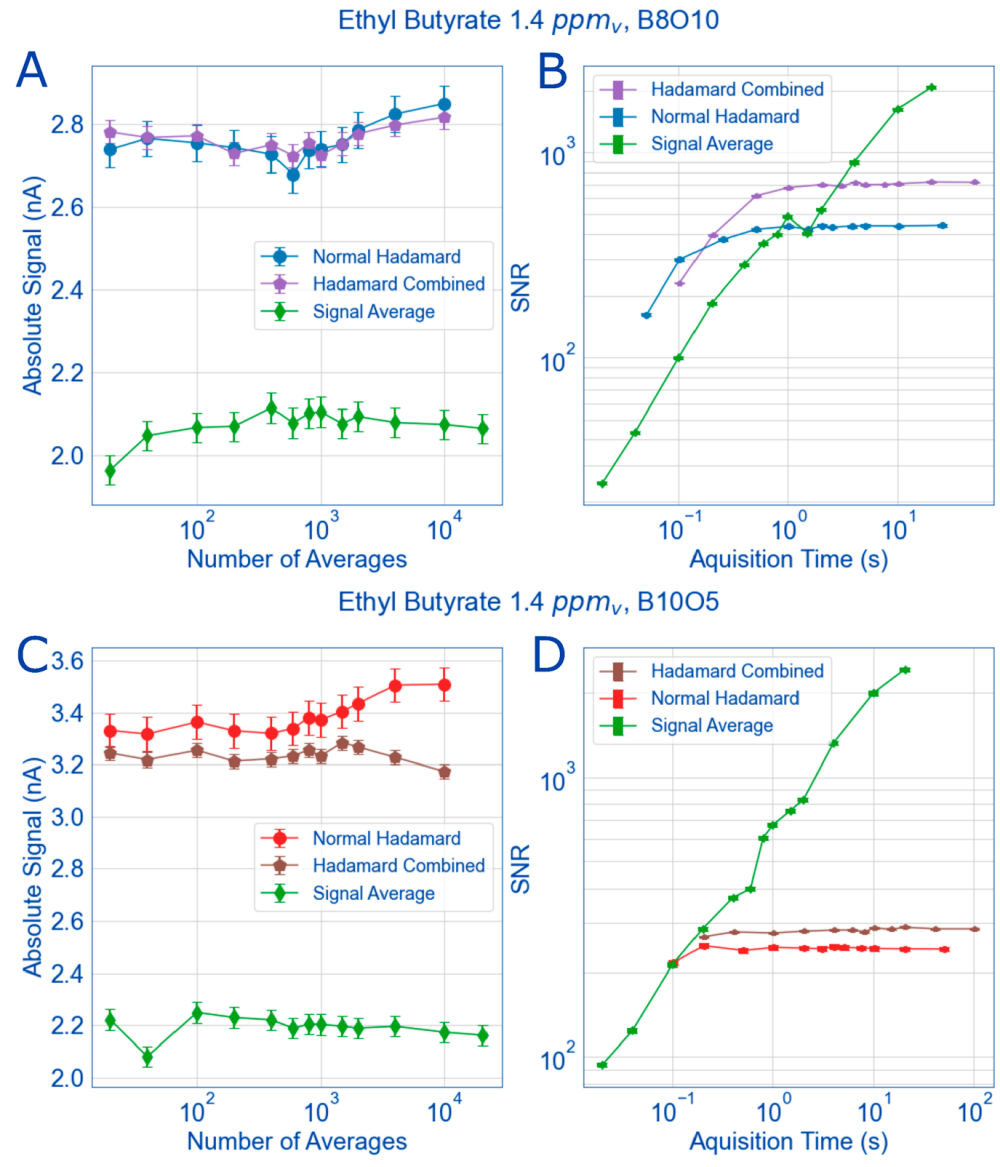
For the primary ion peak of ethyl butyrate (1.4 ppm_v_), the absolute signal of the monomer ion peak (1 standard deviation for error) is plotted against the number of averages taken (20 to 20,000, A and C) or equivalent acquisition time (B and D). The signal from this peak remains relatively constant within error no matter the number of averages taken; however, the multiplexed spectra always have more signal than the signal averaged spectra (A) and the 10-bit sequence has 20% more signal than the 8-bit sequence (C). However, when plotting the SNR against the acquisition times associated with the number of averages from A and C, the challenges with HiKE-IMS multiplexing become obvious (B and D). Careful selection of the number of bits and oversampling with a small number of averages results in an improvement of SNR of up to a factor of 10 with the fewest number of averages (B). Otherwise, if the number of bits in the sequence is maximized and oversampling minimized for ion throughput, noise from the ion gating event negates the gain in signal and results in longer acquisition times and reduced SNR compared to signal averaging (D). Experimental instrument parameters are listed in Table 1 and sequence abbreviations are in Table 2.

Finally, to characterize the balance of fast acquisition time, number of bits of the sequence, oversampling, and theoretical gain, ethyl butyrate was measured using traditional signal average mode, a 10-bit 5× oversampled Hadamard sequence, and an 8-bit 10× oversampled Hadamard sequence (Figure 6). When comparing the absolute signal from the ethyl butyrate peak width between normal Hadamard, combined normal Hadamard and inverse Hadamard (the method described above with Figure 5), and the signal average mode, the intensity of the peak remains mostly constant no matter how many averages are taken (A and C). Additionally, there is more signal in the 10-bit sequence than the 8-bit sequence by a minimum of 20%. This is unsurprising and agrees with the results presented so far. It is, however, interesting that the combined Hadamard spectra has a lower overall signal than the normal Hadamard spectra for the 10-bit sequence (C), but this is because the inverse Hadamard spectra also has a slightly lower signal (Figure S10). It should be noted that the reduced signal for the inverse Hadamard spectra is likely experimental variation and not related to space charge effects which some literature about inverse IMS discuss.^47,52,53^ The reason we can rule out space charge effects is because our inverse Hadamard is oversampled (i.e., there extra space between the ion pulses) and therefore, not the same as a signal averaged inverse IMS experiment with a corona source. If space charge effects exist in inverse Hadamard experiments, they would show up in a normal Hadamard experiment too, since in both, the duty cycle approaches 50%. While there is high ion current in the reaction region of the HiKE-IMS where space charge effects can occur, once the ion gate pulses, less than 1/1000th of those ions are let through in signal average mode. With oversampled multiplexing, that fraction increases to 1/10th at most which is still significantly smaller than the ion current in the reaction region and no space charge effects will happen during the mobility experiment.

Upon converting the absolute signal of ethyl butyrate into a signal-to-noise ratio (here defined as the signal of the ethyl butyrate peak graphed in Figure 6A and 6C, divided by the standard deviation of the noise between 1 and 5 ms), the challenges of implementing Hadamard multiplexing on HiKE-IMS become obvious (Figure 6B and 6D). First, the maximum gain in Hadamard multiplexing is limited only by the number of bits in the sequence, so to achieve the theoretical maximum SNR, the number of bits should be maximized. This lengthens the sequence and thus the acquisition time. Next, because the nature of the IMS experimental time scale is linear, the first Hadamard spectrum collected is unusable because it is physically impossible for ions pulsed at the end of the sequence to show up in the first few points of the first mobility spectrum. Therefore, the second spectrum is the first usable spectrum for the correct deconvolution of HT-IMS spectra, which further lengthens the acquisition time. This means the first spectrum must be discarded or some degree of averaging must happen when multiplexing. Additionally, for HiKE-IMS, and other IMS systems that use the tristate ion shutter, oversampling must be used which further lengthens the sequence and lowers the duty cycle. For example, for a 10-bit 5 times over sampled sequence in HiKE-IMS with 1 μs GPW, the acquisition time for one spectrum is 10.23 ms (5.115 ms × 2, because the first spectrum must be discarded). The 10.23 ms does not include the time for a blank spectrum (double that time, so 20.46 ms including the blank) or the time for an inverted spectrum (double the time for the blank, so 40.92 ms for Hadamard and inverted Hadamard plus blanks and discarding the first spectrum). However, the drift times in the HiKE-IMS are exceedingly short due to the reduced pressure and high reduced electric field strengths resulting in drift times of less than 0.5 ms (Figure 4A and 4B, Figure 5C). During the time it takes to obtain one multiplexed spectrum plus the blanks, 40 signal-averaged spectra could be measured (80 signal averaged spectra if using combined Hadamard and inverse Hadamard). During this time, the signal averaged spectra have 40 (or 80) ion gate pulsing events, whereas the Hadamard would have 512, resulting in the increased signal. However, even when subtracting out the blank spectra, and using the combined Hadamard and inverse Hadamard spectra, artifacts from the ion gate pulse are not completely eliminated and results in a lower or constant SNR for the multiplexed spectra than all signal-averaged spectra when accounting for equivalent acquisition time (Figure 6D).

It should be further emphasized that the SNR gain relative to theory is reduced precisely because a Faraday plate is used, which strongly capacitively couples to the ion gate pulsing and introduces a systematic error. Hadamard and other multiplexing techniques increase SNR primarily by evenly distributing random errors throughout the spectra, but will not necessarily improve the SNR for systematic errors such as the effect of capacitive coupling from the ion gate and Faraday plate. In instruments that remove the capacitive coupling between the ion gate and detector (either by switching to a pulsed ion source and removing the gate, or coupling the IMS to a mass spectrometer), the systemic errors are eliminated and theoretical SNR is further improved. This improvement is exemplified in experiments performed using HT-IMS-TOF-MS from Clowers et al. that show a significant increase in SNR using only a 5-bit PRBS.^20^ The difference between these two experiments again is the use of a Faraday plate compared to an IMS-TOF-MS. Furthermore, the SNR of the HiKE-IMS is already high when operated in signal average mode (over 2000 with 1024 signal averages here), significantly higher than that shown by Hong et al. (maximum is 95 for SA, 157 for HT).^47^ While multiplexing improves ion signal for HiKE-IMS, further improvements to SNR and acquisition time from multiplexing implementation on a stand-alone system (i.e., no MS) are likely application specific and situational, such as comparing a small number of averages with a highly oversampled sequence (Figure 6B).

## Conclusion

An open source, low cost Teensy 4.1 microcontroller was used to implement Hadamard multiplexing on the HiKE-IMS with no additional instrument modifications for the first time. The operation of the Teensy is highly flexible; the communication via fiber optics and SMA connectors on the mounting board allows this device to be used not only with any IMS instrument from our group, but also with any instrument that uses fiber optics or SMA (or any SMA adapter) to communicate when the ion gating event should occur including those using the pulsers from, e.g., Gracia et al.^54^ While the ease of implementation of multiplexing with a focus on open-source is demonstrated here, expectations need to be bound accordingly when implementing Hadamard multiplexing on a pre-existing system that was not designed for multiplexing. For example, due to the tristate-gating scheme, oversampling must be utilized, and therefore the maximum possible duty-cycle is decreased from 50% down to 10%. Additionally, the ion gating event introduces significant noise into stand-alone systems, like HiKE-IMS, from the influence of capacitive coupling to the Faraday plate, which cannot be completely eliminated. For these reasons, multiplexing on the HiKE-IMS is possible, but the gains are not as high as theoretically possible on other IMS platforms, especially ones utilizing a mass analyzer for detecting the ions. However, additional challenges can be expected when applying multiplexing to commercial systems (both IMS and MS) including bypassing the closed nature of these systems. Commercial low-pressure IMS-MS systems may include an ion trapping region before the mobility cell which may increase the number of ions injected into the drift tube; however, these instruments still have one injection pulse when operated in signal average mode that can be increased by multiplexing. Multiplexing TOF-MS may be possible using the Teensy, since the clock is accurate within the nanosecond range, but other strategies may be necessary for higher resolution mass spectrometry systems.

With HiKE-IMS, when using a 10-bit, oversampled 5 times sequence, the absolute gain in analyte signal approaches a maximum of a factor of 4 and the SNR gain approaches a factor of 10 only when using the inverted HT technique as described by Hong et al. and under specific sequence/oversampling selection.^47^ Through this initial effort, the groundwork is established for improving duty-cycle from <0.1% up to 10% by using the Teensy coupled with IMS. This increase in ion current using Hadamard transform is highly beneficial for any future couplings of drift tube IMS to mass spectrometers.

## Data Availability

All Arduino code to control the Teensy and data analysis in Python are also freely available on github (github.com/bhclowers/DAMS).

## Supporting Information Available

All the Teensy firmware, Python Code for sequence generation and deconvolution, and an example data set for cinnamaldehyde (ZIP)
A brief tutorial of Teensy operation and additional figures (PDF)
