# Supplementary material for: Implementation of an Open-Source Multiplexing Ion Gate Control for High Kinetic Energy Ion Mobility Spectrometry (HiKE-IMS)
Source: J Am Soc Mass Spectrom. 2023 Jun 5;34(7):1283–94. doi: 10.1021/jasms.3c00013 (PMC10326917; doi:10.1021/jasms.3c00013)
Supplement: Supplementary file 2 — js3c00013_si_002.pdf [file js3c00013_si_002.pdf]

Supporting Information for Implementation of an Open-Source Multiplexing Ion Gate Control for High Kinetic Energy Ion Mobility Spectrometry (HiKE-IMS)

Cameron N. Naylor[1*], Brian H. Clowers[2], Florian Schlottmann[1], Nic Solle[1], Stefan Zimmermann[1]
1: Leibniz University Hannover, Institute of Electrical Engineering and Measurement Technology, Department of Sensors and Measurement Technology, 30167 Hannover Germany
2: Washington State University, Department of Chemistry, Pullman WA 99164 USA
*Corresponding Author: naylor@geml.uni-hannover.de

Table of Contents:

What Happens when Capacitive Coupling Isn't Mitigated: S-2
Figure S1:Signal Average Spectra + Blank S-2
Figure S2: Raw Multiplexed Spectra S-3
Figure S3: No Capacitive Coupling Removed S-4
Teensy Mounting Board:..S-5
Figure S4: Board Assmebly Diagram S-5
Table S1:Board Parts Table S-5
Teensy Operation Tutorial: S-6
Figure S5: File Selection..S-6
Figure S6: List of Commands S-7
Figure S7: Pulse/Bin Length Settings S-8
Figure S8: Adjusting Spectra Time S-9
Figure S9:ADQ Number of Samples..S-10
Figure S10: Spectra Used for Table 3 ....S-11
Figure S11: Ethyl Butyrate Spectra S-12

What Happens when Capacitive Coupling Isn’t Mitigated:

All of the following spectra are using α-pinene and are the same data presented in Figures 2 & 3. However, by doing this short exercise in data analysis, we can show the effect of capacitive coupling and what happens when you don’t mitigate it.

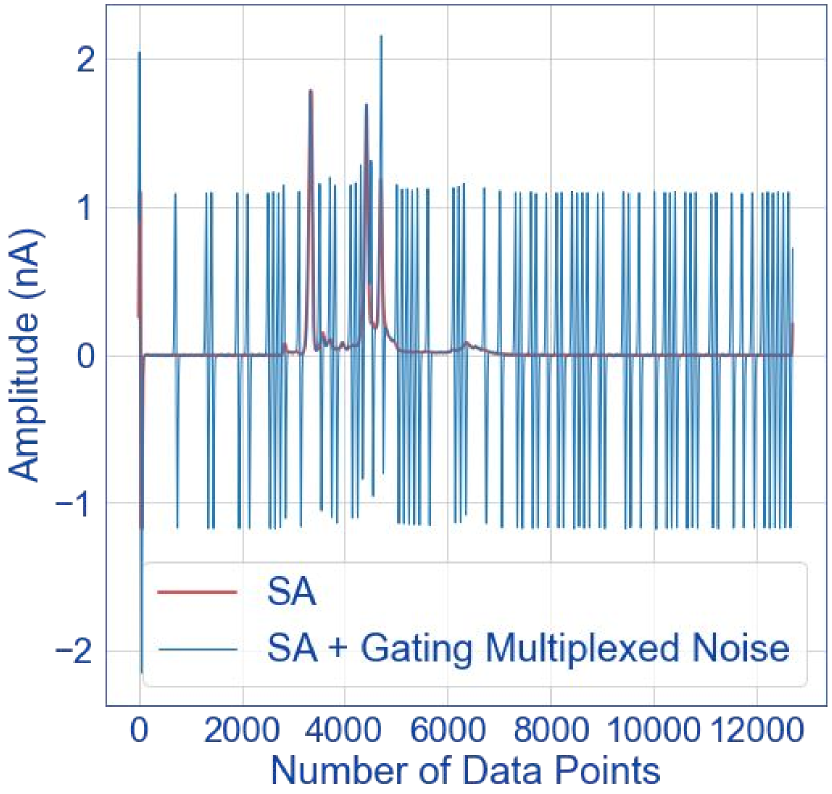

Figure S1: On the left is the blank spectra of the multiplexing pulsing scheme with the corona source turned off (blue) added onto the signal averaged spectra (red). Here the signal average spectrum represents the resulting ion mobility spectrum from just the first gate pulse. However, every time the gate pulses, the effect of capacitive coupling between the ion gate and Faraday plate are nearly equal in intensity to the actual ion signal resulting in an interference pattern that will cause errors in the transform spectra if not mitigated.

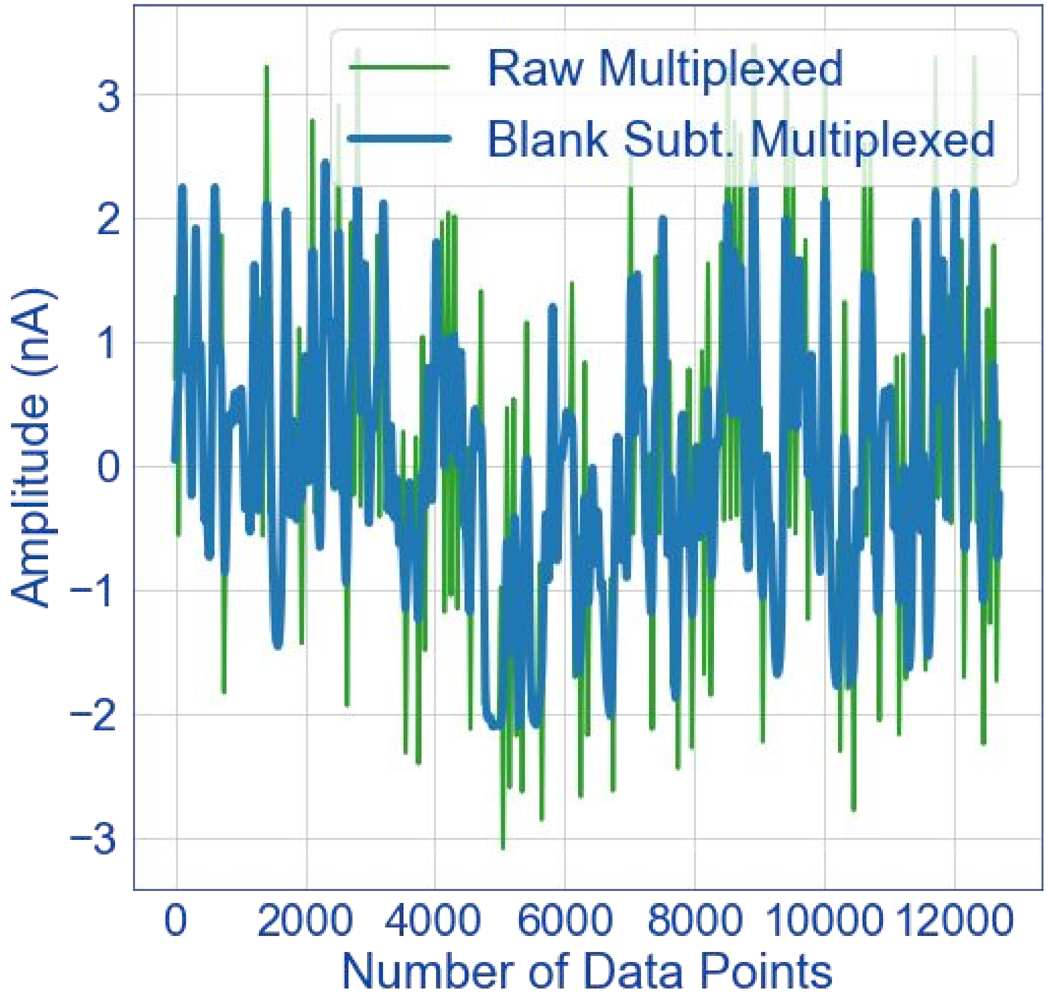

Figure S2: Is the full graph of the Raw Multiplexed spectra compared to the blank subtracted spectra shown in Figure 2B.

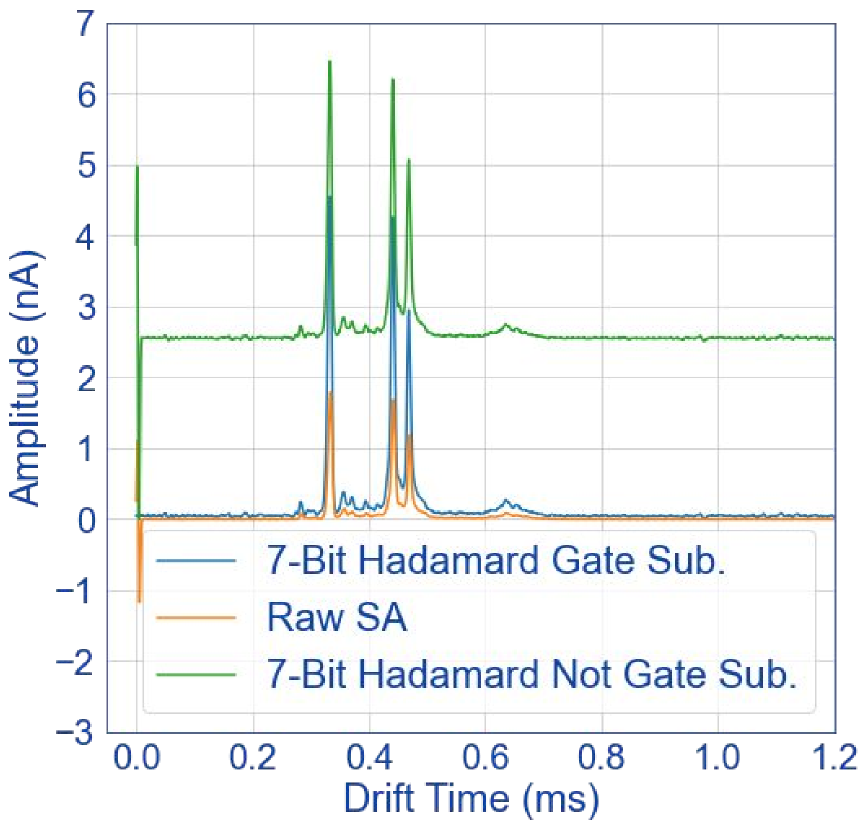

Figure S3: If the blank spectrum is not subtracted before deconvoluting the spectra (green), the following happens when comparing to the signal averaged (orange) and blank-subtracted deconvoluted Hadamard spectra (blue): 1) a constant offset is introduced into the data. The baseline of the green trace at 0.01 V was not added for aesthetic graphic purposes, but instead is a result of the constant noise from the capacitive coupling adding additional signal all throughout the spectra. 2) At first glance, the spectra are transformed correctly (the little spikes in the baseline are preserved in both the blue and green spectra), however, on closer examination, the signal gains are not the same. In the blank subtracted blue trace (Also visible in Figure 2C), each of the 3 most abundant peaks have maximums at 0.017V, 0.016V, and 0.011V. When accounting for the constant offset, the green trace has maximum intensities of 0.014V, 0.013V, and 0.009V respectively. 3) The effect of capacitive coupling between the ion gate and Faraday plate is present for all ion mobility spectra at the 0.0ms time point. However, in the non-blank subtracted multiplexed spectra, this effect is much more prominent (intensities are nearly doubled from the signal averaged and blank-subtracted deconvoluted spectra).

Teensy Mounting Board:

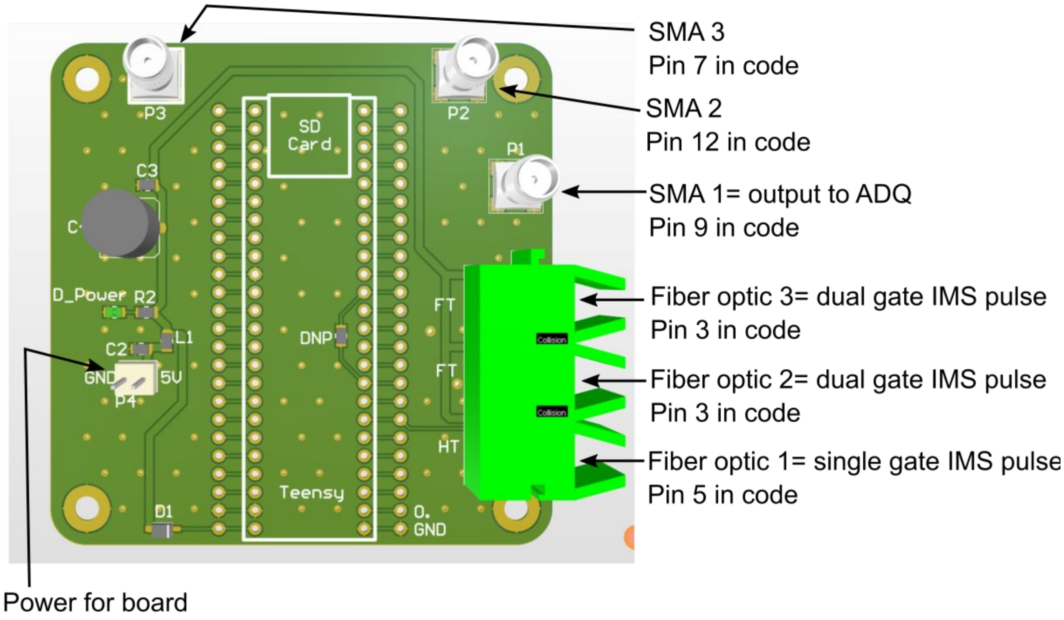

Figure S4: A diagram of the Teensy mounting board (all files included in supplemental) and the additional parts required for assembly. Not listed on the board, you will need one (or two for FT) fiber optic cables to send the pulsing sequence to the ion gate controller/pulser, header pins to connect the Teensy to the mounting board and the breakout lane to troubleshoot pin signals from the Teensy, a Teensy 4.1, and a micro-USB cable to connect the Teensy to a computer.

Table S1: A list of parts for the Teensy Mounting board required for assembly

	Bottom of Board		
**Board Key**	**Part**	**Supplier**	**Catalogue Number**
C5, C6	Do Not Place		
R3, R4	0 R		
U1	SN74LVC2G17DBVR Dual Schmitt Trigger Buffer	Farnell	3119647
	Top of Board		
LWL1, LWL2, LWL3	AFBR-1624Z LWL transmitter TTL 3.3-5V, 50MBit	Farnell	2213640
P1, P2, P3	SMA	Farnell	1342652
C2, C3	10µF 35V	Farnell	2547034
C4	330µF 35V	Mouser	661-EMZR350ARA331MHA
D_Power	SMD LED green	Reichelt	SMD-LED 0805 GN
L1	10µH	Mouser	810-MLZ2012N100L
R2	4K7		
D1	1N4148W	Farnell	2675147
P4	2-pin jumper		
	Socket Header 24x1		

Teensy Operation Tutorial:

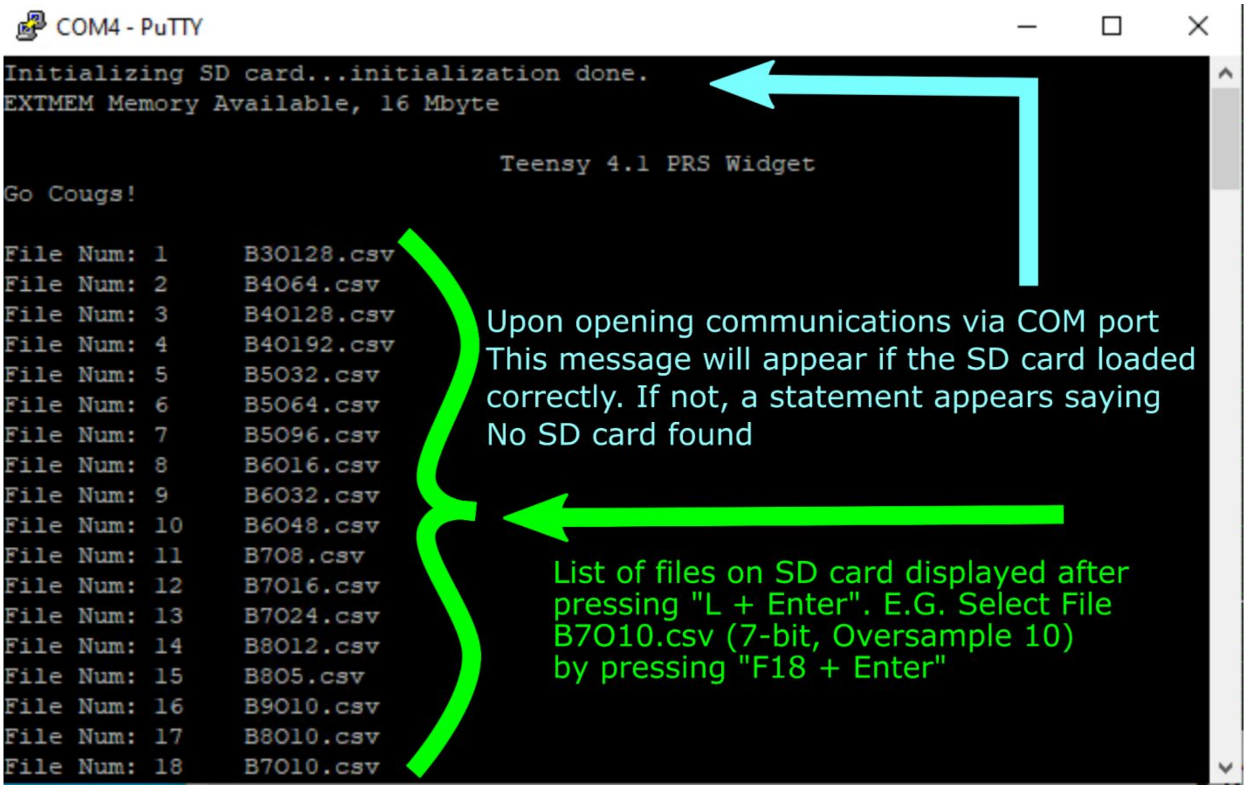

Figure S5: The GUI from Putty is displayed with the outputs of initialization of the Teensy. Sometimes the SD card is not found upon booting, and in this case, the Teensyduino needs to be rebooted by pressing the button on top of the microcontroller. After the SD card initializes, the files can be loaded.

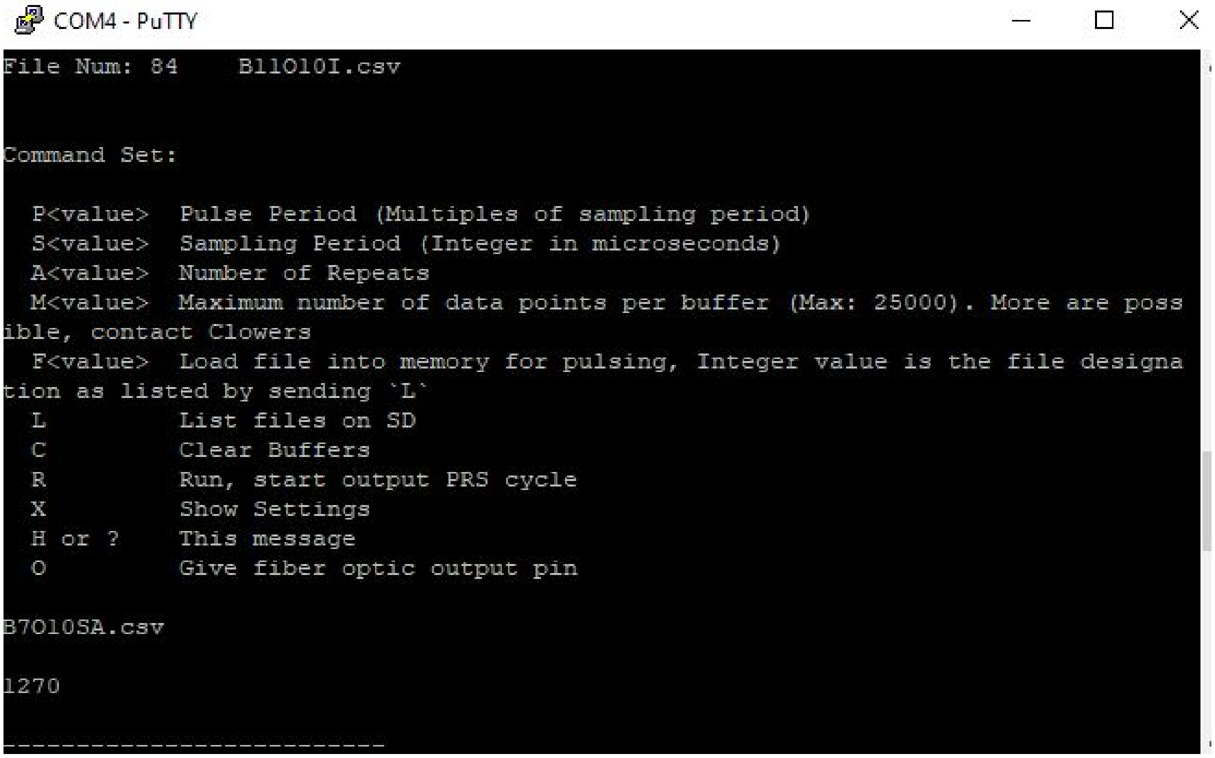

Figure S6: By pressing “H + enter” a “cheat sheet” of commands for the Teensy are displayed. By pressing one of these letters (and an integer value) plus Enter, the command is executed. To implement any IMS experiment, the gate pulse width must be set first (see Figure S3).

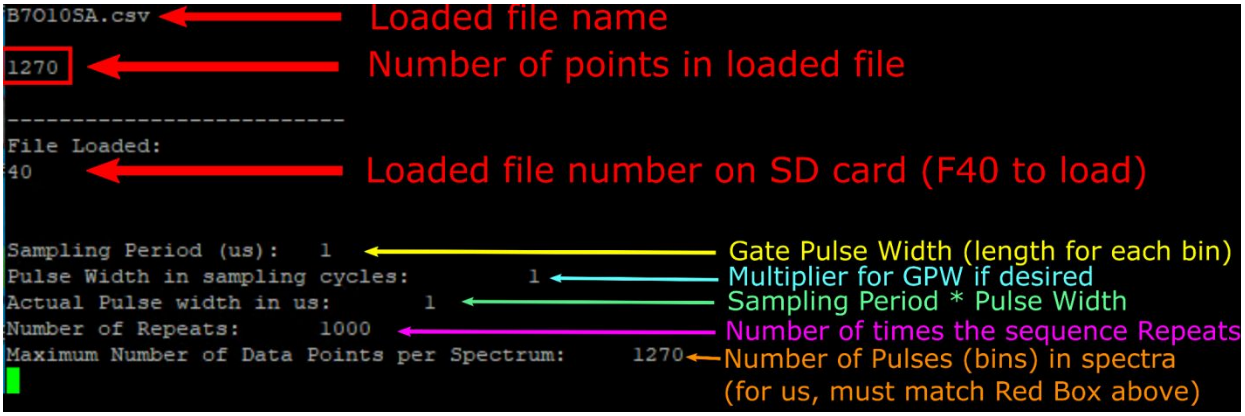

Figure S7: By pressing "L + enter" all files on the SD card are displayed. Upon loading one of them using "F<number> + Enter" (here, F40), the name of the selected file and number of points in the file are displayed. The number of points are important for setting up the gate pulsing event. Next, by pressing "X + Enter" all gate pulsing details are listed. First is the sampling period, or default gate pulse width (changed by pressing "S<int> + Enter"). This value can be extended by pressing "P<value> + Enter" and is displayed in the next line. The third line (green) is the multiplication of both of these values and your actual gate pulse width. For us, everything must be "1" because of the 1 microsecond gate pulse width in the HiKE-IMS. "A<value> + Enter" is the number of times the Teensy repeats the measurement, or the number of averages. For Hadamard, this number must be greater than 1. Finally displayed is the number of data points in the spectrum, changed with "M<value> + Enter". This value must match the number of data points in the loaded file at minimum. Once all settings are input, and the sequence is loaded, press "R+ Enter" to run the sequence and start the experiment.

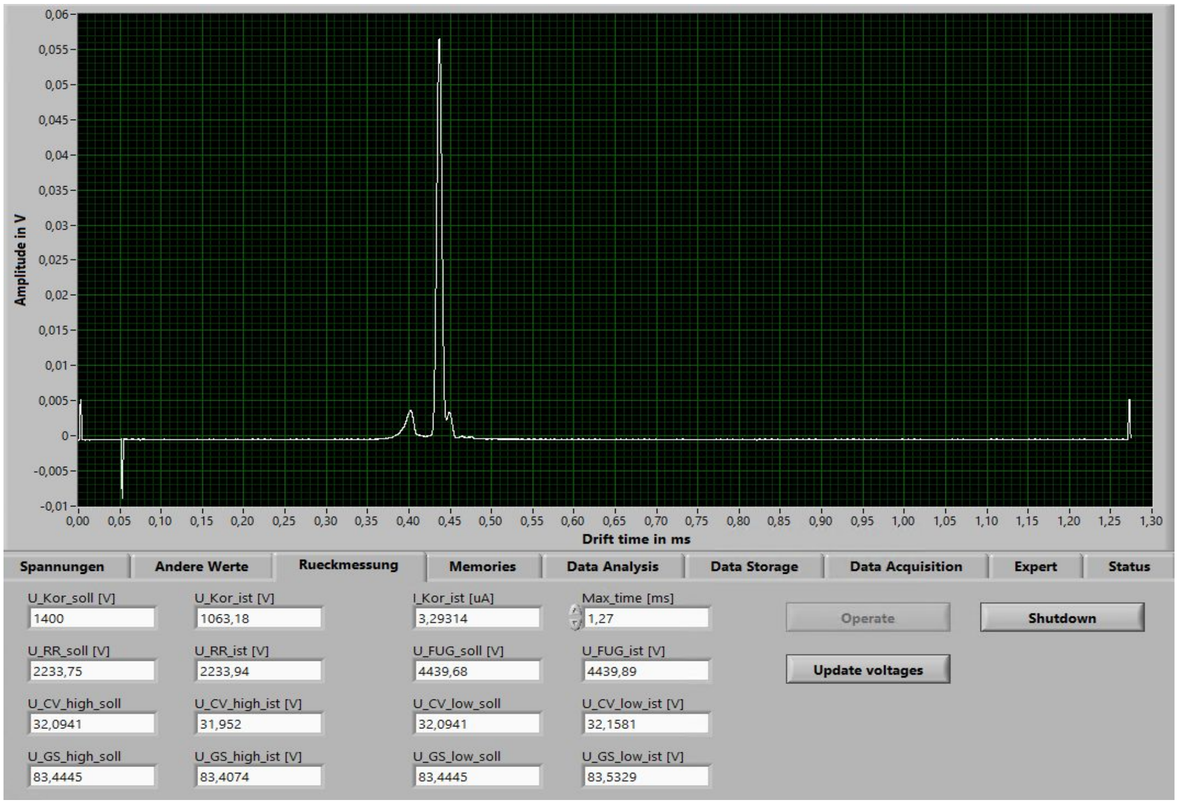

Figure S8: Screenshot of the Meastool software to operate the HiKE-IMS. An additional parameter has been added that specifies the time of data collection to accommodate the precise number of data points needed for Hadamard multiplexing (in this case, 7-bit 10x oversampled, 1270 µsec long sequence). The spectra displayed is a signal averaged reactant ion spectra. Additionally, note the pulses at 0ms and 0.05ms which are the effect of capacitive coupling of the ion gate to the detector from the injection pulse.

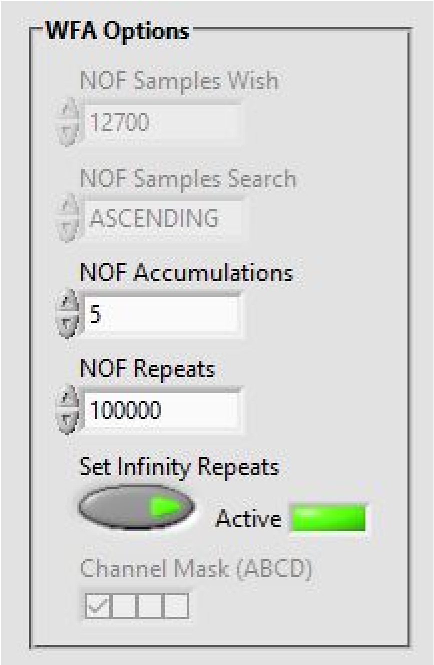

Figure S9: Screenshot of the ADQ software where the exact number of data points is entered under NOF Samples Wish. In this case, the number 12700, is for a 7-bit 10 x oversampled sequence (length 1270, 1.27 ms), and multiplied by 10 to ensure a Gaussian peak shape.

Spectra Used for Table 3:

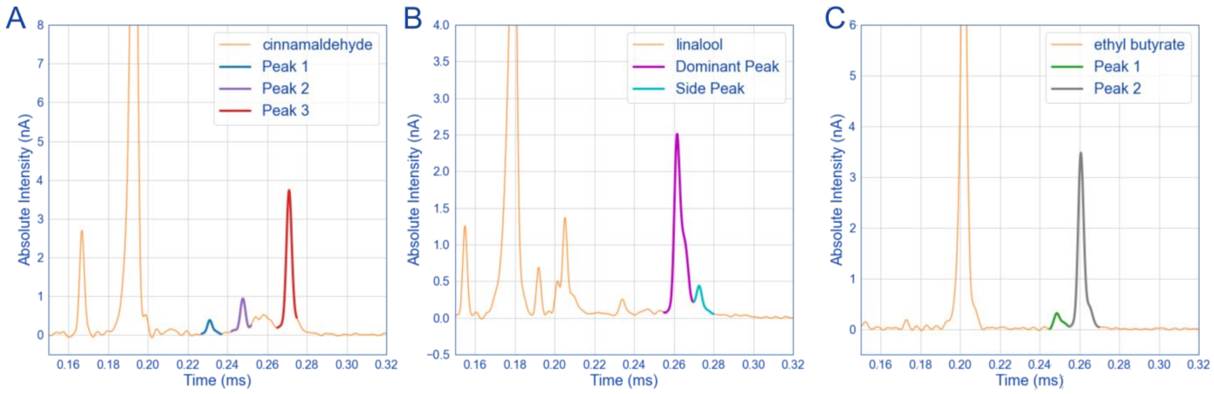

Figure S10: Shown are the inverse Hadamard subtracted (B10O5) spectra for cinnamaldehyde (A), linalool (B), and ethyl butyrate (C). The highlighted peaks for each analyte were used to calculate the resolving powers, absolute signals, and SNR for Table 3.

Ethyl Butyrate Spectra:

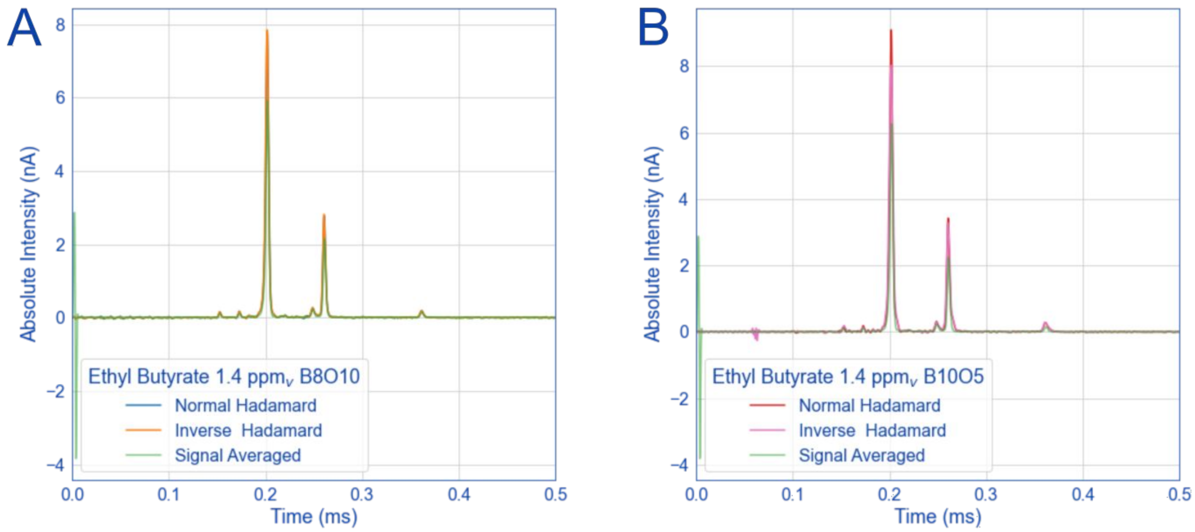

Figure S11: The demultiplexed and SA spectra for Figure 6 are shown. An 8-bit, 10-times oversampled spectrum of ethyl butyrate with normal and inverse Hadamard as well as signal averaged data is plotted (A). Additionally, a 10-bit, 5-times oversampled spectrum of ethyl butyrate with normal and inverse Hadamard is plotted with the signal averaged data (B). The 8-bit, 10 times overesampled sequence (A) shows almost identical signal gain of normal and inverse Hadamard compared to the 10-bit, 5 times oversampled sequence (B) where the inverse multiplexed spectra has less signal gain than the normal Hadamard.
