# Supplementary material for: Implementation of an Open-Source Multiplexing Ion Gate Control for High Kinetic Energy Ion Mobility Spectrometry (HiKE-IMS)
Source: J Am Soc Mass Spectrom. 2023 Jun 5;34(7):1283–94. doi: 10.1021/jasms.3c00013 (PMC10326917; doi:10.1021/jasms.3c00013)
Supplement: Supplementary file 1 — js3c00013_si_001.zip [file js3c00013_si_001.zip › js3c00013_si_001/Teensykicad/Pads.html]

 


```
Dump: MAM_PCB-PcbDoc
  


02

  


02 00 00 00


01


31

  


01 00 00 00


00

  


05 00 00 00


04 7C 26 7C 30

  


01 00 00 00


00

  


C2 00 00 00


01


0C 00


0A 00


FF FF


09 00


FF FF FF FF


09 7C 89 01


65 BE 3F 01


0D 69 08 00


79 35 07 00


0D 69 08 00


79 35 07 00


0D 69 08 00


79 35 07 00


00 00 00 00


02


02


02


00 00 00 00 00 80 66 40


01


00


00


00 00 00 00 00


A0 86 01 00


04


00


A0 86 01 00


40 0D 03 00


40 0D 03 00


00 00 00 00


40 9C 00 00


00


00 00 01 01 01 01


01


01


01 00 00


00 00 00 00 00 00 00 00


01 00 00 01 00 00 00 40 9C 00 00 00 B4 CC 36 A4 12 8B DD 48 97 18 E1 C6 BA A6 65 4C 33 82 41 85 38 79 42 40 83 79 2B 1B 7D 37 65 33 00 00 00 00 FF FF FF 7F FF FF FF 7F 00 01 12 00 00 00 00 00 00 00 00 00 00 00 01 03 00 00 00 00 00 00 00 00

  


00 00 00 00

  


02

  


02 00 00 00


01


32

  


01 00 00 00


00

  


05 00 00 00


04 7C 26 7C 30

  


01 00 00 00


00

  


C2 00 00 00


01


0C 00


16 00


FF FF


09 00


FF FF FF FF


09 7C 89 01


9A 8E 4A 01


0D 69 08 00


79 35 07 00


0D 69 08 00


79 35 07 00


0D 69 08 00


79 35 07 00


00 00 00 00


02


02


02


00 00 00 00 00 80 66 40


01


00


00


00 00 00 00 00


A0 86 01 00


04


00


A0 86 01 00


40 0D 03 00


40 0D 03 00


00 00 00 00


40 9C 00 00


00


00 00 01 01 01 01


01


01


01 00 00


00 00 00 00 00 00 00 00


01 00 00 01 00 00 00 40 9C 00 00 00 B4 CC 36 A4 12 8B DD 48 97 18 E1 C6 BA A6 65 4C 33 82 41 85 38 79 42 40 83 79 2B 1B 7D 37 65 33 00 00 00 00 FF FF FF 7F FF FF FF 7F 00 01 12 00 00 00 00 00 00 00 00 00 00 00 01 03 00 00 00 00 00 00 00 00

  


00 00 00 00

  


02

  


02 00 00 00


01


32

  


01 00 00 00


00

  


05 00 00 00


04 7C 26 7C 30

  


01 00 00 00


00

  


C2 00 00 00


01


0C 00


10 00


FF FF


0D 00


FF FF FF FF


AC D3 E0 00


18 D3 58 01


0D 69 08 00


79 35 07 00


0D 69 08 00


79 35 07 00


0D 69 08 00


79 35 07 00


00 00 00 00


02


02


02


00 00 00 00 00 E0 70 40


01


00


00


00 00 00 00 00


A0 86 01 00


04


00


A0 86 01 00


40 0D 03 00


40 0D 03 00


00 00 00 00


40 9C 00 00


00


00 00 01 01 01 01


01


01


01 00 00


00 00 00 00 00 00 00 00


01 00 00 01 00 00 00 40 9C 00 00 00 B4 CC 36 A4 12 8B DD 48 97 18 E1 C6 BA A6 65 4C 33 82 41 85 38 79 42 40 83 79 2B 1B 7D 37 65 33 00 00 00 00 FF FF FF 7F FF FF FF 7F 00 01 12 00 00 00 00 00 00 00 00 00 00 00 01 03 00 00 00 00 00 00 00 00

  


00 00 00 00

  


02

  


02 00 00 00


01


31

  


01 00 00 00


00

  


05 00 00 00


04 7C 26 7C 30

  


01 00 00 00


00

  


C2 00 00 00


01


0C 00


31 00


FF FF


0D 00


FF FF FF FF


E1 A3 EB 00


18 D3 58 01


0D 69 08 00


79 35 07 00


0D 69 08 00


79 35 07 00


0D 69 08 00


79 35 07 00


00 00 00 00


02


02


02


00 00 00 00 00 E0 70 40


01


00


00


00 00 00 00 00


A0 86 01 00


04


00


A0 86 01 00


40 0D 03 00


40 0D 03 00


00 00 00 00


40 9C 00 00


00


00 00 01 01 01 01


01


01


01 00 00


00 00 00 00 00 00 00 00


01 00 00 01 00 00 00 40 9C 00 00 00 B4 CC 36 A4 12 8B DD 48 97 18 E1 C6 BA A6 65 4C 33 82 41 85 38 79 42 40 83 79 2B 1B 7D 37 65 33 00 00 00 00 FF FF FF 7F FF FF FF 7F 00 01 12 00 00 00 00 00 00 00 00 00 00 00 01 03 00 00 00 00 00 00 00 00

  


00 00 00 00

  


02

  


02 00 00 00


01


31

  


01 00 00 00


00

  


05 00 00 00


04 7C 26 7C 30

  


01 00 00 00


00

  


C2 00 00 00


01


0C 00


31 00


FF FF


18 00


FF FF FF FF


62 C9 E8 00


D0 19 A3 00


79 35 07 00


87 34 04 00


79 35 07 00


87 34 04 00


79 35 07 00


87 34 04 00


00 00 00 00


02


02


02


00 00 00 00 00 00 00 00


01


00


00


00 00 00 00 00


A0 86 01 00


04


00


A0 86 01 00


40 0D 03 00


40 0D 03 00


00 00 00 00


40 9C 00 00


00


00 00 01 01 01 01


01


01


01 00 00


00 00 00 00 00 00 00 00


01 00 00 01 00 00 00 40 9C 00 00 00 56 D4 9A 0A 0A 89 EC 41 93 83 C3 B1 CC 9C 39 9A 33 82 41 85 38 79 42 40 83 79 2B 1B 7D 37 65 33 00 00 00 00 FF FF FF 7F FF FF FF 7F 00 01 12 00 00 00 00 00 00 00 00 00 00 00 01 03 00 00 00 00 00 00 00 00

  


00 00 00 00

  


02

  


02 00 00 00


01


32

  


01 00 00 00


00

  


05 00 00 00


04 7C 26 7C 30

  


01 00 00 00


00

  


C2 00 00 00


01


0C 00


11 00


FF FF


18 00


FF FF FF FF


96 03 FF 00


D0 19 A3 00


79 35 07 00


87 34 04 00


79 35 07 00


87 34 04 00


79 35 07 00


87 34 04 00


00 00 00 00


02


02


02


00 00 00 00 00 00 00 00


01


00


00


00 00 00 00 00


A0 86 01 00


04


00


A0 86 01 00


40 0D 03 00


40 0D 03 00


00 00 00 00


40 9C 00 00


00


00 00 01 01 01 01


01


01


01 00 00


00 00 00 00 00 00 00 00


01 00 00 01 00 00 00 40 9C 00 00 00 56 D4 9A 0A 0A 89 EC 41 93 83 C3 B1 CC 9C 39 9A 33 82 41 85 38 79 42 40 83 79 2B 1B 7D 37 65 33 00 00 00 00 FF FF FF 7F FF FF FF 7F 00 01 12 00 00 00 00 00 00 00 00 00 00 00 01 03 00 00 00 00 00 00 00 00

  


00 00 00 00

  


02

  


02 00 00 00


01


31

  


01 00 00 00


00

  


05 00 00 00


04 7C 26 7C 30

  


01 00 00 00


00

  


C2 00 00 00


01


0C 00


31 00


FF FF


13 00


FF FF FF FF


02 B1 EC 00


28 9B C3 01


0D 69 08 00


79 35 07 00


0D 69 08 00


79 35 07 00


0D 69 08 00


79 35 07 00


00 00 00 00


02


02


02


00 00 00 00 00 E0 70 40


01


00


00


00 00 00 00 00


A0 86 01 00


04


00


A0 86 01 00


40 0D 03 00


40 0D 03 00


00 00 00 00


40 9C 00 00


00


00 00 01 01 01 01


01


01


01 00 00


00 00 00 00 00 00 00 00


01 00 00 01 00 00 00 40 9C 00 00 00 B4 CC 36 A4 12 8B DD 48 97 18 E1 C6 BA A6 65 4C 33 82 41 85 38 79 42 40 83 79 2B 1B 7D 37 65 33 00 00 00 00 FF FF FF 7F FF FF FF 7F 00 01 12 00 00 00 00 00 00 00 00 00 00 00 01 03 00 00 00 00 00 00 00 00

  


00 00 00 00

  


02

  


02 00 00 00


01


32

  


01 00 00 00


00

  


05 00 00 00


04 7C 26 7C 30

  


01 00 00 00


00

  


C2 00 00 00


01


0C 00


19 00


FF FF


13 00


FF FF FF FF


CD E0 E1 00


28 9B C3 01


0D 69 08 00


79 35 07 00


0D 69 08 00


79 35 07 00


0D 69 08 00


79 35 07 00


00 00 00 00


02


02


02


00 00 00 00 00 E0 70 40


01


00


00


00 00 00 00 00


A0 86 01 00


04


00


A0 86 01 00


40 0D 03 00


40 0D 03 00


00 00 00 00


40 9C 00 00


00


00 00 01 01 01 01


01


01


01 00 00


00 00 00 00 00 00 00 00


01 00 00 01 00 00 00 40 9C 00 00 00 B4 CC 36 A4 12 8B DD 48 97 18 E1 C6 BA A6 65 4C 33 82 41 85 38 79 42 40 83 79 2B 1B 7D 37 65 33 00 00 00 00 FF FF FF 7F FF FF FF 7F 00 01 12 00 00 00 00 00 00 00 00 00 00 00 01 03 00 00 00 00 00 00 00 00

  


00 00 00 00

  


02

  


02 00 00 00


01


31

  


01 00 00 00


00

  


05 00 00 00


04 7C 26 7C 30

  


01 00 00 00


00

  


C2 00 00 00


01


0C 00


14 00


FF FF


0E 00


FF FF FF FF


37 81 F7 00


6C 63 3B 01


0D 69 08 00


79 35 07 00


0D 69 08 00


79 35 07 00


0D 69 08 00


79 35 07 00


00 00 00 00


02


02


02


00 00 00 00 00 80 66 40


01


00


00


00 00 00 00 00


A0 86 01 00


04


00


A0 86 01 00


40 0D 03 00


40 0D 03 00


00 00 00 00


40 9C 00 00


00


00 00 01 01 01 01


01


01


01 00 00


00 00 00 00 00 00 00 00


01 00 00 01 00 00 00 40 9C 00 00 00 B4 CC 36 A4 12 8B DD 48 97 18 E1 C6 BA A6 65 4C 33 82 41 85 38 79 42 40 83 79 2B 1B 7D 37 65 33 00 00 00 00 FF FF FF 7F FF FF FF 7F 00 01 12 00 00 00 00 00 00 00 00 00 00 00 01 03 00 00 00 00 00 00 00 00

  


00 00 00 00

  


02

  


02 00 00 00


01


32

  


01 00 00 00


00

  


05 00 00 00


04 7C 26 7C 30

  


01 00 00 00


00

  


C2 00 00 00


01


0C 00


31 00


FF FF


0E 00


FF FF FF FF


37 81 F7 00


A1 33 46 01


0D 69 08 00


79 35 07 00


0D 69 08 00


79 35 07 00


0D 69 08 00


79 35 07 00


00 00 00 00


02


02


02


00 00 00 00 00 80 66 40


01


00


00


00 00 00 00 00


A0 86 01 00


04


00


A0 86 01 00


40 0D 03 00


40 0D 03 00


00 00 00 00


40 9C 00 00


00


00 00 01 01 01 01


01


01


01 00 00


00 00 00 00 00 00 00 00


01 00 00 01 00 00 00 40 9C 00 00 00 B4 CC 36 A4 12 8B DD 48 97 18 E1 C6 BA A6 65 4C 33 82 41 85 38 79 42 40 83 79 2B 1B 7D 37 65 33 00 00 00 00 FF FF FF 7F FF FF FF 7F 00 01 12 00 00 00 00 00 00 00 00 00 00 00 01 03 00 00 00 00 00 00 00 00

  


00 00 00 00

  


02

  


02 00 00 00


01


31

  


01 00 00 00


00

  


05 00 00 00


04 7C 26 7C 30

  


01 00 00 00


00

  


C2 00 00 00


01


0C 00


14 00


FF FF


14 00


FF FF FF FF


E8 48 E7 00


D7 2F 3A 01


0D 69 08 00


79 35 07 00


0D 69 08 00


79 35 07 00


0D 69 08 00


79 35 07 00


00 00 00 00


02


02


02


00 00 00 00 00 E0 70 40


01


00


00


00 00 00 00 00


A0 86 01 00


04


00


A0 86 01 00


40 0D 03 00


40 0D 03 00


00 00 00 00


40 9C 00 00


00


00 00 01 01 01 01


01


01


01 00 00


00 00 00 00 00 00 00 00


01 00 00 01 00 00 00 40 9C 00 00 00 B4 CC 36 A4 12 8B DD 48 97 18 E1 C6 BA A6 65 4C 33 82 41 85 38 79 42 40 83 79 2B 1B 7D 37 65 33 00 00 00 00 FF FF FF 7F FF FF FF 7F 00 01 12 00 00 00 00 00 00 00 00 00 00 00 01 03 00 00 00 00 00 00 00 00

  


00 00 00 00

  


02

  


02 00 00 00


01


32

  


01 00 00 00


00

  


05 00 00 00


04 7C 26 7C 30

  


01 00 00 00


00

  


C2 00 00 00


01


0C 00


19 00


FF FF


14 00


FF FF FF FF


B3 78 DC 00


D7 2F 3A 01


0D 69 08 00


79 35 07 00


0D 69 08 00


79 35 07 00


0D 69 08 00


79 35 07 00


00 00 00 00


02


02


02


00 00 00 00 00 E0 70 40


01


00


00


00 00 00 00 00


A0 86 01 00


04


00


A0 86 01 00


40 0D 03 00


40 0D 03 00


00 00 00 00


40 9C 00 00


00


00 00 01 01 01 01


01


01


01 00 00


00 00 00 00 00 00 00 00


01 00 00 01 00 00 00 40 9C 00 00 00 B4 CC 36 A4 12 8B DD 48 97 18 E1 C6 BA A6 65 4C 33 82 41 85 38 79 42 40 83 79 2B 1B 7D 37 65 33 00 00 00 00 FF FF FF 7F FF FF FF 7F 00 01 12 00 00 00 00 00 00 00 00 00 00 00 01 03 00 00 00 00 00 00 00 00

  


00 00 00 00

  


02

  


02 00 00 00


01


32

  


01 00 00 00


00

  


05 00 00 00


04 7C 26 7C 30

  


01 00 00 00


00

  


C2 00 00 00


01


0C 00


19 00


FF FF


12 00


FF FF FF FF


42 7E C5 00


3E B6 9F 01


93 07 18 00


79 35 07 00


93 07 18 00


79 35 07 00


93 07 18 00


79 35 07 00


00 00 00 00


02


02


02


00 00 00 00 00 80 66 40


01


00


00


00 00 00 00 00


A0 86 01 00


04


00


A0 86 01 00


40 0D 03 00


40 0D 03 00


00 00 00 00


40 9C 00 00


00


00 00 01 01 01 01


01


01


01 00 00


00 00 00 00 00 00 00 00


01 00 00 01 00 00 00 40 9C 00 00 00 1D E7 97 64 9F FE 64 49 A1 8D FA 89 45 20 30 0C 33 82 41 85 38 79 42 40 83 79 2B 1B 7D 37 65 33 00 00 00 00 FF FF FF 7F FF FF FF 7F 00 01 12 00 00 00 00 00 00 00 00 00 00 00 01 03 00 00 00 00 00 00 00 00

  


00 00 00 00

  


02

  


02 00 00 00


01


31

  


01 00 00 00


00

  


05 00 00 00


04 7C 26 7C 30

  


01 00 00 00


00

  


C2 00 00 00


01


0C 00


31 00


FF FF


12 00


FF FF FF FF


32 BD EA 00


3E B6 9F 01


93 07 18 00


79 35 07 00


93 07 18 00


79 35 07 00


93 07 18 00


79 35 07 00


00 00 00 00


03


03


03


00 00 00 00 00 80 66 40


01


00


00


00 00 00 00 00


A0 86 01 00


04


00


A0 86 01 00


40 0D 03 00


40 0D 03 00


00 00 00 00


40 9C 00 00


00


00 00 01 01 01 01


01


01


01 00 00


00 00 00 00 00 00 00 00


01 00 00 01 00 00 00 40 9C 00 00 00 36 EF C5 5B CF 96 71 4C BA F1 E0 E6 36 C2 CC 39 33 82 41 85 38 79 42 40 83 79 2B 1B 7D 37 65 33 00 00 00 00 FF FF FF 7F FF FF FF 7F 00 01 12 00 00 00 00 00 00 00 00 00 00 00 01 03 00 00 00 00 00 00 00 00

  


00 00 00 00

  


02

  


02 00 00 00


01


31

  


01 00 00 00


00

  


05 00 00 00


04 7C 26 7C 30

  


01 00 00 00


00

  


C2 00 00 00


01


0C 00


19 00


FF FF


0F 00


FF FF FF FF


9A F1 C5 00


8D F9 58 01


0D 69 08 00


79 35 07 00


0D 69 08 00


79 35 07 00


0D 69 08 00


79 35 07 00


00 00 00 00


02


02


02


00 00 00 00 00 80 56 40


01


00


00


00 00 00 00 00


A0 86 01 00


04


00


A0 86 01 00


40 0D 03 00


40 0D 03 00


00 00 00 00


40 9C 00 00


00


00 00 01 01 01 01


01


01


01 00 00


00 00 00 00 00 00 00 00


01 00 00 01 00 00 00 40 9C 00 00 00 B4 CC 36 A4 12 8B DD 48 97 18 E1 C6 BA A6 65 4C 33 82 41 85 38 79 42 40 83 79 2B 1B 7D 37 65 33 00 00 00 00 FF FF FF 7F FF FF FF 7F 00 01 12 00 00 00 00 00 00 00 00 00 00 00 01 03 00 00 00 00 00 00 00 00

  


00 00 00 00

  


02

  


02 00 00 00


01


32

  


01 00 00 00


00

  


05 00 00 00


04 7C 26 7C 30

  


01 00 00 00


00

  


C2 00 00 00


01


0C 00


10 00


FF FF


0F 00


FF FF FF FF


CF C1 D0 00


8D F9 58 01


0D 69 08 00


79 35 07 00


0D 69 08 00


79 35 07 00


0D 69 08 00


79 35 07 00


00 00 00 00


02


02


02


00 00 00 00 00 80 56 40


01


00


00


00 00 00 00 00


A0 86 01 00


04


00


A0 86 01 00


40 0D 03 00


40 0D 03 00


00 00 00 00


40 9C 00 00


00


00 00 01 01 01 01


01


01


01 00 00


00 00 00 00 00 00 00 00


01 00 00 01 00 00 00 40 9C 00 00 00 B4 CC 36 A4 12 8B DD 48 97 18 E1 C6 BA A6 65 4C 33 82 41 85 38 79 42 40 83 79 2B 1B 7D 37 65 33 00 00 00 00 FF FF FF 7F FF FF FF 7F 00 01 12 00 00 00 00 00 00 00 00 00 00 00 01 03 00 00 00 00 00 00 00 00

  


00 00 00 00

  


02

  


02 00 00 00


01


31

  


01 00 00 00


00

  


05 00 00 00


04 7C 26 7C 30

  


01 00 00 00


00

  


C2 00 00 00


4A


0C 00


19 00


FF FF


00 00


FF FF FF FF


33 E9 D3 00


4E A7 BD 00


CA 03 0C 00


CA 03 0C 00


CA 03 0C 00


CA 03 0C 00


CA 03 0C 00


CA 03 0C 00


79 35 07 00


01


01


01


00 00 00 00 00 80 56 40


01


00


00


00 00 00 00 02


A0 86 01 00


04


00


A0 86 01 00


40 0D 03 00


40 0D 03 00


00 00 00 00


40 9C 00 00


00


00 01 01 01 01 01


01


01


01 01 00


00 00 00 00 00 00 00 00


0F 00 03 01 00 00 00 40 9C 00 00 00 8E 54 57 D5 EF A6 78 4F A4 F4 DB 92 5F FF 07 C3 33 82 41 85 38 79 42 40 83 79 2B 1B 7D 37 65 33 00 00 00 00 FF FF FF 7F FF FF FF 7F 00 01 12 00 00 00 00 00 00 00 00 00 00 00 01 03 00 00 00 00 00 00 00 00

  


00 00 00 00

  


02

  


02 00 00 00


01


32

  


01 00 00 00


00

  


05 00 00 00


04 7C 26 7C 30

  


01 00 00 00


00

  


C2 00 00 00


4A


0C 00


14 00


FF FF


00 00


FF FF FF FF


33 E9 D3 00


AC 7D FC 00


CA 03 0C 00


CA 03 0C 00


CA 03 0C 00


CA 03 0C 00


CA 03 0C 00


CA 03 0C 00


79 35 07 00


02


02


02


00 00 00 00 00 80 56 40


01


00


00


00 00 00 00 02


A0 86 01 00


04


00


A0 86 01 00


40 0D 03 00


40 0D 03 00


00 00 00 00


40 9C 00 00


00


00 01 01 01 01 01


01


01


01 01 00


00 00 00 00 00 00 00 00


0F 00 03 01 00 00 00 40 9C 00 00 00 61 6C F0 3E EC 60 44 41 BD D0 C7 1E B3 95 88 21 33 82 41 85 38 79 42 40 83 79 2B 1B 7D 37 65 33 00 00 00 00 FF FF FF 7F FF FF FF 7F 00 01 12 00 00 00 00 00 00 00 00 00 00 00 01 03 00 00 00 00 00 00 00 00

  


00 00 00 00

  


02

  


02 00 00 00


01


31

  


01 00 00 00


00

  


05 00 00 00


04 7C 26 7C 30

  


01 00 00 00


00

  


C2 00 00 00


4A


0C 00


14 00


FF FF


01 00


FF FF FF FF


15 13 E7 00


66 35 23 01


A1 9C 09 00


A1 9C 09 00


A1 9C 09 00


A1 9C 09 00


A1 9C 09 00


A1 9C 09 00


E5 01 06 00


02


02


02


00 00 00 00 00 80 66 40


01


00


00


00 00 00 00 02


A0 86 01 00


04


00


A0 86 01 00


40 0D 03 00


40 0D 03 00


00 00 00 00


40 9C 00 00


00


00 01 01 01 01 01


01


01


01 01 00


00 00 00 00 00 00 00 00


0F 00 03 01 00 00 00 40 9C 00 00 00 6B 1E CE A4 B2 2F 80 42 82 CB 2D BB AE 73 9E 71 33 82 41 85 38 79 42 40 83 79 2B 1B 7D 37 65 33 00 00 00 00 FF FF FF 7F FF FF FF 7F 00 01 12 00 00 00 00 00 00 00 00 00 00 00 01 03 00 00 00 00 00 00 00 00

  


00 00 00 00

  


02

  


02 00 00 00


01


32

  


01 00 00 00


00

  


05 00 00 00


04 7C 26 7C 30

  


01 00 00 00


00

  


C2 00 00 00


4A


0C 00


19 00


FF FF


01 00


FF FF FF FF


D5 D0 D7 00


66 35 23 01


A1 9C 09 00


A1 9C 09 00


A1 9C 09 00


A1 9C 09 00


A1 9C 09 00


A1 9C 09 00


E5 01 06 00


01


01


01


00 00 00 00 00 80 66 40


01


00


00


00 00 00 00 02


A0 86 01 00


04


00


A0 86 01 00


40 0D 03 00


40 0D 03 00


00 00 00 00


40 9C 00 00


00


00 01 01 01 01 01


01


01


01 01 00


00 00 00 00 00 00 00 00


0F 00 03 01 00 00 00 40 9C 00 00 00 38 C4 88 68 59 11 50 45 94 93 CC 0C 22 F1 0F F7 33 82 41 85 38 79 42 40 83 79 2B 1B 7D 37 65 33 00 00 00 00 FF FF FF 7F FF FF FF 7F 00 01 12 00 00 00 00 00 00 00 00 00 00 00 01 03 00 00 00 00 00 00 00 00

  


00 00 00 00

  


02

  


02 00 00 00


01


31

  


01 00 00 00


00

  


05 00 00 00


04 7C 26 7C 30

  


01 00 00 00


00

  


C2 00 00 00


4A


0C 00


0E 00


FF FF


08 00


FF FF FF FF


FE 1F 1C 02


07 8E C2 01


BC 04 0F 00


BC 04 0F 00


BC 04 0F 00


BC 04 0F 00


BC 04 0F 00


BC 04 0F 00


D7 02 09 00


01


01


01


00 00 00 00 00 00 00 00


01


00


00


00 00 00 00 02


A0 86 01 00


04


00


A0 86 01 00


40 0D 03 00


40 0D 03 00


00 00 00 00


40 9C 00 00


00


00 01 01 01 01 01


01


01


01 01 00


00 00 00 00 00 00 00 00


0F 00 03 01 00 00 00 40 9C 00 00 00 44 A5 0C 20 F2 A6 C2 47 87 73 6D 35 80 A5 A2 9E 33 82 41 85 38 79 42 40 83 79 2B 1B 7D 37 65 33 00 00 00 00 FF FF FF 7F FF FF FF 7F 00 01 12 00 00 00 00 00 00 00 00 00 00 00 01 03 00 00 00 00 00 00 00 00

  


00 00 00 00

  


02

  


02 00 00 00


01


30

  


01 00 00 00


00

  


05 00 00 00


04 7C 26 7C 30

  


01 00 00 00


00

  


C2 00 00 00


4A


0C 00


19 00


FF FF


08 00


FF FF FF FF


5D CE 0C 02


66 3C B3 01


CA 03 0C 00


CA 03 0C 00


CA 03 0C 00


CA 03 0C 00


CA 03 0C 00


CA 03 0C 00


D7 02 09 00


02


02


02


00 00 00 00 00 00 00 00


01


00


00


00 00 00 00 02


A0 86 01 00


04


00


A0 86 01 00


40 0D 03 00


40 0D 03 00


00 00 00 00


40 9C 00 00


00


00 01 01 01 01 01


01


01


01 01 00


00 00 00 00 02 00 00 00


0F 00 03 01 00 00 00 40 9C 00 00 00 6E FD 22 25 EB 00 B2 40 81 A9 F9 8F 47 DC C3 F0 33 82 41 85 38 79 42 40 83 79 2B 1B 7D 37 65 33 00 00 00 00 FF FF FF 7F FF FF FF 7F 00 01 12 00 00 00 00 00 00 00 00 00 00 00 01 03 00 00 00 00 00 00 00 00

  


00 00 00 00

  


02

  


02 00 00 00


01


30

  


01 00 00 00


00

  


05 00 00 00


04 7C 26 7C 30

  


01 00 00 00


00

  


C2 00 00 00


4A


0C 00


19 00


FF FF


08 00


FF FF FF FF


9F 71 2B 02


66 3C B3 01


CA 03 0C 00


CA 03 0C 00


CA 03 0C 00


CA 03 0C 00


CA 03 0C 00


CA 03 0C 00


D7 02 09 00


02


02


02


00 00 00 00 00 00 00 00


01


00


00


00 00 00 00 02


A0 86 01 00


04


00


A0 86 01 00


40 0D 03 00


40 0D 03 00


00 00 00 00


40 9C 00 00


00


00 01 01 01 01 01


01


01


01 01 00


00 00 00 00 02 00 00 00


0F 00 03 01 00 00 00 40 9C 00 00 00 6E FD 22 25 EB 00 B2 40 81 A9 F9 8F 47 DC C3 F0 33 82 41 85 38 79 42 40 83 79 2B 1B 7D 37 65 33 00 00 00 00 FF FF FF 7F FF FF FF 7F 00 01 12 00 00 00 00 00 00 00 00 00 00 00 01 03 00 00 00 00 00 00 00 00

  


00 00 00 00

  


02

  


02 00 00 00


01


30

  


01 00 00 00


00

  


05 00 00 00


04 7C 26 7C 30

  


01 00 00 00


00

  


C2 00 00 00


4A


0C 00


19 00


FF FF


08 00


FF FF FF FF


9F 71 2B 02


A8 DF D1 01


CA 03 0C 00


CA 03 0C 00


CA 03 0C 00


CA 03 0C 00


CA 03 0C 00


CA 03 0C 00


D7 02 09 00


02


02


02


00 00 00 00 00 00 00 00


01


00


00


00 00 00 00 02


A0 86 01 00


04


00


A0 86 01 00


40 0D 03 00


40 0D 03 00


00 00 00 00


40 9C 00 00


00


00 01 01 01 01 01


01


01


01 01 00


00 00 00 00 02 00 00 00


0F 00 03 01 00 00 00 40 9C 00 00 00 6E FD 22 25 EB 00 B2 40 81 A9 F9 8F 47 DC C3 F0 33 82 41 85 38 79 42 40 83 79 2B 1B 7D 37 65 33 00 00 00 00 FF FF FF 7F FF FF FF 7F 00 01 12 00 00 00 00 00 00 00 00 00 00 00 01 03 00 00 00 00 00 00 00 00

  


00 00 00 00

  


02

  


02 00 00 00


01


30

  


01 00 00 00


00

  


05 00 00 00


04 7C 26 7C 30

  


01 00 00 00


00

  


C2 00 00 00


4A


0C 00


19 00


FF FF


08 00


FF FF FF FF


5D CE 0C 02


A8 DF D1 01


CA 03 0C 00


CA 03 0C 00


CA 03 0C 00


CA 03 0C 00


CA 03 0C 00


CA 03 0C 00


D7 02 09 00


02


02


02


00 00 00 00 00 00 00 00


01


00


00


00 00 00 00 02


A0 86 01 00


04


00


A0 86 01 00


40 0D 03 00


40 0D 03 00


00 00 00 00


40 9C 00 00


00


00 01 01 01 01 01


01


01


01 01 00


00 00 00 00 02 00 00 00


0F 00 03 01 00 00 00 40 9C 00 00 00 6E FD 22 25 EB 00 B2 40 81 A9 F9 8F 47 DC C3 F0 33 82 41 85 38 79 42 40 83 79 2B 1B 7D 37 65 33 00 00 00 00 FF FF FF 7F FF FF FF 7F 00 01 12 00 00 00 00 00 00 00 00 00 00 00 01 03 00 00 00 00 00 00 00 00

  


00 00 00 00

  


02

  


03 00 00 00


02


32 33

  


01 00 00 00


00

  


05 00 00 00


04 7C 26 7C 30

  


01 00 00 00


00

  


C2 00 00 00


4A


0C 00


17 00


FF FF


04 00


FF FF FF FF


D1 E0 22 01


64 43 02 02


60 AE 0A 00


60 AE 0A 00


60 AE 0A 00


60 AE 0A 00


60 AE 0A 00


60 AE 0A 00


E5 01 06 00


01


01


01


00 00 00 00 00 80 66 40


01


00


00


00 00 00 00 02


A0 86 01 00


04


00


A0 86 01 00


40 0D 03 00


40 0D 03 00


00 00 00 00


40 9C 00 00


00


00 01 01 01 01 01


01


01


01 01 00


00 00 00 00 00 00 00 00


0F 00 03 01 00 00 00 40 9C 00 00 00 FD 59 30 C0 71 4B E9 44 89 E9 20 D2 B2 A8 B7 97 33 82 41 85 38 79 42 40 83 79 2B 1B 7D 37 65 33 00 00 00 00 FF FF FF 7F FF FF FF 7F 00 01 12 00 00 00 00 00 00 00 00 00 00 00 01 03 00 00 00 00 00 00 00 00

  


00 00 00 00

  


02

  


03 00 00 00


02


32 32

  


01 00 00 00


00

  


05 00 00 00


04 7C 26 7C 30

  


01 00 00 00


00

  


C2 00 00 00


4A


0C 00


08 00


FF FF


04 00


FF FF FF FF


D1 E0 22 01


24 01 F3 01


60 AE 0A 00


60 AE 0A 00


60 AE 0A 00


60 AE 0A 00


60 AE 0A 00


60 AE 0A 00


E5 01 06 00


01


01


01


00 00 00 00 00 80 66 40


01


00


00


00 00 00 00 02


A0 86 01 00


04


00


A0 86 01 00


40 0D 03 00


40 0D 03 00


00 00 00 00


40 9C 00 00


00


00 01 01 01 01 01


01


01


01 01 00


00 00 00 00 00 00 00 00


0F 00 03 01 00 00 00 40 9C 00 00 00 FD 59 30 C0 71 4B E9 44 89 E9 20 D2 B2 A8 B7 97 33 82 41 85 38 79 42 40 83 79 2B 1B 7D 37 65 33 00 00 00 00 FF FF FF 7F FF FF FF 7F 00 01 12 00 00 00 00 00 00 00 00 00 00 00 01 03 00 00 00 00 00 00 00 00

  


00 00 00 00

  


02

  


03 00 00 00


02


32 31

  


01 00 00 00


00

  


05 00 00 00


04 7C 26 7C 30

  


01 00 00 00


00

  


C2 00 00 00


4A


0C 00


04 00


FF FF


04 00


FF FF FF FF


D1 E0 22 01


E4 BE E3 01


60 AE 0A 00


60 AE 0A 00


60 AE 0A 00


60 AE 0A 00


60 AE 0A 00


60 AE 0A 00


E5 01 06 00


01


01


01


00 00 00 00 00 80 66 40


01


00


00


00 00 00 00 02


A0 86 01 00


04


00


A0 86 01 00


40 0D 03 00


40 0D 03 00


00 00 00 00


40 9C 00 00


00


00 01 01 01 01 01


01


01


01 01 00


00 00 00 00 00 00 00 00


0F 00 03 01 00 00 00 40 9C 00 00 00 FD 59 30 C0 71 4B E9 44 89 E9 20 D2 B2 A8 B7 97 33 82 41 85 38 79 42 40 83 79 2B 1B 7D 37 65 33 00 00 00 00 FF FF FF 7F FF FF FF 7F 00 01 12 00 00 00 00 00 00 00 00 00 00 00 01 03 00 00 00 00 00 00 00 00

  


00 00 00 00

  


02

  


03 00 00 00


02


32 30

  


01 00 00 00


00

  


05 00 00 00


04 7C 26 7C 30

  


01 00 00 00


00

  


C2 00 00 00


4A


0C 00


1B 00


FF FF


04 00


FF FF FF FF


D1 E0 22 01


A4 7C D4 01


60 AE 0A 00


60 AE 0A 00


60 AE 0A 00


60 AE 0A 00


60 AE 0A 00


60 AE 0A 00


E5 01 06 00


01


01


01


00 00 00 00 00 80 66 40


01


00


00


00 00 00 00 02


A0 86 01 00


04


00


A0 86 01 00


40 0D 03 00


40 0D 03 00


00 00 00 00


40 9C 00 00


00


00 01 01 01 01 01


01


01


01 01 00


00 00 00 00 00 00 00 00


0F 00 03 01 00 00 00 40 9C 00 00 00 FD 59 30 C0 71 4B E9 44 89 E9 20 D2 B2 A8 B7 97 33 82 41 85 38 79 42 40 83 79 2B 1B 7D 37 65 33 00 00 00 00 FF FF FF 7F FF FF FF 7F 00 01 12 00 00 00 00 00 00 00 00 00 00 00 01 03 00 00 00 00 00 00 00 00

  


00 00 00 00

  


02

  


03 00 00 00


02


31 39

  


01 00 00 00


00

  


05 00 00 00


04 7C 26 7C 30

  


01 00 00 00


00

  


C2 00 00 00


4A


0C 00


1B 00


FF FF


04 00


FF FF FF FF


D1 E0 22 01


64 3A C5 01


60 AE 0A 00


60 AE 0A 00


60 AE 0A 00


60 AE 0A 00


60 AE 0A 00


60 AE 0A 00


E5 01 06 00


01


01


01


00 00 00 00 00 80 66 40


01


00


00


00 00 00 00 02


A0 86 01 00


04


00


A0 86 01 00


40 0D 03 00


40 0D 03 00


00 00 00 00


40 9C 00 00


00


00 01 01 01 01 01


01


01


01 01 00


00 00 00 00 00 00 00 00


0F 00 03 01 00 00 00 40 9C 00 00 00 FD 59 30 C0 71 4B E9 44 89 E9 20 D2 B2 A8 B7 97 33 82 41 85 38 79 42 40 83 79 2B 1B 7D 37 65 33 00 00 00 00 FF FF FF 7F FF FF FF 7F 00 01 12 00 00 00 00 00 00 00 00 00 00 00 01 03 00 00 00 00 00 00 00 00

  


00 00 00 00

  


02

  


03 00 00 00


02


31 38

  


01 00 00 00


00

  


05 00 00 00


04 7C 26 7C 30

  


01 00 00 00


00

  


C2 00 00 00


4A


0C 00


22 00


FF FF


04 00


FF FF FF FF


D1 E0 22 01


24 F8 B5 01


60 AE 0A 00


60 AE 0A 00


60 AE 0A 00


60 AE 0A 00


60 AE 0A 00


60 AE 0A 00


E5 01 06 00


01


01


01


00 00 00 00 00 80 66 40


01


00


00


00 00 00 00 02


A0 86 01 00


04


00


A0 86 01 00


40 0D 03 00


40 0D 03 00


00 00 00 00


40 9C 00 00


00


00 01 01 01 01 01


01


01


01 01 00


00 00 00 00 00 00 00 00


0F 00 03 01 00 00 00 40 9C 00 00 00 FD 59 30 C0 71 4B E9 44 89 E9 20 D2 B2 A8 B7 97 33 82 41 85 38 79 42 40 83 79 2B 1B 7D 37 65 33 00 00 00 00 FF FF FF 7F FF FF FF 7F 00 01 12 00 00 00 00 00 00 00 00 00 00 00 01 03 00 00 00 00 00 00 00 00

  


00 00 00 00

  


02

  


03 00 00 00


02


31 37

  


01 00 00 00


00

  


05 00 00 00


04 7C 26 7C 30

  


01 00 00 00


00

  


C2 00 00 00


4A


0C 00


21 00


FF FF


04 00


FF FF FF FF


D1 E0 22 01


E4 B5 A6 01


60 AE 0A 00


60 AE 0A 00


60 AE 0A 00


60 AE 0A 00


60 AE 0A 00


60 AE 0A 00


E5 01 06 00


01


01


01


00 00 00 00 00 80 66 40


01


00


00


00 00 00 00 02


A0 86 01 00


04


00


A0 86 01 00


40 0D 03 00


40 0D 03 00


00 00 00 00


40 9C 00 00


00


00 01 01 01 01 01


01


01


01 01 00


00 00 00 00 00 00 00 00


0F 00 03 01 00 00 00 40 9C 00 00 00 FD 59 30 C0 71 4B E9 44 89 E9 20 D2 B2 A8 B7 97 33 82 41 85 38 79 42 40 83 79 2B 1B 7D 37 65 33 00 00 00 00 FF FF FF 7F FF FF FF 7F 00 01 12 00 00 00 00 00 00 00 00 00 00 00 01 03 00 00 00 00 00 00 00 00

  


00 00 00 00

  


02

  


03 00 00 00


02


31 36

  


01 00 00 00


00

  


05 00 00 00


04 7C 26 7C 30

  


01 00 00 00


00

  


C2 00 00 00


4A


0C 00


20 00


FF FF


04 00


FF FF FF FF


D1 E0 22 01


A4 73 97 01


60 AE 0A 00


60 AE 0A 00


60 AE 0A 00


60 AE 0A 00


60 AE 0A 00


60 AE 0A 00


E5 01 06 00


01


01


01


00 00 00 00 00 80 66 40


01


00


00


00 00 00 00 02


A0 86 01 00


04


00


A0 86 01 00


40 0D 03 00


40 0D 03 00


00 00 00 00


40 9C 00 00


00


00 01 01 01 01 01


01


01


01 01 00


00 00 00 00 00 00 00 00


0F 00 03 01 00 00 00 40 9C 00 00 00 FD 59 30 C0 71 4B E9 44 89 E9 20 D2 B2 A8 B7 97 33 82 41 85 38 79 42 40 83 79 2B 1B 7D 37 65 33 00 00 00 00 FF FF FF 7F FF FF FF 7F 00 01 12 00 00 00 00 00 00 00 00 00 00 00 01 03 00 00 00 00 00 00 00 00

  


00 00 00 00

  


02

  


03 00 00 00


02


31 35

  


01 00 00 00


00

  


05 00 00 00


04 7C 26 7C 30

  


01 00 00 00


00

  


C2 00 00 00


4A


0C 00


1F 00


FF FF


04 00


FF FF FF FF


D1 E0 22 01


64 31 88 01


60 AE 0A 00


60 AE 0A 00


60 AE 0A 00


60 AE 0A 00


60 AE 0A 00


60 AE 0A 00


E5 01 06 00


01


01


01


00 00 00 00 00 80 66 40


01


00


00


00 00 00 00 02


A0 86 01 00


04


00


A0 86 01 00


40 0D 03 00


40 0D 03 00


00 00 00 00


40 9C 00 00


00


00 01 01 01 01 01


01


01


01 01 00


00 00 00 00 00 00 00 00


0F 00 03 01 00 00 00 40 9C 00 00 00 FD 59 30 C0 71 4B E9 44 89 E9 20 D2 B2 A8 B7 97 33 82 41 85 38 79 42 40 83 79 2B 1B 7D 37 65 33 00 00 00 00 FF FF FF 7F FF FF FF 7F 00 01 12 00 00 00 00 00 00 00 00 00 00 00 01 03 00 00 00 00 00 00 00 00

  


00 00 00 00

  


02

  


03 00 00 00


02


31 34

  


01 00 00 00


00

  


05 00 00 00


04 7C 26 7C 30

  


01 00 00 00


00

  


C2 00 00 00


4A


0C 00


19 00


FF FF


04 00


FF FF FF FF


D1 E0 22 01


24 EF 78 01


60 AE 0A 00


60 AE 0A 00


60 AE 0A 00


60 AE 0A 00


60 AE 0A 00


60 AE 0A 00


E5 01 06 00


01


01


01


00 00 00 00 00 80 66 40


01


00


00


00 00 00 00 02


A0 86 01 00


04


00


A0 86 01 00


40 0D 03 00


40 0D 03 00


00 00 00 00


40 9C 00 00


00


00 01 01 01 01 01


01


01


01 01 00


00 00 00 00 00 00 00 00


0F 00 03 01 00 00 00 40 9C 00 00 00 FD 59 30 C0 71 4B E9 44 89 E9 20 D2 B2 A8 B7 97 33 82 41 85 38 79 42 40 83 79 2B 1B 7D 37 65 33 00 00 00 00 FF FF FF 7F FF FF FF 7F 00 01 12 00 00 00 00 00 00 00 00 00 00 00 01 03 00 00 00 00 00 00 00 00

  


00 00 00 00

  


02

  


03 00 00 00


02


31 33

  


01 00 00 00


00

  


05 00 00 00


04 7C 26 7C 30

  


01 00 00 00


00

  


C2 00 00 00


4A


0C 00


33 00


FF FF


04 00


FF FF FF FF


D1 E0 22 01


E4 AC 69 01


60 AE 0A 00


60 AE 0A 00


60 AE 0A 00


60 AE 0A 00


60 AE 0A 00


60 AE 0A 00


E5 01 06 00


01


01


01


00 00 00 00 00 80 66 40


01


00


00


00 00 00 00 02


A0 86 01 00


04


00


A0 86 01 00


40 0D 03 00


40 0D 03 00


00 00 00 00


40 9C 00 00


00


00 01 01 01 01 01


01


01


01 01 00


00 00 00 00 00 00 00 00


0F 00 03 01 00 00 00 40 9C 00 00 00 FD 59 30 C0 71 4B E9 44 89 E9 20 D2 B2 A8 B7 97 33 82 41 85 38 79 42 40 83 79 2B 1B 7D 37 65 33 00 00 00 00 FF FF FF 7F FF FF FF 7F 00 01 12 00 00 00 00 00 00 00 00 00 00 00 01 03 00 00 00 00 00 00 00 00

  


00 00 00 00

  


02

  


03 00 00 00


02


31 32

  


01 00 00 00


00

  


05 00 00 00


04 7C 26 7C 30

  


01 00 00 00


00

  


C2 00 00 00


4A


0C 00


30 00


FF FF


04 00


FF FF FF FF


D1 E0 22 01


A4 6A 5A 01


60 AE 0A 00


60 AE 0A 00


60 AE 0A 00


60 AE 0A 00


60 AE 0A 00


60 AE 0A 00


E5 01 06 00


01


01


01


00 00 00 00 00 80 66 40


01


00


00


00 00 00 00 02


A0 86 01 00


04


00


A0 86 01 00


40 0D 03 00


40 0D 03 00


00 00 00 00


40 9C 00 00


00


00 01 01 01 01 01


01


01


01 01 00


00 00 00 00 00 00 00 00


0F 00 03 01 00 00 00 40 9C 00 00 00 FD 59 30 C0 71 4B E9 44 89 E9 20 D2 B2 A8 B7 97 33 82 41 85 38 79 42 40 83 79 2B 1B 7D 37 65 33 00 00 00 00 FF FF FF 7F FF FF FF 7F 00 01 12 00 00 00 00 00 00 00 00 00 00 00 01 03 00 00 00 00 00 00 00 00

  


00 00 00 00

  


02

  


03 00 00 00


02


31 31

  


01 00 00 00


00

  


05 00 00 00


04 7C 26 7C 30

  


01 00 00 00


00

  


C2 00 00 00


4A


0C 00


2F 00


FF FF


04 00


FF FF FF FF


D1 E0 22 01


64 28 4B 01


60 AE 0A 00


60 AE 0A 00


60 AE 0A 00


60 AE 0A 00


60 AE 0A 00


60 AE 0A 00


E5 01 06 00


01


01


01


00 00 00 00 00 80 66 40


01


00


00


00 00 00 00 02


A0 86 01 00


04


00


A0 86 01 00


40 0D 03 00


40 0D 03 00


00 00 00 00


40 9C 00 00


00


00 01 01 01 01 01


01


01


01 01 00


00 00 00 00 00 00 00 00


0F 00 03 01 00 00 00 40 9C 00 00 00 FD 59 30 C0 71 4B E9 44 89 E9 20 D2 B2 A8 B7 97 33 82 41 85 38 79 42 40 83 79 2B 1B 7D 37 65 33 00 00 00 00 FF FF FF 7F FF FF FF 7F 00 01 12 00 00 00 00 00 00 00 00 00 00 00 01 03 00 00 00 00 00 00 00 00

  


00 00 00 00

  


02

  


03 00 00 00


02


31 30

  


01 00 00 00


00

  


05 00 00 00


04 7C 26 7C 30

  


01 00 00 00


00

  


C2 00 00 00


4A


0C 00


2E 00


FF FF


04 00


FF FF FF FF


D1 E0 22 01


24 E6 3B 01


60 AE 0A 00


60 AE 0A 00


60 AE 0A 00


60 AE 0A 00


60 AE 0A 00


60 AE 0A 00


E5 01 06 00


01


01


01


00 00 00 00 00 80 66 40


01


00


00


00 00 00 00 02


A0 86 01 00


04


00


A0 86 01 00


40 0D 03 00


40 0D 03 00


00 00 00 00


40 9C 00 00


00


00 01 01 01 01 01


01


01


01 01 00


00 00 00 00 00 00 00 00


0F 00 03 01 00 00 00 40 9C 00 00 00 FD 59 30 C0 71 4B E9 44 89 E9 20 D2 B2 A8 B7 97 33 82 41 85 38 79 42 40 83 79 2B 1B 7D 37 65 33 00 00 00 00 FF FF FF 7F FF FF FF 7F 00 01 12 00 00 00 00 00 00 00 00 00 00 00 01 03 00 00 00 00 00 00 00 00

  


00 00 00 00

  


02

  


02 00 00 00


01


39

  


01 00 00 00


00

  


05 00 00 00


04 7C 26 7C 30

  


01 00 00 00


00

  


C2 00 00 00


4A


0C 00


2D 00


FF FF


04 00


FF FF FF FF


D1 E0 22 01


E4 A3 2C 01


60 AE 0A 00


60 AE 0A 00


60 AE 0A 00


60 AE 0A 00


60 AE 0A 00


60 AE 0A 00


E5 01 06 00


01


01


01


00 00 00 00 00 80 66 40


01


00


00


00 00 00 00 02


A0 86 01 00


04


00


A0 86 01 00


40 0D 03 00


40 0D 03 00


00 00 00 00


40 9C 00 00


00


00 01 01 01 01 01


01


01


01 01 00


00 00 00 00 00 00 00 00


0F 00 03 01 00 00 00 40 9C 00 00 00 FD 59 30 C0 71 4B E9 44 89 E9 20 D2 B2 A8 B7 97 33 82 41 85 38 79 42 40 83 79 2B 1B 7D 37 65 33 00 00 00 00 FF FF FF 7F FF FF FF 7F 00 01 12 00 00 00 00 00 00 00 00 00 00 00 01 03 00 00 00 00 00 00 00 00

  


00 00 00 00

  


02

  


02 00 00 00


01


38

  


01 00 00 00


00

  


05 00 00 00


04 7C 26 7C 30

  


01 00 00 00


00

  


C2 00 00 00


4A


0C 00


2C 00


FF FF


04 00


FF FF FF FF


D1 E0 22 01


A4 61 1D 01


60 AE 0A 00


60 AE 0A 00


60 AE 0A 00


60 AE 0A 00


60 AE 0A 00


60 AE 0A 00


E5 01 06 00


01


01


01


00 00 00 00 00 80 66 40


01


00


00


00 00 00 00 02


A0 86 01 00


04


00


A0 86 01 00


40 0D 03 00


40 0D 03 00


00 00 00 00


40 9C 00 00


00


00 01 01 01 01 01


01


01


01 01 00


00 00 00 00 00 00 00 00


0F 00 03 01 00 00 00 40 9C 00 00 00 FD 59 30 C0 71 4B E9 44 89 E9 20 D2 B2 A8 B7 97 33 82 41 85 38 79 42 40 83 79 2B 1B 7D 37 65 33 00 00 00 00 FF FF FF 7F FF FF FF 7F 00 01 12 00 00 00 00 00 00 00 00 00 00 00 01 03 00 00 00 00 00 00 00 00

  


00 00 00 00

  


02

  


02 00 00 00


01


37

  


01 00 00 00


00

  


05 00 00 00


04 7C 26 7C 30

  


01 00 00 00


00

  


C2 00 00 00


4A


0C 00


2B 00


FF FF


04 00


FF FF FF FF


D1 E0 22 01


64 1F 0E 01


60 AE 0A 00


60 AE 0A 00


60 AE 0A 00


60 AE 0A 00


60 AE 0A 00


60 AE 0A 00


E5 01 06 00


01


01


01


00 00 00 00 00 80 66 40


01


00


00


00 00 00 00 02


A0 86 01 00


04


00


A0 86 01 00


40 0D 03 00


40 0D 03 00


00 00 00 00


40 9C 00 00


00


00 01 01 01 01 01


01


01


01 01 00


00 00 00 00 00 00 00 00


0F 00 03 01 00 00 00 40 9C 00 00 00 FD 59 30 C0 71 4B E9 44 89 E9 20 D2 B2 A8 B7 97 33 82 41 85 38 79 42 40 83 79 2B 1B 7D 37 65 33 00 00 00 00 FF FF FF 7F FF FF FF 7F 00 01 12 00 00 00 00 00 00 00 00 00 00 00 01 03 00 00 00 00 00 00 00 00

  


00 00 00 00

  


02

  


02 00 00 00


01


36

  


01 00 00 00


00

  


05 00 00 00


04 7C 26 7C 30

  


01 00 00 00


00

  


C2 00 00 00


4A


0C 00


2A 00


FF FF


04 00


FF FF FF FF


D1 E0 22 01


24 DD FE 00


60 AE 0A 00


60 AE 0A 00


60 AE 0A 00


60 AE 0A 00


60 AE 0A 00


60 AE 0A 00


E5 01 06 00


01


01


01


00 00 00 00 00 80 66 40


01


00


00


00 00 00 00 02


A0 86 01 00


04


00


A0 86 01 00


40 0D 03 00


40 0D 03 00


00 00 00 00


40 9C 00 00


00


00 01 01 01 01 01


01


01


01 01 00


00 00 00 00 00 00 00 00


0F 00 03 01 00 00 00 40 9C 00 00 00 FD 59 30 C0 71 4B E9 44 89 E9 20 D2 B2 A8 B7 97 33 82 41 85 38 79 42 40 83 79 2B 1B 7D 37 65 33 00 00 00 00 FF FF FF 7F FF FF FF 7F 00 01 12 00 00 00 00 00 00 00 00 00 00 00 01 03 00 00 00 00 00 00 00 00

  


00 00 00 00

  


02

  


02 00 00 00


01


35

  


01 00 00 00


00

  


05 00 00 00


04 7C 26 7C 30

  


01 00 00 00


00

  


C2 00 00 00


4A


0C 00


29 00


FF FF


04 00


FF FF FF FF


D1 E0 22 01


E4 9A EF 00


60 AE 0A 00


60 AE 0A 00


60 AE 0A 00


60 AE 0A 00


60 AE 0A 00


60 AE 0A 00


E5 01 06 00


01


01


01


00 00 00 00 00 80 66 40


01


00


00


00 00 00 00 02


A0 86 01 00


04


00


A0 86 01 00


40 0D 03 00


40 0D 03 00


00 00 00 00


40 9C 00 00


00


00 01 01 01 01 01


01


01


01 01 00


00 00 00 00 00 00 00 00


0F 00 03 01 00 00 00 40 9C 00 00 00 FD 59 30 C0 71 4B E9 44 89 E9 20 D2 B2 A8 B7 97 33 82 41 85 38 79 42 40 83 79 2B 1B 7D 37 65 33 00 00 00 00 FF FF FF 7F FF FF FF 7F 00 01 12 00 00 00 00 00 00 00 00 00 00 00 01 03 00 00 00 00 00 00 00 00

  


00 00 00 00

  


02

  


02 00 00 00


01


34

  


01 00 00 00


00

  


05 00 00 00


04 7C 26 7C 30

  


01 00 00 00


00

  


C2 00 00 00


4A


0C 00


28 00


FF FF


04 00


FF FF FF FF


D1 E0 22 01


A4 58 E0 00


60 AE 0A 00


60 AE 0A 00


60 AE 0A 00


60 AE 0A 00


60 AE 0A 00


60 AE 0A 00


E5 01 06 00


01


01


01


00 00 00 00 00 80 66 40


01


00


00


00 00 00 00 02


A0 86 01 00


04


00


A0 86 01 00


40 0D 03 00


40 0D 03 00


00 00 00 00


40 9C 00 00


00


00 01 01 01 01 01


01


01


01 01 00


00 00 00 00 00 00 00 00


0F 00 03 01 00 00 00 40 9C 00 00 00 FD 59 30 C0 71 4B E9 44 89 E9 20 D2 B2 A8 B7 97 33 82 41 85 38 79 42 40 83 79 2B 1B 7D 37 65 33 00 00 00 00 FF FF FF 7F FF FF FF 7F 00 01 12 00 00 00 00 00 00 00 00 00 00 00 01 03 00 00 00 00 00 00 00 00

  


00 00 00 00

  


02

  


02 00 00 00


01


33

  


01 00 00 00


00

  


05 00 00 00


04 7C 26 7C 30

  


01 00 00 00


00

  


C2 00 00 00


4A


0C 00


27 00


FF FF


04 00


FF FF FF FF


D1 E0 22 01


64 16 D1 00


60 AE 0A 00


60 AE 0A 00


60 AE 0A 00


60 AE 0A 00


60 AE 0A 00


60 AE 0A 00


E5 01 06 00


01


01


01


00 00 00 00 00 80 66 40


01


00


00


00 00 00 00 02


A0 86 01 00


04


00


A0 86 01 00


40 0D 03 00


40 0D 03 00


00 00 00 00


40 9C 00 00


00


00 01 01 01 01 01


01


01


01 01 00


00 00 00 00 00 00 00 00


0F 00 03 01 00 00 00 40 9C 00 00 00 FD 59 30 C0 71 4B E9 44 89 E9 20 D2 B2 A8 B7 97 33 82 41 85 38 79 42 40 83 79 2B 1B 7D 37 65 33 00 00 00 00 FF FF FF 7F FF FF FF 7F 00 01 12 00 00 00 00 00 00 00 00 00 00 00 01 03 00 00 00 00 00 00 00 00

  


00 00 00 00

  


02

  


02 00 00 00


01


32

  


01 00 00 00


00

  


05 00 00 00


04 7C 26 7C 30

  


01 00 00 00


00

  


C2 00 00 00


4A


0C 00


00 00


FF FF


04 00


FF FF FF FF


D1 E0 22 01


24 D4 C1 00


60 AE 0A 00


60 AE 0A 00


60 AE 0A 00


60 AE 0A 00


60 AE 0A 00


60 AE 0A 00


E5 01 06 00


01


01


01


00 00 00 00 00 80 66 40


01


00


00


00 00 00 00 02


A0 86 01 00


04


00


A0 86 01 00


40 0D 03 00


40 0D 03 00


00 00 00 00


40 9C 00 00


00


00 01 01 01 01 01


01


01


01 01 00


00 00 00 00 00 00 00 00


0F 00 03 01 00 00 00 40 9C 00 00 00 FD 59 30 C0 71 4B E9 44 89 E9 20 D2 B2 A8 B7 97 33 82 41 85 38 79 42 40 83 79 2B 1B 7D 37 65 33 00 00 00 00 FF FF FF 7F FF FF FF 7F 00 01 12 00 00 00 00 00 00 00 00 00 00 00 01 03 00 00 00 00 00 00 00 00

  


00 00 00 00

  


02

  


02 00 00 00


01


31

  


01 00 00 00


00

  


05 00 00 00


04 7C 26 7C 30

  


01 00 00 00


00

  


C2 00 00 00


4A


0C 00


19 00


FF FF


04 00


FF FF FF FF


D1 E0 22 01


E4 91 B2 00


60 AE 0A 00


60 AE 0A 00


60 AE 0A 00


60 AE 0A 00


60 AE 0A 00


60 AE 0A 00


E5 01 06 00


01


01


01


00 00 00 00 00 80 66 40


01


00


00


00 00 00 00 02


A0 86 01 00


04


00


A0 86 01 00


40 0D 03 00


40 0D 03 00


00 00 00 00


40 9C 00 00


00


00 01 01 01 01 01


01


01


01 01 00


00 00 00 00 00 00 00 00


0F 00 03 01 00 00 00 40 9C 00 00 00 FD 59 30 C0 71 4B E9 44 89 E9 20 D2 B2 A8 B7 97 33 82 41 85 38 79 42 40 83 79 2B 1B 7D 37 65 33 00 00 00 00 FF FF FF 7F FF FF FF 7F 00 01 12 00 00 00 00 00 00 00 00 00 00 00 01 03 00 00 00 00 00 00 00 00

  


00 00 00 00

  


02

  


02 00 00 00


01


30

  


01 00 00 00


00

  


05 00 00 00


04 7C 26 7C 30

  


01 00 00 00


00

  


C2 00 00 00


4A


0C 00


11 00


FF FF


04 00


FF FF FF FF


D1 E0 22 01


A4 4F A3 00


60 AE 0A 00


60 AE 0A 00


60 AE 0A 00


60 AE 0A 00


60 AE 0A 00


60 AE 0A 00


E5 01 06 00


01


01


01


00 00 00 00 00 80 66 40


01


00


00


00 00 00 00 02


A0 86 01 00


04


00


A0 86 01 00


40 0D 03 00


40 0D 03 00


00 00 00 00


40 9C 00 00


00


00 01 01 01 01 01


01


01


01 01 00


00 00 00 00 00 00 00 00


0F 00 03 01 00 00 00 40 9C 00 00 00 FD 59 30 C0 71 4B E9 44 89 E9 20 D2 B2 A8 B7 97 33 82 41 85 38 79 42 40 83 79 2B 1B 7D 37 65 33 00 00 00 00 FF FF FF 7F FF FF FF 7F 00 01 12 00 00 00 00 00 00 00 00 00 00 00 01 03 00 00 00 00 00 00 00 00

  


00 00 00 00

  


02

  


03 00 00 00


02


32 33

  


01 00 00 00


00

  


05 00 00 00


04 7C 26 7C 30

  


01 00 00 00


00

  


C2 00 00 00


4A


0C 00


17 00


FF FF


02 00


FF FF FF FF


51 65 41 01


64 43 02 02


60 AE 0A 00


60 AE 0A 00


60 AE 0A 00


60 AE 0A 00


60 AE 0A 00


60 AE 0A 00


E5 01 06 00


01


01


01


00 00 00 00 00 80 66 40


01


00


00


00 00 00 00 02


A0 86 01 00


04


00


A0 86 01 00


40 0D 03 00


40 0D 03 00


00 00 00 00


40 9C 00 00


00


00 01 01 01 01 01


01


01


01 01 00


00 00 00 00 00 00 00 00


0F 00 03 01 00 00 00 40 9C 00 00 00 FD 59 30 C0 71 4B E9 44 89 E9 20 D2 B2 A8 B7 97 33 82 41 85 38 79 42 40 83 79 2B 1B 7D 37 65 33 00 00 00 00 FF FF FF 7F FF FF FF 7F 00 01 12 00 00 00 00 00 00 00 00 00 00 00 01 03 00 00 00 00 00 00 00 00

  


00 00 00 00

  


02

  


03 00 00 00


02


32 32

  


01 00 00 00


00

  


05 00 00 00


04 7C 26 7C 30

  


01 00 00 00


00

  


C2 00 00 00


4A


0C 00


08 00


FF FF


02 00


FF FF FF FF


51 65 41 01


24 01 F3 01


60 AE 0A 00


60 AE 0A 00


60 AE 0A 00


60 AE 0A 00


60 AE 0A 00


60 AE 0A 00


E5 01 06 00


01


01


01


00 00 00 00 00 80 66 40


01


00


00


00 00 00 00 02


A0 86 01 00


04


00


A0 86 01 00


40 0D 03 00


40 0D 03 00


00 00 00 00


40 9C 00 00


00


00 01 01 01 01 01


01


01


01 01 00


00 00 00 00 00 00 00 00


0F 00 03 01 00 00 00 40 9C 00 00 00 FD 59 30 C0 71 4B E9 44 89 E9 20 D2 B2 A8 B7 97 33 82 41 85 38 79 42 40 83 79 2B 1B 7D 37 65 33 00 00 00 00 FF FF FF 7F FF FF FF 7F 00 01 12 00 00 00 00 00 00 00 00 00 00 00 01 03 00 00 00 00 00 00 00 00

  


00 00 00 00

  


02

  


03 00 00 00


02


32 31

  


01 00 00 00


00

  


05 00 00 00


04 7C 26 7C 30

  


01 00 00 00


00

  


C2 00 00 00


4A


0C 00


04 00


FF FF


02 00


FF FF FF FF


51 65 41 01


E4 BE E3 01


60 AE 0A 00


60 AE 0A 00


60 AE 0A 00


60 AE 0A 00


60 AE 0A 00


60 AE 0A 00


E5 01 06 00


01


01


01


00 00 00 00 00 80 66 40


01


00


00


00 00 00 00 02


A0 86 01 00


04


00


A0 86 01 00


40 0D 03 00


40 0D 03 00


00 00 00 00


40 9C 00 00


00


00 01 01 01 01 01


01


01


01 01 00


00 00 00 00 00 00 00 00


0F 00 03 01 00 00 00 40 9C 00 00 00 FD 59 30 C0 71 4B E9 44 89 E9 20 D2 B2 A8 B7 97 33 82 41 85 38 79 42 40 83 79 2B 1B 7D 37 65 33 00 00 00 00 FF FF FF 7F FF FF FF 7F 00 01 12 00 00 00 00 00 00 00 00 00 00 00 01 03 00 00 00 00 00 00 00 00

  


00 00 00 00

  


02

  


03 00 00 00


02


32 30

  


01 00 00 00


00

  


05 00 00 00


04 7C 26 7C 30

  


01 00 00 00


00

  


C2 00 00 00


4A


0C 00


1B 00


FF FF


02 00


FF FF FF FF


51 65 41 01


A4 7C D4 01


60 AE 0A 00


60 AE 0A 00


60 AE 0A 00


60 AE 0A 00


60 AE 0A 00


60 AE 0A 00


E5 01 06 00


01


01


01


00 00 00 00 00 80 66 40


01


00


00


00 00 00 00 02


A0 86 01 00


04


00


A0 86 01 00


40 0D 03 00


40 0D 03 00


00 00 00 00


40 9C 00 00


00


00 01 01 01 01 01


01


01


01 01 00


00 00 00 00 00 00 00 00


0F 00 03 01 00 00 00 40 9C 00 00 00 FD 59 30 C0 71 4B E9 44 89 E9 20 D2 B2 A8 B7 97 33 82 41 85 38 79 42 40 83 79 2B 1B 7D 37 65 33 00 00 00 00 FF FF FF 7F FF FF FF 7F 00 01 12 00 00 00 00 00 00 00 00 00 00 00 01 03 00 00 00 00 00 00 00 00

  


00 00 00 00

  


02

  


03 00 00 00


02


31 39

  


01 00 00 00


00

  


05 00 00 00


04 7C 26 7C 30

  


01 00 00 00


00

  


C2 00 00 00


4A


0C 00


1B 00


FF FF


02 00


FF FF FF FF


51 65 41 01


64 3A C5 01


60 AE 0A 00


60 AE 0A 00


60 AE 0A 00


60 AE 0A 00


60 AE 0A 00


60 AE 0A 00


E5 01 06 00


01


01


01


00 00 00 00 00 80 66 40


01


00


00


00 00 00 00 02


A0 86 01 00


04


00


A0 86 01 00


40 0D 03 00


40 0D 03 00


00 00 00 00


40 9C 00 00


00


00 01 01 01 01 01


01


01


01 01 00


00 00 00 00 00 00 00 00


0F 00 03 01 00 00 00 40 9C 00 00 00 FD 59 30 C0 71 4B E9 44 89 E9 20 D2 B2 A8 B7 97 33 82 41 85 38 79 42 40 83 79 2B 1B 7D 37 65 33 00 00 00 00 FF FF FF 7F FF FF FF 7F 00 01 12 00 00 00 00 00 00 00 00 00 00 00 01 03 00 00 00 00 00 00 00 00

  


00 00 00 00

  


02

  


03 00 00 00


02


31 38

  


01 00 00 00


00

  


05 00 00 00


04 7C 26 7C 30

  


01 00 00 00


00

  


C2 00 00 00


4A


0C 00


22 00


FF FF


02 00


FF FF FF FF


51 65 41 01


24 F8 B5 01


60 AE 0A 00


60 AE 0A 00


60 AE 0A 00


60 AE 0A 00


60 AE 0A 00


60 AE 0A 00


E5 01 06 00


01


01


01


00 00 00 00 00 80 66 40


01


00


00


00 00 00 00 02


A0 86 01 00


04


00


A0 86 01 00


40 0D 03 00


40 0D 03 00


00 00 00 00


40 9C 00 00


00


00 01 01 01 01 01


01


01


01 01 00


00 00 00 00 00 00 00 00


0F 00 03 01 00 00 00 40 9C 00 00 00 FD 59 30 C0 71 4B E9 44 89 E9 20 D2 B2 A8 B7 97 33 82 41 85 38 79 42 40 83 79 2B 1B 7D 37 65 33 00 00 00 00 FF FF FF 7F FF FF FF 7F 00 01 12 00 00 00 00 00 00 00 00 00 00 00 01 03 00 00 00 00 00 00 00 00

  


00 00 00 00

  


02

  


03 00 00 00


02


31 37

  


01 00 00 00


00

  


05 00 00 00


04 7C 26 7C 30

  


01 00 00 00


00

  


C2 00 00 00


4A


0C 00


21 00


FF FF


02 00


FF FF FF FF


51 65 41 01


E4 B5 A6 01


60 AE 0A 00


60 AE 0A 00


60 AE 0A 00


60 AE 0A 00


60 AE 0A 00


60 AE 0A 00


E5 01 06 00


01


01


01


00 00 00 00 00 80 66 40


01


00


00


00 00 00 00 02


A0 86 01 00


04


00


A0 86 01 00


40 0D 03 00


40 0D 03 00


00 00 00 00


40 9C 00 00


00


00 01 01 01 01 01


01


01


01 01 00


00 00 00 00 00 00 00 00


0F 00 03 01 00 00 00 40 9C 00 00 00 FD 59 30 C0 71 4B E9 44 89 E9 20 D2 B2 A8 B7 97 33 82 41 85 38 79 42 40 83 79 2B 1B 7D 37 65 33 00 00 00 00 FF FF FF 7F FF FF FF 7F 00 01 12 00 00 00 00 00 00 00 00 00 00 00 01 03 00 00 00 00 00 00 00 00

  


00 00 00 00

  


02

  


03 00 00 00


02


31 36

  


01 00 00 00


00

  


05 00 00 00


04 7C 26 7C 30

  


01 00 00 00


00

  


C2 00 00 00


4A


0C 00


20 00


FF FF


02 00


FF FF FF FF


51 65 41 01


A4 73 97 01


60 AE 0A 00


60 AE 0A 00


60 AE 0A 00


60 AE 0A 00


60 AE 0A 00


60 AE 0A 00


E5 01 06 00


01


01


01


00 00 00 00 00 80 66 40


01


00


00


00 00 00 00 02


A0 86 01 00


04


00


A0 86 01 00


40 0D 03 00


40 0D 03 00


00 00 00 00


40 9C 00 00


00


00 01 01 01 01 01


01


01


01 01 00


00 00 00 00 00 00 00 00


0F 00 03 01 00 00 00 40 9C 00 00 00 FD 59 30 C0 71 4B E9 44 89 E9 20 D2 B2 A8 B7 97 33 82 41 85 38 79 42 40 83 79 2B 1B 7D 37 65 33 00 00 00 00 FF FF FF 7F FF FF FF 7F 00 01 12 00 00 00 00 00 00 00 00 00 00 00 01 03 00 00 00 00 00 00 00 00

  


00 00 00 00

  


02

  


03 00 00 00


02


31 35

  


01 00 00 00


00

  


05 00 00 00


04 7C 26 7C 30

  


01 00 00 00


00

  


C2 00 00 00


4A


0C 00


1F 00


FF FF


02 00


FF FF FF FF


51 65 41 01


64 31 88 01


60 AE 0A 00


60 AE 0A 00


60 AE 0A 00


60 AE 0A 00


60 AE 0A 00


60 AE 0A 00


E5 01 06 00


01


01


01


00 00 00 00 00 80 66 40


01


00


00


00 00 00 00 02


A0 86 01 00


04


00


A0 86 01 00


40 0D 03 00


40 0D 03 00


00 00 00 00


40 9C 00 00


00


00 01 01 01 01 01


01


01


01 01 00


00 00 00 00 00 00 00 00


0F 00 03 01 00 00 00 40 9C 00 00 00 FD 59 30 C0 71 4B E9 44 89 E9 20 D2 B2 A8 B7 97 33 82 41 85 38 79 42 40 83 79 2B 1B 7D 37 65 33 00 00 00 00 FF FF FF 7F FF FF FF 7F 00 01 12 00 00 00 00 00 00 00 00 00 00 00 01 03 00 00 00 00 00 00 00 00

  


00 00 00 00

  


02

  


03 00 00 00


02


31 34

  


01 00 00 00


00

  


05 00 00 00


04 7C 26 7C 30

  


01 00 00 00


00

  


C2 00 00 00


4A


0C 00


19 00


FF FF


02 00


FF FF FF FF


51 65 41 01


24 EF 78 01


60 AE 0A 00


60 AE 0A 00


60 AE 0A 00


60 AE 0A 00


60 AE 0A 00


60 AE 0A 00


E5 01 06 00


01


01


01


00 00 00 00 00 80 66 40


01


00


00


00 00 00 00 02


A0 86 01 00


04


00


A0 86 01 00


40 0D 03 00


40 0D 03 00


00 00 00 00


40 9C 00 00


00


00 01 01 01 01 01


01


01


01 01 00


00 00 00 00 00 00 00 00


0F 00 03 01 00 00 00 40 9C 00 00 00 FD 59 30 C0 71 4B E9 44 89 E9 20 D2 B2 A8 B7 97 33 82 41 85 38 79 42 40 83 79 2B 1B 7D 37 65 33 00 00 00 00 FF FF FF 7F FF FF FF 7F 00 01 12 00 00 00 00 00 00 00 00 00 00 00 01 03 00 00 00 00 00 00 00 00

  


00 00 00 00

  


02

  


03 00 00 00


02


31 33

  


01 00 00 00


00

  


05 00 00 00


04 7C 26 7C 30

  


01 00 00 00


00

  


C2 00 00 00


4A


0C 00


33 00


FF FF


02 00


FF FF FF FF


51 65 41 01


E4 AC 69 01


60 AE 0A 00


60 AE 0A 00


60 AE 0A 00


60 AE 0A 00


60 AE 0A 00


60 AE 0A 00


E5 01 06 00


01


01


01


00 00 00 00 00 80 66 40


01


00


00


00 00 00 00 02


A0 86 01 00


04


00


A0 86 01 00


40 0D 03 00


40 0D 03 00


00 00 00 00


40 9C 00 00


00


00 01 01 01 01 01


01


01


01 01 00


00 00 00 00 00 00 00 00


0F 00 03 01 00 00 00 40 9C 00 00 00 FD 59 30 C0 71 4B E9 44 89 E9 20 D2 B2 A8 B7 97 33 82 41 85 38 79 42 40 83 79 2B 1B 7D 37 65 33 00 00 00 00 FF FF FF 7F FF FF FF 7F 00 01 12 00 00 00 00 00 00 00 00 00 00 00 01 03 00 00 00 00 00 00 00 00

  


00 00 00 00

  


02

  


03 00 00 00


02


31 32

  


01 00 00 00


00

  


05 00 00 00


04 7C 26 7C 30

  


01 00 00 00


00

  


C2 00 00 00


4A


0C 00


30 00


FF FF


02 00


FF FF FF FF


51 65 41 01


A4 6A 5A 01


60 AE 0A 00


60 AE 0A 00


60 AE 0A 00


60 AE 0A 00


60 AE 0A 00


60 AE 0A 00


E5 01 06 00


01


01


01


00 00 00 00 00 80 66 40


01


00


00


00 00 00 00 02


A0 86 01 00


04


00


A0 86 01 00


40 0D 03 00


40 0D 03 00


00 00 00 00


40 9C 00 00


00


00 01 01 01 01 01


01


01


01 01 00


00 00 00 00 00 00 00 00


0F 00 03 01 00 00 00 40 9C 00 00 00 FD 59 30 C0 71 4B E9 44 89 E9 20 D2 B2 A8 B7 97 33 82 41 85 38 79 42 40 83 79 2B 1B 7D 37 65 33 00 00 00 00 FF FF FF 7F FF FF FF 7F 00 01 12 00 00 00 00 00 00 00 00 00 00 00 01 03 00 00 00 00 00 00 00 00

  


00 00 00 00

  


02

  


03 00 00 00


02


31 31

  


01 00 00 00


00

  


05 00 00 00


04 7C 26 7C 30

  


01 00 00 00


00

  


C2 00 00 00


4A


0C 00


2F 00


FF FF


02 00


FF FF FF FF


51 65 41 01


64 28 4B 01


60 AE 0A 00


60 AE 0A 00


60 AE 0A 00


60 AE 0A 00


60 AE 0A 00


60 AE 0A 00


E5 01 06 00


01


01


01


00 00 00 00 00 80 66 40


01


00


00


00 00 00 00 02


A0 86 01 00


04


00


A0 86 01 00


40 0D 03 00


40 0D 03 00


00 00 00 00


40 9C 00 00


00


00 01 01 01 01 01


01


01


01 01 00


00 00 00 00 00 00 00 00


0F 00 03 01 00 00 00 40 9C 00 00 00 FD 59 30 C0 71 4B E9 44 89 E9 20 D2 B2 A8 B7 97 33 82 41 85 38 79 42 40 83 79 2B 1B 7D 37 65 33 00 00 00 00 FF FF FF 7F FF FF FF 7F 00 01 12 00 00 00 00 00 00 00 00 00 00 00 01 03 00 00 00 00 00 00 00 00

  


00 00 00 00

  


02

  


03 00 00 00


02


31 30

  


01 00 00 00


00

  


05 00 00 00


04 7C 26 7C 30

  


01 00 00 00


00

  


C2 00 00 00


4A


0C 00


2E 00


FF FF


02 00


FF FF FF FF


51 65 41 01


24 E6 3B 01


60 AE 0A 00


60 AE 0A 00


60 AE 0A 00


60 AE 0A 00


60 AE 0A 00


60 AE 0A 00


E5 01 06 00


01


01


01


00 00 00 00 00 80 66 40


01


00


00


00 00 00 00 02


A0 86 01 00


04


00


A0 86 01 00


40 0D 03 00


40 0D 03 00


00 00 00 00


40 9C 00 00


00


00 01 01 01 01 01


01


01


01 01 00


00 00 00 00 00 00 00 00


0F 00 03 01 00 00 00 40 9C 00 00 00 FD 59 30 C0 71 4B E9 44 89 E9 20 D2 B2 A8 B7 97 33 82 41 85 38 79 42 40 83 79 2B 1B 7D 37 65 33 00 00 00 00 FF FF FF 7F FF FF FF 7F 00 01 12 00 00 00 00 00 00 00 00 00 00 00 01 03 00 00 00 00 00 00 00 00

  


00 00 00 00

  


02

  


02 00 00 00


01


39

  


01 00 00 00


00

  


05 00 00 00


04 7C 26 7C 30

  


01 00 00 00


00

  


C2 00 00 00


4A


0C 00


2D 00


FF FF


02 00


FF FF FF FF


51 65 41 01


E4 A3 2C 01


60 AE 0A 00


60 AE 0A 00


60 AE 0A 00


60 AE 0A 00


60 AE 0A 00


60 AE 0A 00


E5 01 06 00


01


01


01


00 00 00 00 00 80 66 40


01


00


00


00 00 00 00 02


A0 86 01 00


04


00


A0 86 01 00


40 0D 03 00


40 0D 03 00


00 00 00 00


40 9C 00 00


00


00 01 01 01 01 01


01


01


01 01 00


00 00 00 00 00 00 00 00


0F 00 03 01 00 00 00 40 9C 00 00 00 FD 59 30 C0 71 4B E9 44 89 E9 20 D2 B2 A8 B7 97 33 82 41 85 38 79 42 40 83 79 2B 1B 7D 37 65 33 00 00 00 00 FF FF FF 7F FF FF FF 7F 00 01 12 00 00 00 00 00 00 00 00 00 00 00 01 03 00 00 00 00 00 00 00 00

  


00 00 00 00

  


02

  


02 00 00 00


01


38

  


01 00 00 00


00

  


05 00 00 00


04 7C 26 7C 30

  


01 00 00 00


00

  


C2 00 00 00


4A


0C 00


2C 00


FF FF


02 00


FF FF FF FF


51 65 41 01


A4 61 1D 01


60 AE 0A 00


60 AE 0A 00


60 AE 0A 00


60 AE 0A 00


60 AE 0A 00


60 AE 0A 00


E5 01 06 00


01


01


01


00 00 00 00 00 80 66 40


01


00


00


00 00 00 00 02


A0 86 01 00


04


00


A0 86 01 00


40 0D 03 00


40 0D 03 00


00 00 00 00


40 9C 00 00


00


00 01 01 01 01 01


01


01


01 01 00


00 00 00 00 00 00 00 00


0F 00 03 01 00 00 00 40 9C 00 00 00 FD 59 30 C0 71 4B E9 44 89 E9 20 D2 B2 A8 B7 97 33 82 41 85 38 79 42 40 83 79 2B 1B 7D 37 65 33 00 00 00 00 FF FF FF 7F FF FF FF 7F 00 01 12 00 00 00 00 00 00 00 00 00 00 00 01 03 00 00 00 00 00 00 00 00

  


00 00 00 00

  


02

  


02 00 00 00


01


37

  


01 00 00 00


00

  


05 00 00 00


04 7C 26 7C 30

  


01 00 00 00


00

  


C2 00 00 00


4A


0C 00


2B 00


FF FF


02 00


FF FF FF FF


51 65 41 01


64 1F 0E 01


60 AE 0A 00


60 AE 0A 00


60 AE 0A 00


60 AE 0A 00


60 AE 0A 00


60 AE 0A 00


E5 01 06 00


01


01


01


00 00 00 00 00 80 66 40


01


00


00


00 00 00 00 02


A0 86 01 00


04


00


A0 86 01 00


40 0D 03 00


40 0D 03 00


00 00 00 00


40 9C 00 00


00


00 01 01 01 01 01


01


01


01 01 00


00 00 00 00 00 00 00 00


0F 00 03 01 00 00 00 40 9C 00 00 00 FD 59 30 C0 71 4B E9 44 89 E9 20 D2 B2 A8 B7 97 33 82 41 85 38 79 42 40 83 79 2B 1B 7D 37 65 33 00 00 00 00 FF FF FF 7F FF FF FF 7F 00 01 12 00 00 00 00 00 00 00 00 00 00 00 01 03 00 00 00 00 00 00 00 00

  


00 00 00 00

  


02

  


02 00 00 00


01


36

  


01 00 00 00


00

  


05 00 00 00


04 7C 26 7C 30

  


01 00 00 00


00

  


C2 00 00 00


4A


0C 00


2A 00


FF FF


02 00


FF FF FF FF


51 65 41 01


24 DD FE 00


60 AE 0A 00


60 AE 0A 00


60 AE 0A 00


60 AE 0A 00


60 AE 0A 00


60 AE 0A 00


E5 01 06 00


01


01


01


00 00 00 00 00 80 66 40


01


00


00


00 00 00 00 02


A0 86 01 00


04


00


A0 86 01 00


40 0D 03 00


40 0D 03 00


00 00 00 00


40 9C 00 00


00


00 01 01 01 01 01


01


01


01 01 00


00 00 00 00 00 00 00 00


0F 00 03 01 00 00 00 40 9C 00 00 00 FD 59 30 C0 71 4B E9 44 89 E9 20 D2 B2 A8 B7 97 33 82 41 85 38 79 42 40 83 79 2B 1B 7D 37 65 33 00 00 00 00 FF FF FF 7F FF FF FF 7F 00 01 12 00 00 00 00 00 00 00 00 00 00 00 01 03 00 00 00 00 00 00 00 00

  


00 00 00 00

  


02

  


02 00 00 00


01


35

  


01 00 00 00


00

  


05 00 00 00


04 7C 26 7C 30

  


01 00 00 00


00

  


C2 00 00 00


4A


0C 00


29 00


FF FF


02 00


FF FF FF FF


51 65 41 01


E4 9A EF 00


60 AE 0A 00


60 AE 0A 00


60 AE 0A 00


60 AE 0A 00


60 AE 0A 00


60 AE 0A 00


E5 01 06 00


01


01


01


00 00 00 00 00 80 66 40


01


00


00


00 00 00 00 02


A0 86 01 00


04


00


A0 86 01 00


40 0D 03 00


40 0D 03 00


00 00 00 00


40 9C 00 00


00


00 01 01 01 01 01


01


01


01 01 00


00 00 00 00 00 00 00 00


0F 00 03 01 00 00 00 40 9C 00 00 00 FD 59 30 C0 71 4B E9 44 89 E9 20 D2 B2 A8 B7 97 33 82 41 85 38 79 42 40 83 79 2B 1B 7D 37 65 33 00 00 00 00 FF FF FF 7F FF FF FF 7F 00 01 12 00 00 00 00 00 00 00 00 00 00 00 01 03 00 00 00 00 00 00 00 00

  


00 00 00 00

  


02

  


02 00 00 00


01


34

  


01 00 00 00


00

  


05 00 00 00


04 7C 26 7C 30

  


01 00 00 00


00

  


C2 00 00 00


4A


0C 00


28 00


FF FF


02 00


FF FF FF FF


51 65 41 01


A4 58 E0 00


60 AE 0A 00


60 AE 0A 00


60 AE 0A 00


60 AE 0A 00


60 AE 0A 00


60 AE 0A 00


E5 01 06 00


01


01


01


00 00 00 00 00 80 66 40


01


00


00


00 00 00 00 02


A0 86 01 00


04


00


A0 86 01 00


40 0D 03 00


40 0D 03 00


00 00 00 00


40 9C 00 00


00


00 01 01 01 01 01


01


01


01 01 00


00 00 00 00 00 00 00 00


0F 00 03 01 00 00 00 40 9C 00 00 00 FD 59 30 C0 71 4B E9 44 89 E9 20 D2 B2 A8 B7 97 33 82 41 85 38 79 42 40 83 79 2B 1B 7D 37 65 33 00 00 00 00 FF FF FF 7F FF FF FF 7F 00 01 12 00 00 00 00 00 00 00 00 00 00 00 01 03 00 00 00 00 00 00 00 00

  


00 00 00 00

  


02

  


02 00 00 00


01


33

  


01 00 00 00


00

  


05 00 00 00


04 7C 26 7C 30

  


01 00 00 00


00

  


C2 00 00 00


4A


0C 00


27 00


FF FF


02 00


FF FF FF FF


51 65 41 01


64 16 D1 00


60 AE 0A 00


60 AE 0A 00


60 AE 0A 00


60 AE 0A 00


60 AE 0A 00


60 AE 0A 00


E5 01 06 00


01


01


01


00 00 00 00 00 80 66 40


01


00


00


00 00 00 00 02


A0 86 01 00


04


00


A0 86 01 00


40 0D 03 00


40 0D 03 00


00 00 00 00


40 9C 00 00


00


00 01 01 01 01 01


01


01


01 01 00


00 00 00 00 00 00 00 00


0F 00 03 01 00 00 00 40 9C 00 00 00 FD 59 30 C0 71 4B E9 44 89 E9 20 D2 B2 A8 B7 97 33 82 41 85 38 79 42 40 83 79 2B 1B 7D 37 65 33 00 00 00 00 FF FF FF 7F FF FF FF 7F 00 01 12 00 00 00 00 00 00 00 00 00 00 00 01 03 00 00 00 00 00 00 00 00

  


00 00 00 00

  


02

  


02 00 00 00


01


32

  


01 00 00 00


00

  


05 00 00 00


04 7C 26 7C 30

  


01 00 00 00


00

  


C2 00 00 00


4A


0C 00


00 00


FF FF


02 00


FF FF FF FF


51 65 41 01


24 D4 C1 00


60 AE 0A 00


60 AE 0A 00


60 AE 0A 00


60 AE 0A 00


60 AE 0A 00


60 AE 0A 00


E5 01 06 00


01


01


01


00 00 00 00 00 80 66 40


01


00


00


00 00 00 00 02


A0 86 01 00


04


00


A0 86 01 00


40 0D 03 00


40 0D 03 00


00 00 00 00


40 9C 00 00


00


00 01 01 01 01 01


01


01


01 01 00


00 00 00 00 00 00 00 00


0F 00 03 01 00 00 00 40 9C 00 00 00 FD 59 30 C0 71 4B E9 44 89 E9 20 D2 B2 A8 B7 97 33 82 41 85 38 79 42 40 83 79 2B 1B 7D 37 65 33 00 00 00 00 FF FF FF 7F FF FF FF 7F 00 01 12 00 00 00 00 00 00 00 00 00 00 00 01 03 00 00 00 00 00 00 00 00

  


00 00 00 00

  


02

  


02 00 00 00


01


31

  


01 00 00 00


00

  


05 00 00 00


04 7C 26 7C 30

  


01 00 00 00


00

  


C2 00 00 00


4A


0C 00


19 00


FF FF


02 00


FF FF FF FF


51 65 41 01


E4 91 B2 00


60 AE 0A 00


60 AE 0A 00


60 AE 0A 00


60 AE 0A 00


60 AE 0A 00


60 AE 0A 00


E5 01 06 00


01


01


01


00 00 00 00 00 80 66 40


01


00


00


00 00 00 00 02


A0 86 01 00


04


00


A0 86 01 00


40 0D 03 00


40 0D 03 00


00 00 00 00


40 9C 00 00


00


00 01 01 01 01 01


01


01


01 01 00


00 00 00 00 00 00 00 00


0F 00 03 01 00 00 00 40 9C 00 00 00 FD 59 30 C0 71 4B E9 44 89 E9 20 D2 B2 A8 B7 97 33 82 41 85 38 79 42 40 83 79 2B 1B 7D 37 65 33 00 00 00 00 FF FF FF 7F FF FF FF 7F 00 01 12 00 00 00 00 00 00 00 00 00 00 00 01 03 00 00 00 00 00 00 00 00

  


00 00 00 00

  


02

  


02 00 00 00


01


30

  


01 00 00 00


00

  


05 00 00 00


04 7C 26 7C 30

  


01 00 00 00


00

  


C2 00 00 00


4A


0C 00


11 00


FF FF


02 00


FF FF FF FF


51 65 41 01


A4 4F A3 00


60 AE 0A 00


60 AE 0A 00


60 AE 0A 00


60 AE 0A 00


60 AE 0A 00


60 AE 0A 00


E5 01 06 00


01


01


01


00 00 00 00 00 80 66 40


01


00


00


00 00 00 00 02


A0 86 01 00


04


00


A0 86 01 00


40 0D 03 00


40 0D 03 00


00 00 00 00


40 9C 00 00


00


00 01 01 01 01 01


01


01


01 01 00


00 00 00 00 00 00 00 00


0F 00 03 01 00 00 00 40 9C 00 00 00 FD 59 30 C0 71 4B E9 44 89 E9 20 D2 B2 A8 B7 97 33 82 41 85 38 79 42 40 83 79 2B 1B 7D 37 65 33 00 00 00 00 FF FF FF 7F FF FF FF 7F 00 01 12 00 00 00 00 00 00 00 00 00 00 00 01 03 00 00 00 00 00 00 00 00

  


00 00 00 00

  


02

  


03 00 00 00


02


32 33

  


01 00 00 00


00

  


05 00 00 00


04 7C 26 7C 30

  


01 00 00 00


00

  


C2 00 00 00


4A


0C 00


0F 00


FF FF


03 00


FF FF FF FF


D1 F2 9C 01


64 43 02 02


60 AE 0A 00


60 AE 0A 00


60 AE 0A 00


60 AE 0A 00


60 AE 0A 00


60 AE 0A 00


E5 01 06 00


01


01


01


00 00 00 00 00 80 66 40


01


00


00


00 00 00 00 02


A0 86 01 00


04


00


A0 86 01 00


40 0D 03 00


40 0D 03 00


00 00 00 00


40 9C 00 00


00


00 01 01 01 01 01


01


01


01 01 00


00 00 00 00 00 00 00 00


0F 00 03 01 00 00 00 40 9C 00 00 00 FD 59 30 C0 71 4B E9 44 89 E9 20 D2 B2 A8 B7 97 33 82 41 85 38 79 42 40 83 79 2B 1B 7D 37 65 33 00 00 00 00 FF FF FF 7F FF FF FF 7F 00 01 12 00 00 00 00 00 00 00 00 00 00 00 01 03 00 00 00 00 00 00 00 00

  


00 00 00 00

  


02

  


03 00 00 00


02


32 32

  


01 00 00 00


00

  


05 00 00 00


04 7C 26 7C 30

  


01 00 00 00


00

  


C2 00 00 00


4A


0C 00


1A 00


FF FF


03 00


FF FF FF FF


D1 F2 9C 01


24 01 F3 01


60 AE 0A 00


60 AE 0A 00


60 AE 0A 00


60 AE 0A 00


60 AE 0A 00


60 AE 0A 00


E5 01 06 00


01


01


01


00 00 00 00 00 80 66 40


01


00


00


00 00 00 00 02


A0 86 01 00


04


00


A0 86 01 00


40 0D 03 00


40 0D 03 00


00 00 00 00


40 9C 00 00


00


00 01 01 01 01 01


01


01


01 01 00


00 00 00 00 00 00 00 00


0F 00 03 01 00 00 00 40 9C 00 00 00 FD 59 30 C0 71 4B E9 44 89 E9 20 D2 B2 A8 B7 97 33 82 41 85 38 79 42 40 83 79 2B 1B 7D 37 65 33 00 00 00 00 FF FF FF 7F FF FF FF 7F 00 01 12 00 00 00 00 00 00 00 00 00 00 00 01 03 00 00 00 00 00 00 00 00

  


00 00 00 00

  


02

  


03 00 00 00


02


32 31

  


01 00 00 00


00

  


05 00 00 00


04 7C 26 7C 30

  


01 00 00 00


00

  


C2 00 00 00


4A


0C 00


1D 00


FF FF


03 00


FF FF FF FF


D1 F2 9C 01


E4 BE E3 01


60 AE 0A 00


60 AE 0A 00


60 AE 0A 00


60 AE 0A 00


60 AE 0A 00


60 AE 0A 00


E5 01 06 00


01


01


01


00 00 00 00 00 80 66 40


01


00


00


00 00 00 00 02


A0 86 01 00


04


00


A0 86 01 00


40 0D 03 00


40 0D 03 00


00 00 00 00


40 9C 00 00


00


00 01 01 01 01 01


01


01


01 01 00


00 00 00 00 00 00 00 00


0F 00 03 01 00 00 00 40 9C 00 00 00 FD 59 30 C0 71 4B E9 44 89 E9 20 D2 B2 A8 B7 97 33 82 41 85 38 79 42 40 83 79 2B 1B 7D 37 65 33 00 00 00 00 FF FF FF 7F FF FF FF 7F 00 01 12 00 00 00 00 00 00 00 00 00 00 00 01 03 00 00 00 00 00 00 00 00

  


00 00 00 00

  


02

  


03 00 00 00


02


32 30

  


01 00 00 00


00

  


05 00 00 00


04 7C 26 7C 30

  


01 00 00 00


00

  


C2 00 00 00


4A


0C 00


05 00


FF FF


03 00


FF FF FF FF


D1 F2 9C 01


A4 7C D4 01


60 AE 0A 00


60 AE 0A 00


60 AE 0A 00


60 AE 0A 00


60 AE 0A 00


60 AE 0A 00


E5 01 06 00


01


01


01


00 00 00 00 00 80 66 40


01


00


00


00 00 00 00 02


A0 86 01 00


04


00


A0 86 01 00


40 0D 03 00


40 0D 03 00


00 00 00 00


40 9C 00 00


00


00 01 01 01 01 01


01


01


01 01 00


00 00 00 00 00 00 00 00


0F 00 03 01 00 00 00 40 9C 00 00 00 FD 59 30 C0 71 4B E9 44 89 E9 20 D2 B2 A8 B7 97 33 82 41 85 38 79 42 40 83 79 2B 1B 7D 37 65 33 00 00 00 00 FF FF FF 7F FF FF FF 7F 00 01 12 00 00 00 00 00 00 00 00 00 00 00 01 03 00 00 00 00 00 00 00 00

  


00 00 00 00

  


02

  


03 00 00 00


02


31 39

  


01 00 00 00


00

  


05 00 00 00


04 7C 26 7C 30

  


01 00 00 00


00

  


C2 00 00 00


4A


0C 00


09 00


FF FF


03 00


FF FF FF FF


D1 F2 9C 01


64 3A C5 01


60 AE 0A 00


60 AE 0A 00


60 AE 0A 00


60 AE 0A 00


60 AE 0A 00


60 AE 0A 00


E5 01 06 00


01


01


01


00 00 00 00 00 80 66 40


01


00


00


00 00 00 00 02


A0 86 01 00


04


00


A0 86 01 00


40 0D 03 00


40 0D 03 00


00 00 00 00


40 9C 00 00


00


00 01 01 01 01 01


01


01


01 01 00


00 00 00 00 00 00 00 00


0F 00 03 01 00 00 00 40 9C 00 00 00 FD 59 30 C0 71 4B E9 44 89 E9 20 D2 B2 A8 B7 97 33 82 41 85 38 79 42 40 83 79 2B 1B 7D 37 65 33 00 00 00 00 FF FF FF 7F FF FF FF 7F 00 01 12 00 00 00 00 00 00 00 00 00 00 00 01 03 00 00 00 00 00 00 00 00

  


00 00 00 00

  


02

  


03 00 00 00


02


31 38

  


01 00 00 00


00

  


05 00 00 00


04 7C 26 7C 30

  


01 00 00 00


00

  


C2 00 00 00


4A


0C 00


23 00


FF FF


03 00


FF FF FF FF


D1 F2 9C 01


24 F8 B5 01


60 AE 0A 00


60 AE 0A 00


60 AE 0A 00


60 AE 0A 00


60 AE 0A 00


60 AE 0A 00


E5 01 06 00


01


01


01


00 00 00 00 00 80 66 40


01


00


00


00 00 00 00 02


A0 86 01 00


04


00


A0 86 01 00


40 0D 03 00


40 0D 03 00


00 00 00 00


40 9C 00 00


00


00 01 01 01 01 01


01


01


01 01 00


00 00 00 00 00 00 00 00


0F 00 03 01 00 00 00 40 9C 00 00 00 FD 59 30 C0 71 4B E9 44 89 E9 20 D2 B2 A8 B7 97 33 82 41 85 38 79 42 40 83 79 2B 1B 7D 37 65 33 00 00 00 00 FF FF FF 7F FF FF FF 7F 00 01 12 00 00 00 00 00 00 00 00 00 00 00 01 03 00 00 00 00 00 00 00 00

  


00 00 00 00

  


02

  


03 00 00 00


02


31 37

  


01 00 00 00


00

  


05 00 00 00


04 7C 26 7C 30

  


01 00 00 00


00

  


C2 00 00 00


4A


0C 00


24 00


FF FF


03 00


FF FF FF FF


D1 F2 9C 01


E4 B5 A6 01


60 AE 0A 00


60 AE 0A 00


60 AE 0A 00


60 AE 0A 00


60 AE 0A 00


60 AE 0A 00


E5 01 06 00


01


01


01


00 00 00 00 00 80 66 40


01


00


00


00 00 00 00 02


A0 86 01 00


04


00


A0 86 01 00


40 0D 03 00


40 0D 03 00


00 00 00 00


40 9C 00 00


00


00 01 01 01 01 01


01


01


01 01 00


00 00 00 00 00 00 00 00


0F 00 03 01 00 00 00 40 9C 00 00 00 FD 59 30 C0 71 4B E9 44 89 E9 20 D2 B2 A8 B7 97 33 82 41 85 38 79 42 40 83 79 2B 1B 7D 37 65 33 00 00 00 00 FF FF FF 7F FF FF FF 7F 00 01 12 00 00 00 00 00 00 00 00 00 00 00 01 03 00 00 00 00 00 00 00 00

  


00 00 00 00

  


02

  


03 00 00 00


02


31 36

  


01 00 00 00


00

  


05 00 00 00


04 7C 26 7C 30

  


01 00 00 00


00

  


C2 00 00 00


4A


0C 00


25 00


FF FF


03 00


FF FF FF FF


D1 F2 9C 01


A4 73 97 01


60 AE 0A 00


60 AE 0A 00


60 AE 0A 00


60 AE 0A 00


60 AE 0A 00


60 AE 0A 00


E5 01 06 00


01


01


01


00 00 00 00 00 80 66 40


01


00


00


00 00 00 00 02


A0 86 01 00


04


00


A0 86 01 00


40 0D 03 00


40 0D 03 00


00 00 00 00


40 9C 00 00


00


00 01 01 01 01 01


01


01


01 01 00


00 00 00 00 00 00 00 00


0F 00 03 01 00 00 00 40 9C 00 00 00 FD 59 30 C0 71 4B E9 44 89 E9 20 D2 B2 A8 B7 97 33 82 41 85 38 79 42 40 83 79 2B 1B 7D 37 65 33 00 00 00 00 FF FF FF 7F FF FF FF 7F 00 01 12 00 00 00 00 00 00 00 00 00 00 00 01 03 00 00 00 00 00 00 00 00

  


00 00 00 00

  


02

  


03 00 00 00


02


31 35

  


01 00 00 00


00

  


05 00 00 00


04 7C 26 7C 30

  


01 00 00 00


00

  


C2 00 00 00


4A


0C 00


26 00


FF FF


03 00


FF FF FF FF


D1 F2 9C 01


64 31 88 01


60 AE 0A 00


60 AE 0A 00


60 AE 0A 00


60 AE 0A 00


60 AE 0A 00


60 AE 0A 00


E5 01 06 00


01


01


01


00 00 00 00 00 80 66 40


01


00


00


00 00 00 00 02


A0 86 01 00


04


00


A0 86 01 00


40 0D 03 00


40 0D 03 00


00 00 00 00


40 9C 00 00


00


00 01 01 01 01 01


01


01


01 01 00


00 00 00 00 00 00 00 00


0F 00 03 01 00 00 00 40 9C 00 00 00 FD 59 30 C0 71 4B E9 44 89 E9 20 D2 B2 A8 B7 97 33 82 41 85 38 79 42 40 83 79 2B 1B 7D 37 65 33 00 00 00 00 FF FF FF 7F FF FF FF 7F 00 01 12 00 00 00 00 00 00 00 00 00 00 00 01 03 00 00 00 00 00 00 00 00

  


00 00 00 00

  


02

  


03 00 00 00


02


31 34

  


01 00 00 00


00

  


05 00 00 00


04 7C 26 7C 30

  


01 00 00 00


00

  


C2 00 00 00


4A


0C 00


32 00


FF FF


03 00


FF FF FF FF


D1 F2 9C 01


24 EF 78 01


60 AE 0A 00


60 AE 0A 00


60 AE 0A 00


60 AE 0A 00


60 AE 0A 00


60 AE 0A 00


E5 01 06 00


01


01


01


00 00 00 00 00 80 66 40


01


00


00


00 00 00 00 02


A0 86 01 00


04


00


A0 86 01 00


40 0D 03 00


40 0D 03 00


00 00 00 00


40 9C 00 00


00


00 01 01 01 01 01


01


01


01 01 00


00 00 00 00 00 00 00 00


0F 00 03 01 00 00 00 40 9C 00 00 00 FD 59 30 C0 71 4B E9 44 89 E9 20 D2 B2 A8 B7 97 33 82 41 85 38 79 42 40 83 79 2B 1B 7D 37 65 33 00 00 00 00 FF FF FF 7F FF FF FF 7F 00 01 12 00 00 00 00 00 00 00 00 00 00 00 01 03 00 00 00 00 00 00 00 00

  


00 00 00 00

  


02

  


03 00 00 00


02


31 33

  


01 00 00 00


00

  


05 00 00 00


04 7C 26 7C 30

  


01 00 00 00


00

  


C2 00 00 00


4A


0C 00


16 00


FF FF


03 00


FF FF FF FF


D1 F2 9C 01


E4 AC 69 01


60 AE 0A 00


60 AE 0A 00


60 AE 0A 00


60 AE 0A 00


60 AE 0A 00


60 AE 0A 00


E5 01 06 00


01


01


01


00 00 00 00 00 80 66 40


01


00


00


00 00 00 00 02


A0 86 01 00


04


00


A0 86 01 00


40 0D 03 00


40 0D 03 00


00 00 00 00


40 9C 00 00


00


00 01 01 01 01 01


01


01


01 01 00


00 00 00 00 00 00 00 00


0F 00 03 01 00 00 00 40 9C 00 00 00 FD 59 30 C0 71 4B E9 44 89 E9 20 D2 B2 A8 B7 97 33 82 41 85 38 79 42 40 83 79 2B 1B 7D 37 65 33 00 00 00 00 FF FF FF 7F FF FF FF 7F 00 01 12 00 00 00 00 00 00 00 00 00 00 00 01 03 00 00 00 00 00 00 00 00

  


00 00 00 00

  


02

  


03 00 00 00


02


31 32

  


01 00 00 00


00

  


05 00 00 00


04 7C 26 7C 30

  


01 00 00 00


00

  


C2 00 00 00


4A


0C 00


15 00


FF FF


03 00


FF FF FF FF


D1 F2 9C 01


A4 6A 5A 01


60 AE 0A 00


60 AE 0A 00


60 AE 0A 00


60 AE 0A 00


60 AE 0A 00


60 AE 0A 00


E5 01 06 00


01


01


01


00 00 00 00 00 80 66 40


01


00


00


00 00 00 00 02


A0 86 01 00


04


00


A0 86 01 00


40 0D 03 00


40 0D 03 00


00 00 00 00


40 9C 00 00


00


00 01 01 01 01 01


01


01


01 01 00


00 00 00 00 00 00 00 00


0F 00 03 01 00 00 00 40 9C 00 00 00 FD 59 30 C0 71 4B E9 44 89 E9 20 D2 B2 A8 B7 97 33 82 41 85 38 79 42 40 83 79 2B 1B 7D 37 65 33 00 00 00 00 FF FF FF 7F FF FF FF 7F 00 01 12 00 00 00 00 00 00 00 00 00 00 00 01 03 00 00 00 00 00 00 00 00

  


00 00 00 00

  


02

  


03 00 00 00


02


31 31

  


01 00 00 00


00

  


05 00 00 00


04 7C 26 7C 30

  


01 00 00 00


00

  


C2 00 00 00


4A


0C 00


1C 00


FF FF


03 00


FF FF FF FF


D1 F2 9C 01


64 28 4B 01


60 AE 0A 00


60 AE 0A 00


60 AE 0A 00


60 AE 0A 00


60 AE 0A 00


60 AE 0A 00


E5 01 06 00


01


01


01


00 00 00 00 00 80 66 40


01


00


00


00 00 00 00 02


A0 86 01 00


04


00


A0 86 01 00


40 0D 03 00


40 0D 03 00


00 00 00 00


40 9C 00 00


00


00 01 01 01 01 01


01


01


01 01 00


00 00 00 00 00 00 00 00


0F 00 03 01 00 00 00 40 9C 00 00 00 FD 59 30 C0 71 4B E9 44 89 E9 20 D2 B2 A8 B7 97 33 82 41 85 38 79 42 40 83 79 2B 1B 7D 37 65 33 00 00 00 00 FF FF FF 7F FF FF FF 7F 00 01 12 00 00 00 00 00 00 00 00 00 00 00 01 03 00 00 00 00 00 00 00 00

  


00 00 00 00

  


02

  


03 00 00 00


02


31 30

  


01 00 00 00


00

  


05 00 00 00


04 7C 26 7C 30

  


01 00 00 00


00

  


C2 00 00 00


4A


0C 00


0E 00


FF FF


03 00


FF FF FF FF


D1 F2 9C 01


24 E6 3B 01


60 AE 0A 00


60 AE 0A 00


60 AE 0A 00


60 AE 0A 00


60 AE 0A 00


60 AE 0A 00


E5 01 06 00


01


01


01


00 00 00 00 00 80 66 40


01


00


00


00 00 00 00 02


A0 86 01 00


04


00


A0 86 01 00


40 0D 03 00


40 0D 03 00


00 00 00 00


40 9C 00 00


00


00 01 01 01 01 01


01


01


01 01 00


00 00 00 00 00 00 00 00


0F 00 03 01 00 00 00 40 9C 00 00 00 FD 59 30 C0 71 4B E9 44 89 E9 20 D2 B2 A8 B7 97 33 82 41 85 38 79 42 40 83 79 2B 1B 7D 37 65 33 00 00 00 00 FF FF FF 7F FF FF FF 7F 00 01 12 00 00 00 00 00 00 00 00 00 00 00 01 03 00 00 00 00 00 00 00 00

  


00 00 00 00

  


02

  


02 00 00 00


01


39

  


01 00 00 00


00

  


05 00 00 00


04 7C 26 7C 30

  


01 00 00 00


00

  


C2 00 00 00


4A


0C 00


06 00


FF FF


03 00


FF FF FF FF


D1 F2 9C 01


E4 A3 2C 01


60 AE 0A 00


60 AE 0A 00


60 AE 0A 00


60 AE 0A 00


60 AE 0A 00


60 AE 0A 00


E5 01 06 00


01


01


01


00 00 00 00 00 80 66 40


01


00


00


00 00 00 00 02


A0 86 01 00


04


00


A0 86 01 00


40 0D 03 00


40 0D 03 00


00 00 00 00


40 9C 00 00


00


00 01 01 01 01 01


01


01


01 01 00


00 00 00 00 00 00 00 00


0F 00 03 01 00 00 00 40 9C 00 00 00 FD 59 30 C0 71 4B E9 44 89 E9 20 D2 B2 A8 B7 97 33 82 41 85 38 79 42 40 83 79 2B 1B 7D 37 65 33 00 00 00 00 FF FF FF 7F FF FF FF 7F 00 01 12 00 00 00 00 00 00 00 00 00 00 00 01 03 00 00 00 00 00 00 00 00

  


00 00 00 00

  


02

  


02 00 00 00


01


38

  


01 00 00 00


00

  


05 00 00 00


04 7C 26 7C 30

  


01 00 00 00


00

  


C2 00 00 00


4A


0C 00


0A 00


FF FF


03 00


FF FF FF FF


D1 F2 9C 01


A4 61 1D 01


60 AE 0A 00


60 AE 0A 00


60 AE 0A 00


60 AE 0A 00


60 AE 0A 00


60 AE 0A 00


E5 01 06 00


01


01


01


00 00 00 00 00 80 66 40


01


00


00


00 00 00 00 02


A0 86 01 00


04


00


A0 86 01 00


40 0D 03 00


40 0D 03 00


00 00 00 00


40 9C 00 00


00


00 01 01 01 01 01


01


01


01 01 00


00 00 00 00 00 00 00 00


0F 00 03 01 00 00 00 40 9C 00 00 00 FD 59 30 C0 71 4B E9 44 89 E9 20 D2 B2 A8 B7 97 33 82 41 85 38 79 42 40 83 79 2B 1B 7D 37 65 33 00 00 00 00 FF FF FF 7F FF FF FF 7F 00 01 12 00 00 00 00 00 00 00 00 00 00 00 01 03 00 00 00 00 00 00 00 00

  


00 00 00 00

  


02

  


02 00 00 00


01


37

  


01 00 00 00


00

  


05 00 00 00


04 7C 26 7C 30

  


01 00 00 00


00

  


C2 00 00 00


4A


0C 00


0D 00


FF FF


03 00


FF FF FF FF


D1 F2 9C 01


64 1F 0E 01


60 AE 0A 00


60 AE 0A 00


60 AE 0A 00


60 AE 0A 00


60 AE 0A 00


60 AE 0A 00


E5 01 06 00


01


01


01


00 00 00 00 00 80 66 40


01


00


00


00 00 00 00 02


A0 86 01 00


04


00


A0 86 01 00


40 0D 03 00


40 0D 03 00


00 00 00 00


40 9C 00 00


00


00 01 01 01 01 01


01


01


01 01 00


00 00 00 00 00 00 00 00


0F 00 03 01 00 00 00 40 9C 00 00 00 FD 59 30 C0 71 4B E9 44 89 E9 20 D2 B2 A8 B7 97 33 82 41 85 38 79 42 40 83 79 2B 1B 7D 37 65 33 00 00 00 00 FF FF FF 7F FF FF FF 7F 00 01 12 00 00 00 00 00 00 00 00 00 00 00 01 03 00 00 00 00 00 00 00 00

  


00 00 00 00

  


02

  


02 00 00 00


01


36

  


01 00 00 00


00

  


05 00 00 00


04 7C 26 7C 30

  


01 00 00 00


00

  


C2 00 00 00


4A


0C 00


02 00


FF FF


03 00


FF FF FF FF


D1 F2 9C 01


24 DD FE 00


60 AE 0A 00


60 AE 0A 00


60 AE 0A 00


60 AE 0A 00


60 AE 0A 00


60 AE 0A 00


E5 01 06 00


01


01


01


00 00 00 00 00 80 66 40


01


00


00


00 00 00 00 02


A0 86 01 00


04


00


A0 86 01 00


40 0D 03 00


40 0D 03 00


00 00 00 00


40 9C 00 00


00


00 01 01 01 01 01


01


01


01 01 00


00 00 00 00 00 00 00 00


0F 00 03 01 00 00 00 40 9C 00 00 00 FD 59 30 C0 71 4B E9 44 89 E9 20 D2 B2 A8 B7 97 33 82 41 85 38 79 42 40 83 79 2B 1B 7D 37 65 33 00 00 00 00 FF FF FF 7F FF FF FF 7F 00 01 12 00 00 00 00 00 00 00 00 00 00 00 01 03 00 00 00 00 00 00 00 00

  


00 00 00 00

  


02

  


02 00 00 00


01


35

  


01 00 00 00


00

  


05 00 00 00


04 7C 26 7C 30

  


01 00 00 00


00

  


C2 00 00 00


4A


0C 00


1E 00


FF FF


03 00


FF FF FF FF


D1 F2 9C 01


E4 9A EF 00


60 AE 0A 00


60 AE 0A 00


60 AE 0A 00


60 AE 0A 00


60 AE 0A 00


60 AE 0A 00


E5 01 06 00


01


01


01


00 00 00 00 00 80 66 40


01


00


00


00 00 00 00 02


A0 86 01 00


04


00


A0 86 01 00


40 0D 03 00


40 0D 03 00


00 00 00 00


40 9C 00 00


00


00 01 01 01 01 01


01


01


01 01 00


00 00 00 00 00 00 00 00


0F 00 03 01 00 00 00 40 9C 00 00 00 FD 59 30 C0 71 4B E9 44 89 E9 20 D2 B2 A8 B7 97 33 82 41 85 38 79 42 40 83 79 2B 1B 7D 37 65 33 00 00 00 00 FF FF FF 7F FF FF FF 7F 00 01 12 00 00 00 00 00 00 00 00 00 00 00 01 03 00 00 00 00 00 00 00 00

  


00 00 00 00

  


02

  


02 00 00 00


01


34

  


01 00 00 00


00

  


05 00 00 00


04 7C 26 7C 30

  


01 00 00 00


00

  


C2 00 00 00


4A


0C 00


18 00


FF FF


03 00


FF FF FF FF


D1 F2 9C 01


A4 58 E0 00


60 AE 0A 00


60 AE 0A 00


60 AE 0A 00


60 AE 0A 00


60 AE 0A 00


60 AE 0A 00


E5 01 06 00


01


01


01


00 00 00 00 00 80 66 40


01


00


00


00 00 00 00 02


A0 86 01 00


04


00


A0 86 01 00


40 0D 03 00


40 0D 03 00


00 00 00 00


40 9C 00 00


00


00 01 01 01 01 01


01


01


01 01 00


00 00 00 00 00 00 00 00


0F 00 03 01 00 00 00 40 9C 00 00 00 FD 59 30 C0 71 4B E9 44 89 E9 20 D2 B2 A8 B7 97 33 82 41 85 38 79 42 40 83 79 2B 1B 7D 37 65 33 00 00 00 00 FF FF FF 7F FF FF FF 7F 00 01 12 00 00 00 00 00 00 00 00 00 00 00 01 03 00 00 00 00 00 00 00 00

  


00 00 00 00

  


02

  


02 00 00 00


01


33

  


01 00 00 00


00

  


05 00 00 00


04 7C 26 7C 30

  


01 00 00 00


00

  


C2 00 00 00


4A


0C 00


0C 00


FF FF


03 00


FF FF FF FF


D1 F2 9C 01


64 16 D1 00


60 AE 0A 00


60 AE 0A 00


60 AE 0A 00


60 AE 0A 00


60 AE 0A 00


60 AE 0A 00


E5 01 06 00


01


01


01


00 00 00 00 00 80 66 40


01


00


00


00 00 00 00 02


A0 86 01 00


04


00


A0 86 01 00


40 0D 03 00


40 0D 03 00


00 00 00 00


40 9C 00 00


00


00 01 01 01 01 01


01


01


01 01 00


00 00 00 00 00 00 00 00


0F 00 03 01 00 00 00 40 9C 00 00 00 FD 59 30 C0 71 4B E9 44 89 E9 20 D2 B2 A8 B7 97 33 82 41 85 38 79 42 40 83 79 2B 1B 7D 37 65 33 00 00 00 00 FF FF FF 7F FF FF FF 7F 00 01 12 00 00 00 00 00 00 00 00 00 00 00 01 03 00 00 00 00 00 00 00 00

  


00 00 00 00

  


02

  


02 00 00 00


01


32

  


01 00 00 00


00

  


05 00 00 00


04 7C 26 7C 30

  


01 00 00 00


00

  


C2 00 00 00


4A


0C 00


07 00


FF FF


03 00


FF FF FF FF


D1 F2 9C 01


24 D4 C1 00


60 AE 0A 00


60 AE 0A 00


60 AE 0A 00


60 AE 0A 00


60 AE 0A 00


60 AE 0A 00


E5 01 06 00


01


01


01


00 00 00 00 00 80 66 40


01


00


00


00 00 00 00 02


A0 86 01 00


04


00


A0 86 01 00


40 0D 03 00


40 0D 03 00


00 00 00 00


40 9C 00 00


00


00 01 01 01 01 01


01


01


01 01 00


00 00 00 00 00 00 00 00


0F 00 03 01 00 00 00 40 9C 00 00 00 FD 59 30 C0 71 4B E9 44 89 E9 20 D2 B2 A8 B7 97 33 82 41 85 38 79 42 40 83 79 2B 1B 7D 37 65 33 00 00 00 00 FF FF FF 7F FF FF FF 7F 00 01 12 00 00 00 00 00 00 00 00 00 00 00 01 03 00 00 00 00 00 00 00 00

  


00 00 00 00

  


02

  


02 00 00 00


01


31

  


01 00 00 00


00

  


05 00 00 00


04 7C 26 7C 30

  


01 00 00 00


00

  


C2 00 00 00


4A


0C 00


0B 00


FF FF


03 00


FF FF FF FF


D1 F2 9C 01


E4 91 B2 00


60 AE 0A 00


60 AE 0A 00


60 AE 0A 00


60 AE 0A 00


60 AE 0A 00


60 AE 0A 00


E5 01 06 00


01


01


01


00 00 00 00 00 80 66 40


01


00


00


00 00 00 00 02


A0 86 01 00


04


00


A0 86 01 00


40 0D 03 00


40 0D 03 00


00 00 00 00


40 9C 00 00


00


00 01 01 01 01 01


01


01


01 01 00


00 00 00 00 00 00 00 00


0F 00 03 01 00 00 00 40 9C 00 00 00 FD 59 30 C0 71 4B E9 44 89 E9 20 D2 B2 A8 B7 97 33 82 41 85 38 79 42 40 83 79 2B 1B 7D 37 65 33 00 00 00 00 FF FF FF 7F FF FF FF 7F 00 01 12 00 00 00 00 00 00 00 00 00 00 00 01 03 00 00 00 00 00 00 00 00

  


00 00 00 00

  


02

  


02 00 00 00


01


30

  


01 00 00 00


00

  


05 00 00 00


04 7C 26 7C 30

  


01 00 00 00


00

  


C2 00 00 00


4A


0C 00


19 00


FF FF


03 00


FF FF FF FF


D1 F2 9C 01


A4 4F A3 00


60 AE 0A 00


60 AE 0A 00


60 AE 0A 00


60 AE 0A 00


60 AE 0A 00


60 AE 0A 00


E5 01 06 00


01


01


01


00 00 00 00 00 80 66 40


01


00


00


00 00 00 00 02


A0 86 01 00


04


00


A0 86 01 00


40 0D 03 00


40 0D 03 00


00 00 00 00


40 9C 00 00


00


00 01 01 01 01 01


01


01


01 01 00


00 00 00 00 00 00 00 00


0F 00 03 01 00 00 00 40 9C 00 00 00 FD 59 30 C0 71 4B E9 44 89 E9 20 D2 B2 A8 B7 97 33 82 41 85 38 79 42 40 83 79 2B 1B 7D 37 65 33 00 00 00 00 FF FF FF 7F FF FF FF 7F 00 01 12 00 00 00 00 00 00 00 00 00 00 00 01 03 00 00 00 00 00 00 00 00

  


00 00 00 00

  


02

  


07 00 00 00


06


53 48 49 45 4C 44

  


01 00 00 00


00

  


05 00 00 00


04 7C 26 7C 30

  


01 00 00 00


00

  


C2 00 00 00


4A


0C 00


19 00


FF FF


16 00


FF FF FF FF


A9 68 2F 02


5B 43 FE 00


A1 9C 09 00


A1 9C 09 00


A1 9C 09 00


A1 9C 09 00


A1 9C 09 00


A1 9C 09 00


46 11 06 00


01


01


01


00 00 00 00 00 80 56 40


01


00


00


00 00 00 00 02


A0 86 01 00


04


00


A0 86 01 00


40 0D 03 00


40 0D 03 00


00 00 00 00


40 9C 00 00


00


00 01 01 01 01 01


01


01


01 01 00


00 00 00 00 00 00 00 00


0F 00 03 01 00 00 00 40 9C 00 00 00 26 31 35 3C 2E 76 DB 4B B6 72 35 AB DA 3A 5E BE 33 82 41 85 38 79 42 40 83 79 2B 1B 7D 37 65 33 00 00 00 00 FF FF FF 7F FF FF FF 7F 00 01 12 00 00 00 00 00 00 00 00 00 00 00 01 03 00 00 00 00 00 00 00 00

  


00 00 00 00

  


02

  


07 00 00 00


06


53 48 49 45 4C 44

  


01 00 00 00


00

  


05 00 00 00


04 7C 26 7C 30

  


01 00 00 00


00

  


C2 00 00 00


4A


0C 00


19 00


FF FF


16 00


FF FF FF FF


A9 68 2F 02


9B 7C D0 00


A1 9C 09 00


A1 9C 09 00


A1 9C 09 00


A1 9C 09 00


A1 9C 09 00


A1 9C 09 00


46 11 06 00


01


01


01


00 00 00 00 00 80 56 40


01


00


00


00 00 00 00 02


A0 86 01 00


04


00


A0 86 01 00


40 0D 03 00


40 0D 03 00


00 00 00 00


40 9C 00 00


00


00 01 01 01 01 01


01


01


01 01 00


00 00 00 00 00 00 00 00


0F 00 03 01 00 00 00 40 9C 00 00 00 26 31 35 3C 2E 76 DB 4B B6 72 35 AB DA 3A 5E BE 33 82 41 85 38 79 42 40 83 79 2B 1B 7D 37 65 33 00 00 00 00 FF FF FF 7F FF FF FF 7F 00 01 12 00 00 00 00 00 00 00 00 00 00 00 01 03 00 00 00 00 00 00 00 00

  


00 00 00 00

  


02

  


02 00 00 00


01


34

  


01 00 00 00


00

  


05 00 00 00


04 7C 26 7C 30

  


01 00 00 00


00

  


C2 00 00 00


4A


0C 00


03 00


FF FF


16 00


FF FF FF FF


E9 A1 01 02


9B 7C D0 00


A1 9C 09 00


A1 9C 09 00


A1 9C 09 00


A1 9C 09 00


A1 9C 09 00


A1 9C 09 00


46 11 06 00


01


01


01


00 00 00 00 00 80 56 40


01


00


00


00 00 00 00 02


A0 86 01 00


04


00


A0 86 01 00


40 0D 03 00


40 0D 03 00


00 00 00 00


40 9C 00 00


00


00 01 01 01 01 01


01


01


01 01 00


00 00 00 00 00 00 00 00


0F 00 03 01 00 00 00 40 9C 00 00 00 26 31 35 3C 2E 76 DB 4B B6 72 35 AB DA 3A 5E BE 33 82 41 85 38 79 42 40 83 79 2B 1B 7D 37 65 33 00 00 00 00 FF FF FF 7F FF FF FF 7F 00 01 12 00 00 00 00 00 00 00 00 00 00 00 01 03 00 00 00 00 00 00 00 00

  


00 00 00 00

  


02

  


02 00 00 00


01


33

  


01 00 00 00


00

  


05 00 00 00


04 7C 26 7C 30

  


01 00 00 00


00

  


C2 00 00 00


4A


0C 00


19 00


FF FF


16 00


FF FF FF FF


E9 A1 01 02


DB BE DF 00


A1 9C 09 00


A1 9C 09 00


A1 9C 09 00


A1 9C 09 00


A1 9C 09 00


A1 9C 09 00


46 11 06 00


01


01


01


00 00 00 00 00 80 56 40


01


00


00


00 00 00 00 02


A0 86 01 00


04


00


A0 86 01 00


40 0D 03 00


40 0D 03 00


00 00 00 00


40 9C 00 00


00


00 01 01 01 01 01


01


01


01 01 00


00 00 00 00 00 00 00 00


0F 00 03 01 00 00 00 40 9C 00 00 00 26 31 35 3C 2E 76 DB 4B B6 72 35 AB DA 3A 5E BE 33 82 41 85 38 79 42 40 83 79 2B 1B 7D 37 65 33 00 00 00 00 FF FF FF 7F FF FF FF 7F 00 01 12 00 00 00 00 00 00 00 00 00 00 00 01 03 00 00 00 00 00 00 00 00

  


00 00 00 00

  


02

  


02 00 00 00


01


32

  


01 00 00 00


00

  


05 00 00 00


04 7C 26 7C 30

  


01 00 00 00


00

  


C2 00 00 00


4A


0C 00


FF FF


FF FF


16 00


FF FF FF FF


E9 A1 01 02


1B 01 EF 00


A1 9C 09 00


A1 9C 09 00


A1 9C 09 00


A1 9C 09 00


A1 9C 09 00


A1 9C 09 00


46 11 06 00


01


01


01


00 00 00 00 00 80 56 40


01


00


00


00 00 00 00 00


A0 86 01 00


04


00


A0 86 01 00


40 0D 03 00


40 0D 03 00


00 00 00 00


40 9C 00 00


00


00 00 01 01 01 01


01


01


01 00 00


00 00 00 00 00 00 00 00


0F 00 03 01 00 00 00 40 9C 00 00 00 26 31 35 3C 2E 76 DB 4B B6 72 35 AB DA 3A 5E BE 33 82 41 85 38 79 42 40 83 79 2B 1B 7D 37 65 33 00 00 00 00 FF FF FF 7F FF FF FF 7F 00 01 12 00 00 00 00 00 00 00 00 00 00 00 01 03 00 00 00 00 00 00 00 00

  


00 00 00 00

  


02

  


02 00 00 00


01


31

  


01 00 00 00


00

  


05 00 00 00


04 7C 26 7C 30

  


01 00 00 00


00

  


C2 00 00 00


4A


0C 00


31 00


FF FF


16 00


FF FF FF FF


E9 A1 01 02


5B 43 FE 00


A1 9C 09 00


A1 9C 09 00


A1 9C 09 00


A1 9C 09 00


A1 9C 09 00


A1 9C 09 00


46 11 06 00


01


01


01


00 00 00 00 00 80 56 40


01


00


00


00 00 00 00 02


A0 86 01 00


04


00


A0 86 01 00


40 0D 03 00


40 0D 03 00


00 00 00 00


40 9C 00 00


00


00 01 01 01 01 01


01


01


01 01 00


00 00 00 00 00 00 00 00


0F 00 03 01 00 00 00 40 9C 00 00 00 26 31 35 3C 2E 76 DB 4B B6 72 35 AB DA 3A 5E BE 33 82 41 85 38 79 42 40 83 79 2B 1B 7D 37 65 33 00 00 00 00 FF FF FF 7F FF FF FF 7F 00 01 12 00 00 00 00 00 00 00 00 00 00 00 01 03 00 00 00 00 00 00 00 00

  


00 00 00 00

  


02

  


02 00 00 00


01


30

  


01 00 00 00


00

  


05 00 00 00


04 7C 26 7C 30

  


01 00 00 00


00

  


C2 00 00 00


4A


0C 00


19 00


FF FF


07 00


FF FF FF FF


EA EC FB 01


1D B0 2E 02


CA 03 0C 00


CA 03 0C 00


CA 03 0C 00


CA 03 0C 00


CA 03 0C 00


CA 03 0C 00


D7 02 09 00


02


02


02


00 00 00 00 00 E0 70 40


01


00


00


00 00 00 00 02


A0 86 01 00


04


00


A0 86 01 00


40 0D 03 00


40 0D 03 00


00 00 00 00


40 9C 00 00


00


00 01 01 01 01 01


01


01


01 01 00


00 00 00 00 02 00 00 00


0F 00 03 01 00 00 00 40 9C 00 00 00 6E FD 22 25 EB 00 B2 40 81 A9 F9 8F 47 DC C3 F0 33 82 41 85 38 79 42 40 83 79 2B 1B 7D 37 65 33 00 00 00 00 FF FF FF 7F FF FF FF 7F 00 01 12 00 00 00 00 00 00 00 00 00 00 00 01 03 00 00 00 00 00 00 00 00

  


00 00 00 00

  


02

  


02 00 00 00


01


30

  


01 00 00 00


00

  


05 00 00 00


04 7C 26 7C 30

  


01 00 00 00


00

  


C2 00 00 00


4A


0C 00


19 00


FF FF


07 00


FF FF FF FF


EA EC FB 01


DB 0C 10 02


CA 03 0C 00


CA 03 0C 00


CA 03 0C 00


CA 03 0C 00


CA 03 0C 00


CA 03 0C 00


D7 02 09 00


02


02


02


00 00 00 00 00 E0 70 40


01


00


00


00 00 00 00 02


A0 86 01 00


04


00


A0 86 01 00


40 0D 03 00


40 0D 03 00


00 00 00 00


40 9C 00 00


00


00 01 01 01 01 01


01


01


01 01 00


00 00 00 00 02 00 00 00


0F 00 03 01 00 00 00 40 9C 00 00 00 6E FD 22 25 EB 00 B2 40 81 A9 F9 8F 47 DC C3 F0 33 82 41 85 38 79 42 40 83 79 2B 1B 7D 37 65 33 00 00 00 00 FF FF FF 7F FF FF FF 7F 00 01 12 00 00 00 00 00 00 00 00 00 00 00 01 03 00 00 00 00 00 00 00 00

  


00 00 00 00

  


02

  


02 00 00 00


01


30

  


01 00 00 00


00

  


05 00 00 00


04 7C 26 7C 30

  


01 00 00 00


00

  


C2 00 00 00


4A


0C 00


19 00


FF FF


07 00


FF FF FF FF


A8 49 DD 01


DB 0C 10 02


CA 03 0C 00


CA 03 0C 00


CA 03 0C 00


CA 03 0C 00


CA 03 0C 00


CA 03 0C 00


D7 02 09 00


02


02


02


00 00 00 00 00 E0 70 40


01


00


00


00 00 00 00 02


A0 86 01 00


04


00


A0 86 01 00


40 0D 03 00


40 0D 03 00


00 00 00 00


40 9C 00 00


00


00 01 01 01 01 01


01


01


01 01 00


00 00 00 00 02 00 00 00


0F 00 03 01 00 00 00 40 9C 00 00 00 6E FD 22 25 EB 00 B2 40 81 A9 F9 8F 47 DC C3 F0 33 82 41 85 38 79 42 40 83 79 2B 1B 7D 37 65 33 00 00 00 00 FF FF FF 7F FF FF FF 7F 00 01 12 00 00 00 00 00 00 00 00 00 00 00 01 03 00 00 00 00 00 00 00 00

  


00 00 00 00

  


02

  


02 00 00 00


01


30

  


01 00 00 00


00

  


05 00 00 00


04 7C 26 7C 30

  


01 00 00 00


00

  


C2 00 00 00


4A


0C 00


19 00


FF FF


07 00


FF FF FF FF


A8 49 DD 01


1D B0 2E 02


CA 03 0C 00


CA 03 0C 00


CA 03 0C 00


CA 03 0C 00


CA 03 0C 00


CA 03 0C 00


D7 02 09 00


02


02


02


00 00 00 00 00 E0 70 40


01


00


00


00 00 00 00 02


A0 86 01 00


04


00


A0 86 01 00


40 0D 03 00


40 0D 03 00


00 00 00 00


40 9C 00 00


00


00 01 01 01 01 01


01


01


01 01 00


00 00 00 00 02 00 00 00


0F 00 03 01 00 00 00 40 9C 00 00 00 6E FD 22 25 EB 00 B2 40 81 A9 F9 8F 47 DC C3 F0 33 82 41 85 38 79 42 40 83 79 2B 1B 7D 37 65 33 00 00 00 00 FF FF FF 7F FF FF FF 7F 00 01 12 00 00 00 00 00 00 00 00 00 00 00 01 03 00 00 00 00 00 00 00 00

  


00 00 00 00

  


02

  


02 00 00 00


01


31

  


01 00 00 00


00

  


05 00 00 00


04 7C 26 7C 30

  


01 00 00 00


00

  


C2 00 00 00


4A


0C 00


16 00


FF FF


07 00


FF FF FF FF


49 9B EC 01


7C 5E 1F 02


BC 04 0F 00


BC 04 0F 00


BC 04 0F 00


BC 04 0F 00


BC 04 0F 00


BC 04 0F 00


D7 02 09 00


01


01


01


00 00 00 00 00 E0 70 40


01


00


00


00 00 00 00 02


A0 86 01 00


04


00


A0 86 01 00


40 0D 03 00


40 0D 03 00


00 00 00 00


40 9C 00 00


00


00 01 01 01 01 01


01


01


01 01 00


00 00 00 00 00 00 00 00


0F 00 03 01 00 00 00 40 9C 00 00 00 44 A5 0C 20 F2 A6 C2 47 87 73 6D 35 80 A5 A2 9E 33 82 41 85 38 79 42 40 83 79 2B 1B 7D 37 65 33 00 00 00 00 FF FF FF 7F FF FF FF 7F 00 01 12 00 00 00 00 00 00 00 00 00 00 00 01 03 00 00 00 00 00 00 00 00

  


00 00 00 00

  


02

  


02 00 00 00


01


30

  


01 00 00 00


00

  


05 00 00 00


04 7C 26 7C 30

  


01 00 00 00


00

  


C2 00 00 00


4A


0C 00


19 00


FF FF


06 00


FF FF FF FF


90 A9 FD 00


AB 89 2E 02


CA 03 0C 00


CA 03 0C 00


CA 03 0C 00


CA 03 0C 00


CA 03 0C 00


CA 03 0C 00


D7 02 09 00


02


02


02


00 00 00 00 00 E0 70 40


01


00


00


00 00 00 00 02


A0 86 01 00


04


00


A0 86 01 00


40 0D 03 00


40 0D 03 00


00 00 00 00


40 9C 00 00


00


00 01 01 01 01 01


01


01


01 01 00


00 00 00 00 02 00 00 00


0F 00 03 01 00 00 00 40 9C 00 00 00 6E FD 22 25 EB 00 B2 40 81 A9 F9 8F 47 DC C3 F0 33 82 41 85 38 79 42 40 83 79 2B 1B 7D 37 65 33 00 00 00 00 FF FF FF 7F FF FF FF 7F 00 01 12 00 00 00 00 00 00 00 00 00 00 00 01 03 00 00 00 00 00 00 00 00

  


00 00 00 00

  


02

  


02 00 00 00


01


30

  


01 00 00 00


00

  


05 00 00 00


04 7C 26 7C 30

  


01 00 00 00


00

  


C2 00 00 00


4A


0C 00


19 00


FF FF


06 00


FF FF FF FF


90 A9 FD 00


69 E6 0F 02


CA 03 0C 00


CA 03 0C 00


CA 03 0C 00


CA 03 0C 00


CA 03 0C 00


CA 03 0C 00


D7 02 09 00


02


02


02


00 00 00 00 00 E0 70 40


01


00


00


00 00 00 00 02


A0 86 01 00


04


00


A0 86 01 00


40 0D 03 00


40 0D 03 00


00 00 00 00


40 9C 00 00


00


00 01 01 01 01 01


01


01


01 01 00


00 00 00 00 02 00 00 00


0F 00 03 01 00 00 00 40 9C 00 00 00 6E FD 22 25 EB 00 B2 40 81 A9 F9 8F 47 DC C3 F0 33 82 41 85 38 79 42 40 83 79 2B 1B 7D 37 65 33 00 00 00 00 FF FF FF 7F FF FF FF 7F 00 01 12 00 00 00 00 00 00 00 00 00 00 00 01 03 00 00 00 00 00 00 00 00

  


00 00 00 00

  


02

  


02 00 00 00


01


30

  


01 00 00 00


00

  


05 00 00 00


04 7C 26 7C 30

  


01 00 00 00


00

  


C2 00 00 00


4A


0C 00


19 00


FF FF


06 00


FF FF FF FF


4E 06 DF 00


69 E6 0F 02


CA 03 0C 00


CA 03 0C 00


CA 03 0C 00


CA 03 0C 00


CA 03 0C 00


CA 03 0C 00


D7 02 09 00


02


02


02


00 00 00 00 00 E0 70 40


01


00


00


00 00 00 00 02


A0 86 01 00


04


00


A0 86 01 00


40 0D 03 00


40 0D 03 00


00 00 00 00


40 9C 00 00


00


00 01 01 01 01 01


01


01


01 01 00


00 00 00 00 02 00 00 00


0F 00 03 01 00 00 00 40 9C 00 00 00 6E FD 22 25 EB 00 B2 40 81 A9 F9 8F 47 DC C3 F0 33 82 41 85 38 79 42 40 83 79 2B 1B 7D 37 65 33 00 00 00 00 FF FF FF 7F FF FF FF 7F 00 01 12 00 00 00 00 00 00 00 00 00 00 00 01 03 00 00 00 00 00 00 00 00

  


00 00 00 00

  


02

  


02 00 00 00


01


30

  


01 00 00 00


00

  


05 00 00 00


04 7C 26 7C 30

  


01 00 00 00


00

  


C2 00 00 00


4A


0C 00


19 00


FF FF


06 00


FF FF FF FF


4E 06 DF 00


AB 89 2E 02


CA 03 0C 00


CA 03 0C 00


CA 03 0C 00


CA 03 0C 00


CA 03 0C 00


CA 03 0C 00


D7 02 09 00


02


02


02


00 00 00 00 00 E0 70 40


01


00


00


00 00 00 00 02


A0 86 01 00


04


00


A0 86 01 00


40 0D 03 00


40 0D 03 00


00 00 00 00


40 9C 00 00


00


00 01 01 01 01 01


01


01


01 01 00


00 00 00 00 02 00 00 00


0F 00 03 01 00 00 00 40 9C 00 00 00 6E FD 22 25 EB 00 B2 40 81 A9 F9 8F 47 DC C3 F0 33 82 41 85 38 79 42 40 83 79 2B 1B 7D 37 65 33 00 00 00 00 FF FF FF 7F FF FF FF 7F 00 01 12 00 00 00 00 00 00 00 00 00 00 00 01 03 00 00 00 00 00 00 00 00

  


00 00 00 00

  


02

  


02 00 00 00


01


31

  


01 00 00 00


00

  


05 00 00 00


04 7C 26 7C 30

  


01 00 00 00


00

  


C2 00 00 00


4A


0C 00


0A 00


FF FF


06 00


FF FF FF FF


EF 57 EE 00


0A 38 1F 02


BC 04 0F 00


BC 04 0F 00


BC 04 0F 00


BC 04 0F 00


BC 04 0F 00


BC 04 0F 00


D7 02 09 00


01


01


01


00 00 00 00 00 E0 70 40


01


00


00


00 00 00 00 02


A0 86 01 00


04


00


A0 86 01 00


40 0D 03 00


40 0D 03 00


00 00 00 00


40 9C 00 00


00


00 01 01 01 01 01


01


01


01 01 00


00 00 00 00 00 00 00 00


0F 00 03 01 00 00 00 40 9C 00 00 00 44 A5 0C 20 F2 A6 C2 47 87 73 6D 35 80 A5 A2 9E 33 82 41 85 38 79 42 40 83 79 2B 1B 7D 37 65 33 00 00 00 00 FF FF FF 7F FF FF FF 7F 00 01 12 00 00 00 00 00 00 00 00 00 00 00 01 03 00 00 00 00 00 00 00 00

  


00 00 00 00

  


02

  


02 00 00 00


01


31

  


01 00 00 00


00

  


05 00 00 00


04 7C 26 7C 30

  


01 00 00 00


00

  


C2 00 00 00


4A


0C 00


31 00


FF FF


15 00


FF FF FF FF


E9 A1 01 02


FB 45 78 01


A1 9C 09 00


A1 9C 09 00


A1 9C 09 00


A1 9C 09 00


A1 9C 09 00


A1 9C 09 00


46 11 06 00


01


01


01


00 00 00 00 00 80 56 40


01


00


00


00 00 00 00 02


A0 86 01 00


04


00


A0 86 01 00


40 0D 03 00


40 0D 03 00


00 00 00 00


40 9C 00 00


00


00 01 01 01 01 01


01


01


01 01 00


00 00 00 00 00 00 00 00


0F 00 03 01 00 00 00 40 9C 00 00 00 26 31 35 3C 2E 76 DB 4B B6 72 35 AB DA 3A 5E BE 33 82 41 85 38 79 42 40 83 79 2B 1B 7D 37 65 33 00 00 00 00 FF FF FF 7F FF FF FF 7F 00 01 12 00 00 00 00 00 00 00 00 00 00 00 01 03 00 00 00 00 00 00 00 00

  


00 00 00 00

  


02

  


02 00 00 00


01


32

  


01 00 00 00


00

  


05 00 00 00


04 7C 26 7C 30

  


01 00 00 00


00

  


C2 00 00 00


4A


0C 00


FF FF


FF FF


15 00


FF FF FF FF


E9 A1 01 02


BB 03 69 01


A1 9C 09 00


A1 9C 09 00


A1 9C 09 00


A1 9C 09 00


A1 9C 09 00


A1 9C 09 00


46 11 06 00


01


01


01


00 00 00 00 00 80 56 40


01


00


00


00 00 00 00 00


A0 86 01 00


04


00


A0 86 01 00


40 0D 03 00


40 0D 03 00


00 00 00 00


40 9C 00 00


00


00 00 01 01 01 01


01


01


01 00 00


00 00 00 00 00 00 00 00


0F 00 03 01 00 00 00 40 9C 00 00 00 26 31 35 3C 2E 76 DB 4B B6 72 35 AB DA 3A 5E BE 33 82 41 85 38 79 42 40 83 79 2B 1B 7D 37 65 33 00 00 00 00 FF FF FF 7F FF FF FF 7F 00 01 12 00 00 00 00 00 00 00 00 00 00 00 01 03 00 00 00 00 00 00 00 00

  


00 00 00 00

  


02

  


02 00 00 00


01


33

  


01 00 00 00


00

  


05 00 00 00


04 7C 26 7C 30

  


01 00 00 00


00

  


C2 00 00 00


4A


0C 00


19 00


FF FF


15 00


FF FF FF FF


E9 A1 01 02


7B C1 59 01


A1 9C 09 00


A1 9C 09 00


A1 9C 09 00


A1 9C 09 00


A1 9C 09 00


A1 9C 09 00


46 11 06 00


01


01


01


00 00 00 00 00 80 56 40


01


00


00


00 00 00 00 02


A0 86 01 00


04


00


A0 86 01 00


40 0D 03 00


40 0D 03 00


00 00 00 00


40 9C 00 00


00


00 01 01 01 01 01


01


01


01 01 00


00 00 00 00 00 00 00 00


0F 00 03 01 00 00 00 40 9C 00 00 00 26 31 35 3C 2E 76 DB 4B B6 72 35 AB DA 3A 5E BE 33 82 41 85 38 79 42 40 83 79 2B 1B 7D 37 65 33 00 00 00 00 FF FF FF 7F FF FF FF 7F 00 01 12 00 00 00 00 00 00 00 00 00 00 00 01 03 00 00 00 00 00 00 00 00

  


00 00 00 00

  


02

  


02 00 00 00


01


34

  


01 00 00 00


00

  


05 00 00 00


04 7C 26 7C 30

  


01 00 00 00


00

  


C2 00 00 00


4A


0C 00


01 00


FF FF


15 00


FF FF FF FF


E9 A1 01 02


3B 7F 4A 01


A1 9C 09 00


A1 9C 09 00


A1 9C 09 00


A1 9C 09 00


A1 9C 09 00


A1 9C 09 00


46 11 06 00


01


01


01


00 00 00 00 00 80 56 40


01


00


00


00 00 00 00 02


A0 86 01 00


04


00


A0 86 01 00


40 0D 03 00


40 0D 03 00


00 00 00 00


40 9C 00 00


00


00 01 01 01 01 01


01


01


01 01 00


00 00 00 00 00 00 00 00


0F 00 03 01 00 00 00 40 9C 00 00 00 26 31 35 3C 2E 76 DB 4B B6 72 35 AB DA 3A 5E BE 33 82 41 85 38 79 42 40 83 79 2B 1B 7D 37 65 33 00 00 00 00 FF FF FF 7F FF FF FF 7F 00 01 12 00 00 00 00 00 00 00 00 00 00 00 01 03 00 00 00 00 00 00 00 00

  


00 00 00 00

  


02

  


07 00 00 00


06


53 48 49 45 4C 44

  


01 00 00 00


00

  


05 00 00 00


04 7C 26 7C 30

  


01 00 00 00


00

  


C2 00 00 00


4A


0C 00


19 00


FF FF


15 00


FF FF FF FF


A9 68 2F 02


3B 7F 4A 01


A1 9C 09 00


A1 9C 09 00


A1 9C 09 00


A1 9C 09 00


A1 9C 09 00


A1 9C 09 00


46 11 06 00


01


01


01


00 00 00 00 00 80 56 40


01


00


00


00 00 00 00 02


A0 86 01 00


04


00


A0 86 01 00


40 0D 03 00


40 0D 03 00


00 00 00 00


40 9C 00 00


00


00 01 01 01 01 01


01


01


01 01 00


00 00 00 00 00 00 00 00


0F 00 03 01 00 00 00 40 9C 00 00 00 26 31 35 3C 2E 76 DB 4B B6 72 35 AB DA 3A 5E BE 33 82 41 85 38 79 42 40 83 79 2B 1B 7D 37 65 33 00 00 00 00 FF FF FF 7F FF FF FF 7F 00 01 12 00 00 00 00 00 00 00 00 00 00 00 01 03 00 00 00 00 00 00 00 00

  


00 00 00 00

  


02

  


07 00 00 00


06


53 48 49 45 4C 44

  


01 00 00 00


00

  


05 00 00 00


04 7C 26 7C 30

  


01 00 00 00


00

  


C2 00 00 00


4A


0C 00


19 00


FF FF


15 00


FF FF FF FF


A9 68 2F 02


FB 45 78 01


A1 9C 09 00


A1 9C 09 00


A1 9C 09 00


A1 9C 09 00


A1 9C 09 00


A1 9C 09 00


46 11 06 00


01


01


01


00 00 00 00 00 80 56 40


01


00


00


00 00 00 00 02


A0 86 01 00


04


00


A0 86 01 00


40 0D 03 00


40 0D 03 00


00 00 00 00


40 9C 00 00


00


00 01 01 01 01 01


01


01


01 01 00


00 00 00 00 00 00 00 00


0F 00 03 01 00 00 00 40 9C 00 00 00 26 31 35 3C 2E 76 DB 4B B6 72 35 AB DA 3A 5E BE 33 82 41 85 38 79 42 40 83 79 2B 1B 7D 37 65 33 00 00 00 00 FF FF FF 7F FF FF FF 7F 00 01 12 00 00 00 00 00 00 00 00 00 00 00 01 03 00 00 00 00 00 00 00 00

  


00 00 00 00

  


02

  


07 00 00 00


06


53 48 49 45 4C 44

  


01 00 00 00


00

  


05 00 00 00


04 7C 26 7C 30

  


01 00 00 00


00

  


C2 00 00 00


4A


0C 00


19 00


FF FF


17 00


FF FF FF FF


A9 68 2F 02


AB 44 3B 01


A1 9C 09 00


A1 9C 09 00


A1 9C 09 00


A1 9C 09 00


A1 9C 09 00


A1 9C 09 00


46 11 06 00


01


01


01


00 00 00 00 00 80 56 40


01


00


00


00 00 00 00 02


A0 86 01 00


04


00


A0 86 01 00


40 0D 03 00


40 0D 03 00


00 00 00 00


40 9C 00 00


00


00 01 01 01 01 01


01


01


01 01 00


00 00 00 00 00 00 00 00


0F 00 03 01 00 00 00 40 9C 00 00 00 26 31 35 3C 2E 76 DB 4B B6 72 35 AB DA 3A 5E BE 33 82 41 85 38 79 42 40 83 79 2B 1B 7D 37 65 33 00 00 00 00 FF FF FF 7F FF FF FF 7F 00 01 12 00 00 00 00 00 00 00 00 00 00 00 01 03 00 00 00 00 00 00 00 00

  


00 00 00 00

  


02

  


07 00 00 00


06


53 48 49 45 4C 44

  


01 00 00 00


00

  


05 00 00 00


04 7C 26 7C 30

  


01 00 00 00


00

  


C2 00 00 00


4A


0C 00


19 00


FF FF


17 00


FF FF FF FF


A9 68 2F 02


EB 7D 0D 01


A1 9C 09 00


A1 9C 09 00


A1 9C 09 00


A1 9C 09 00


A1 9C 09 00


A1 9C 09 00


46 11 06 00


01


01


01


00 00 00 00 00 80 56 40


01


00


00


00 00 00 00 02


A0 86 01 00


04


00


A0 86 01 00


40 0D 03 00


40 0D 03 00


00 00 00 00


40 9C 00 00


00


00 01 01 01 01 01


01


01


01 01 00


00 00 00 00 00 00 00 00


0F 00 03 01 00 00 00 40 9C 00 00 00 26 31 35 3C 2E 76 DB 4B B6 72 35 AB DA 3A 5E BE 33 82 41 85 38 79 42 40 83 79 2B 1B 7D 37 65 33 00 00 00 00 FF FF FF 7F FF FF FF 7F 00 01 12 00 00 00 00 00 00 00 00 00 00 00 01 03 00 00 00 00 00 00 00 00

  


00 00 00 00

  


02

  


02 00 00 00


01


34

  


01 00 00 00


00

  


05 00 00 00


04 7C 26 7C 30

  


01 00 00 00


00

  


C2 00 00 00


4A


0C 00


01 00


FF FF


17 00


FF FF FF FF


E9 A1 01 02


EB 7D 0D 01


A1 9C 09 00


A1 9C 09 00


A1 9C 09 00


A1 9C 09 00


A1 9C 09 00


A1 9C 09 00


46 11 06 00


01


01


01


00 00 00 00 00 80 56 40


01


00


00


00 00 00 00 02


A0 86 01 00


04


00


A0 86 01 00


40 0D 03 00


40 0D 03 00


00 00 00 00


40 9C 00 00


00


00 01 01 01 01 01


01


01


01 01 00


00 00 00 00 00 00 00 00


0F 00 03 01 00 00 00 40 9C 00 00 00 26 31 35 3C 2E 76 DB 4B B6 72 35 AB DA 3A 5E BE 33 82 41 85 38 79 42 40 83 79 2B 1B 7D 37 65 33 00 00 00 00 FF FF FF 7F FF FF FF 7F 00 01 12 00 00 00 00 00 00 00 00 00 00 00 01 03 00 00 00 00 00 00 00 00

  


00 00 00 00

  


02

  


02 00 00 00


01


33

  


01 00 00 00


00

  


05 00 00 00


04 7C 26 7C 30

  


01 00 00 00


00

  


C2 00 00 00


4A


0C 00


19 00


FF FF


17 00


FF FF FF FF


E9 A1 01 02


2B C0 1C 01


A1 9C 09 00


A1 9C 09 00


A1 9C 09 00


A1 9C 09 00


A1 9C 09 00


A1 9C 09 00


46 11 06 00


01


01


01


00 00 00 00 00 80 56 40


01


00


00


00 00 00 00 02


A0 86 01 00


04


00


A0 86 01 00


40 0D 03 00


40 0D 03 00


00 00 00 00


40 9C 00 00


00


00 01 01 01 01 01


01


01


01 01 00


00 00 00 00 00 00 00 00


0F 00 03 01 00 00 00 40 9C 00 00 00 26 31 35 3C 2E 76 DB 4B B6 72 35 AB DA 3A 5E BE 33 82 41 85 38 79 42 40 83 79 2B 1B 7D 37 65 33 00 00 00 00 FF FF FF 7F FF FF FF 7F 00 01 12 00 00 00 00 00 00 00 00 00 00 00 01 03 00 00 00 00 00 00 00 00

  


00 00 00 00

  


02

  


02 00 00 00


01


32

  


01 00 00 00


00

  


05 00 00 00


04 7C 26 7C 30

  


01 00 00 00


00

  


C2 00 00 00


4A


0C 00


FF FF


FF FF


17 00


FF FF FF FF


E9 A1 01 02


6B 02 2C 01


A1 9C 09 00


A1 9C 09 00


A1 9C 09 00


A1 9C 09 00


A1 9C 09 00


A1 9C 09 00


46 11 06 00


01


01


01


00 00 00 00 00 80 56 40


01


00


00


00 00 00 00 00


A0 86 01 00


04


00


A0 86 01 00


40 0D 03 00


40 0D 03 00


00 00 00 00


40 9C 00 00


00


00 00 01 01 01 01


01


01


01 00 00


00 00 00 00 00 00 00 00


0F 00 03 01 00 00 00 40 9C 00 00 00 26 31 35 3C 2E 76 DB 4B B6 72 35 AB DA 3A 5E BE 33 82 41 85 38 79 42 40 83 79 2B 1B 7D 37 65 33 00 00 00 00 FF FF FF 7F FF FF FF 7F 00 01 12 00 00 00 00 00 00 00 00 00 00 00 01 03 00 00 00 00 00 00 00 00

  


00 00 00 00

  


02

  


02 00 00 00


01


31

  


01 00 00 00


00

  


05 00 00 00


04 7C 26 7C 30

  


01 00 00 00


00

  


C2 00 00 00


4A


0C 00


31 00


FF FF


17 00


FF FF FF FF


E9 A1 01 02


AB 44 3B 01


A1 9C 09 00


A1 9C 09 00


A1 9C 09 00


A1 9C 09 00


A1 9C 09 00


A1 9C 09 00


46 11 06 00


01


01


01


00 00 00 00 00 80 56 40


01


00


00


00 00 00 00 02


A0 86 01 00


04


00


A0 86 01 00


40 0D 03 00


40 0D 03 00


00 00 00 00


40 9C 00 00


00


00 01 01 01 01 01


01


01


01 01 00


00 00 00 00 00 00 00 00


0F 00 03 01 00 00 00 40 9C 00 00 00 26 31 35 3C 2E 76 DB 4B B6 72 35 AB DA 3A 5E BE 33 82 41 85 38 79 42 40 83 79 2B 1B 7D 37 65 33 00 00 00 00 FF FF FF 7F FF FF FF 7F 00 01 12 00 00 00 00 00 00 00 00 00 00 00 01 03 00 00 00 00 00 00 00 00

  


00 00 00 00

  


02

  


02 00 00 00


01


30

  


01 00 00 00


00

  


05 00 00 00


04 7C 26 7C 30

  


01 00 00 00


00

  


C2 00 00 00


4A


0C 00


19 00


FF FF


05 00


FF FF FF FF


51 77 BB 01


A4 4F A3 00


60 AE 0A 00


60 AE 0A 00


60 AE 0A 00


60 AE 0A 00


60 AE 0A 00


60 AE 0A 00


E5 01 06 00


01


01


01


00 00 00 00 00 80 66 40


01


00


00


00 00 00 00 02


A0 86 01 00


04


00


A0 86 01 00


40 0D 03 00


40 0D 03 00


00 00 00 00


40 9C 00 00


00


00 01 01 01 01 01


01


01


01 01 00


00 00 00 00 00 00 00 00


0F 00 03 01 00 00 00 40 9C 00 00 00 FD 59 30 C0 71 4B E9 44 89 E9 20 D2 B2 A8 B7 97 33 82 41 85 38 79 42 40 83 79 2B 1B 7D 37 65 33 00 00 00 00 FF FF FF 7F FF FF FF 7F 00 01 12 00 00 00 00 00 00 00 00 00 00 00 01 03 00 00 00 00 00 00 00 00

  


00 00 00 00

  


02

  


02 00 00 00


01


31

  


01 00 00 00


00

  


05 00 00 00


04 7C 26 7C 30

  


01 00 00 00


00

  


C2 00 00 00


4A


0C 00


0B 00


FF FF


05 00


FF FF FF FF


51 77 BB 01


E4 91 B2 00


60 AE 0A 00


60 AE 0A 00


60 AE 0A 00


60 AE 0A 00


60 AE 0A 00


60 AE 0A 00


E5 01 06 00


01


01


01


00 00 00 00 00 80 66 40


01


00


00


00 00 00 00 02


A0 86 01 00


04


00


A0 86 01 00


40 0D 03 00


40 0D 03 00


00 00 00 00


40 9C 00 00


00


00 01 01 01 01 01


01


01


01 01 00


00 00 00 00 00 00 00 00


0F 00 03 01 00 00 00 40 9C 00 00 00 FD 59 30 C0 71 4B E9 44 89 E9 20 D2 B2 A8 B7 97 33 82 41 85 38 79 42 40 83 79 2B 1B 7D 37 65 33 00 00 00 00 FF FF FF 7F FF FF FF 7F 00 01 12 00 00 00 00 00 00 00 00 00 00 00 01 03 00 00 00 00 00 00 00 00

  


00 00 00 00

  


02

  


02 00 00 00


01


32

  


01 00 00 00


00

  


05 00 00 00


04 7C 26 7C 30

  


01 00 00 00


00

  


C2 00 00 00


4A


0C 00


07 00


FF FF


05 00


FF FF FF FF


51 77 BB 01


24 D4 C1 00


60 AE 0A 00


60 AE 0A 00


60 AE 0A 00


60 AE 0A 00


60 AE 0A 00


60 AE 0A 00


E5 01 06 00


01


01


01


00 00 00 00 00 80 66 40


01


00


00


00 00 00 00 02


A0 86 01 00


04


00


A0 86 01 00


40 0D 03 00


40 0D 03 00


00 00 00 00


40 9C 00 00


00


00 01 01 01 01 01


01


01


01 01 00


00 00 00 00 00 00 00 00


0F 00 03 01 00 00 00 40 9C 00 00 00 FD 59 30 C0 71 4B E9 44 89 E9 20 D2 B2 A8 B7 97 33 82 41 85 38 79 42 40 83 79 2B 1B 7D 37 65 33 00 00 00 00 FF FF FF 7F FF FF FF 7F 00 01 12 00 00 00 00 00 00 00 00 00 00 00 01 03 00 00 00 00 00 00 00 00

  


00 00 00 00

  


02

  


02 00 00 00


01


33

  


01 00 00 00


00

  


05 00 00 00


04 7C 26 7C 30

  


01 00 00 00


00

  


C2 00 00 00


4A


0C 00


0C 00


FF FF


05 00


FF FF FF FF


51 77 BB 01


64 16 D1 00


60 AE 0A 00


60 AE 0A 00


60 AE 0A 00


60 AE 0A 00


60 AE 0A 00


60 AE 0A 00


E5 01 06 00


01


01


01


00 00 00 00 00 80 66 40


01


00


00


00 00 00 00 02


A0 86 01 00


04


00


A0 86 01 00


40 0D 03 00


40 0D 03 00


00 00 00 00


40 9C 00 00


00


00 01 01 01 01 01


01


01


01 01 00


00 00 00 00 00 00 00 00


0F 00 03 01 00 00 00 40 9C 00 00 00 FD 59 30 C0 71 4B E9 44 89 E9 20 D2 B2 A8 B7 97 33 82 41 85 38 79 42 40 83 79 2B 1B 7D 37 65 33 00 00 00 00 FF FF FF 7F FF FF FF 7F 00 01 12 00 00 00 00 00 00 00 00 00 00 00 01 03 00 00 00 00 00 00 00 00

  


00 00 00 00

  


02

  


02 00 00 00


01


34

  


01 00 00 00


00

  


05 00 00 00


04 7C 26 7C 30

  


01 00 00 00


00

  


C2 00 00 00


4A


0C 00


18 00


FF FF


05 00


FF FF FF FF


51 77 BB 01


A4 58 E0 00


60 AE 0A 00


60 AE 0A 00


60 AE 0A 00


60 AE 0A 00


60 AE 0A 00


60 AE 0A 00


E5 01 06 00


01


01


01


00 00 00 00 00 80 66 40


01


00


00


00 00 00 00 02


A0 86 01 00


04


00


A0 86 01 00


40 0D 03 00


40 0D 03 00


00 00 00 00


40 9C 00 00


00


00 01 01 01 01 01


01


01


01 01 00


00 00 00 00 00 00 00 00


0F 00 03 01 00 00 00 40 9C 00 00 00 FD 59 30 C0 71 4B E9 44 89 E9 20 D2 B2 A8 B7 97 33 82 41 85 38 79 42 40 83 79 2B 1B 7D 37 65 33 00 00 00 00 FF FF FF 7F FF FF FF 7F 00 01 12 00 00 00 00 00 00 00 00 00 00 00 01 03 00 00 00 00 00 00 00 00

  


00 00 00 00

  


02

  


02 00 00 00


01


35

  


01 00 00 00


00

  


05 00 00 00


04 7C 26 7C 30

  


01 00 00 00


00

  


C2 00 00 00


4A


0C 00


1E 00


FF FF


05 00


FF FF FF FF


51 77 BB 01


E4 9A EF 00


60 AE 0A 00


60 AE 0A 00


60 AE 0A 00


60 AE 0A 00


60 AE 0A 00


60 AE 0A 00


E5 01 06 00


01


01


01


00 00 00 00 00 80 66 40


01


00


00


00 00 00 00 02


A0 86 01 00


04


00


A0 86 01 00


40 0D 03 00


40 0D 03 00


00 00 00 00


40 9C 00 00


00


00 01 01 01 01 01


01


01


01 01 00


00 00 00 00 00 00 00 00


0F 00 03 01 00 00 00 40 9C 00 00 00 FD 59 30 C0 71 4B E9 44 89 E9 20 D2 B2 A8 B7 97 33 82 41 85 38 79 42 40 83 79 2B 1B 7D 37 65 33 00 00 00 00 FF FF FF 7F FF FF FF 7F 00 01 12 00 00 00 00 00 00 00 00 00 00 00 01 03 00 00 00 00 00 00 00 00

  


00 00 00 00

  


02

  


02 00 00 00


01


36

  


01 00 00 00


00

  


05 00 00 00


04 7C 26 7C 30

  


01 00 00 00


00

  


C2 00 00 00


4A


0C 00


02 00


FF FF


05 00


FF FF FF FF


51 77 BB 01


24 DD FE 00


60 AE 0A 00


60 AE 0A 00


60 AE 0A 00


60 AE 0A 00


60 AE 0A 00


60 AE 0A 00


E5 01 06 00


01


01


01


00 00 00 00 00 80 66 40


01


00


00


00 00 00 00 02


A0 86 01 00


04


00


A0 86 01 00


40 0D 03 00


40 0D 03 00


00 00 00 00


40 9C 00 00


00


00 01 01 01 01 01


01


01


01 01 00


00 00 00 00 00 00 00 00


0F 00 03 01 00 00 00 40 9C 00 00 00 FD 59 30 C0 71 4B E9 44 89 E9 20 D2 B2 A8 B7 97 33 82 41 85 38 79 42 40 83 79 2B 1B 7D 37 65 33 00 00 00 00 FF FF FF 7F FF FF FF 7F 00 01 12 00 00 00 00 00 00 00 00 00 00 00 01 03 00 00 00 00 00 00 00 00

  


00 00 00 00

  


02

  


02 00 00 00


01


37

  


01 00 00 00


00

  


05 00 00 00


04 7C 26 7C 30

  


01 00 00 00


00

  


C2 00 00 00


4A


0C 00


0D 00


FF FF


05 00


FF FF FF FF


51 77 BB 01


64 1F 0E 01


60 AE 0A 00


60 AE 0A 00


60 AE 0A 00


60 AE 0A 00


60 AE 0A 00


60 AE 0A 00


E5 01 06 00


01


01


01


00 00 00 00 00 80 66 40


01


00


00


00 00 00 00 02


A0 86 01 00


04


00


A0 86 01 00


40 0D 03 00


40 0D 03 00


00 00 00 00


40 9C 00 00


00


00 01 01 01 01 01


01


01


01 01 00


00 00 00 00 00 00 00 00


0F 00 03 01 00 00 00 40 9C 00 00 00 FD 59 30 C0 71 4B E9 44 89 E9 20 D2 B2 A8 B7 97 33 82 41 85 38 79 42 40 83 79 2B 1B 7D 37 65 33 00 00 00 00 FF FF FF 7F FF FF FF 7F 00 01 12 00 00 00 00 00 00 00 00 00 00 00 01 03 00 00 00 00 00 00 00 00

  


00 00 00 00

  


02

  


02 00 00 00


01


38

  


01 00 00 00


00

  


05 00 00 00


04 7C 26 7C 30

  


01 00 00 00


00

  


C2 00 00 00


4A


0C 00


0A 00


FF FF


05 00


FF FF FF FF


51 77 BB 01


A4 61 1D 01


60 AE 0A 00


60 AE 0A 00


60 AE 0A 00


60 AE 0A 00


60 AE 0A 00


60 AE 0A 00


E5 01 06 00


01


01


01


00 00 00 00 00 80 66 40


01


00


00


00 00 00 00 02


A0 86 01 00


04


00


A0 86 01 00


40 0D 03 00


40 0D 03 00


00 00 00 00


40 9C 00 00


00


00 01 01 01 01 01


01


01


01 01 00


00 00 00 00 00 00 00 00


0F 00 03 01 00 00 00 40 9C 00 00 00 FD 59 30 C0 71 4B E9 44 89 E9 20 D2 B2 A8 B7 97 33 82 41 85 38 79 42 40 83 79 2B 1B 7D 37 65 33 00 00 00 00 FF FF FF 7F FF FF FF 7F 00 01 12 00 00 00 00 00 00 00 00 00 00 00 01 03 00 00 00 00 00 00 00 00

  


00 00 00 00

  


02

  


02 00 00 00


01


39

  


01 00 00 00


00

  


05 00 00 00


04 7C 26 7C 30

  


01 00 00 00


00

  


C2 00 00 00


4A


0C 00


06 00


FF FF


05 00


FF FF FF FF


51 77 BB 01


E4 A3 2C 01


60 AE 0A 00


60 AE 0A 00


60 AE 0A 00


60 AE 0A 00


60 AE 0A 00


60 AE 0A 00


E5 01 06 00


01


01


01


00 00 00 00 00 80 66 40


01


00


00


00 00 00 00 02


A0 86 01 00


04


00


A0 86 01 00


40 0D 03 00


40 0D 03 00


00 00 00 00


40 9C 00 00


00


00 01 01 01 01 01


01


01


01 01 00


00 00 00 00 00 00 00 00


0F 00 03 01 00 00 00 40 9C 00 00 00 FD 59 30 C0 71 4B E9 44 89 E9 20 D2 B2 A8 B7 97 33 82 41 85 38 79 42 40 83 79 2B 1B 7D 37 65 33 00 00 00 00 FF FF FF 7F FF FF FF 7F 00 01 12 00 00 00 00 00 00 00 00 00 00 00 01 03 00 00 00 00 00 00 00 00

  


00 00 00 00

  


02

  


03 00 00 00


02


31 30

  


01 00 00 00


00

  


05 00 00 00


04 7C 26 7C 30

  


01 00 00 00


00

  


C2 00 00 00


4A


0C 00


0E 00


FF FF


05 00


FF FF FF FF


51 77 BB 01


24 E6 3B 01


60 AE 0A 00


60 AE 0A 00


60 AE 0A 00


60 AE 0A 00


60 AE 0A 00


60 AE 0A 00


E5 01 06 00


01


01


01


00 00 00 00 00 80 66 40


01


00


00


00 00 00 00 02


A0 86 01 00


04


00


A0 86 01 00


40 0D 03 00


40 0D 03 00


00 00 00 00


40 9C 00 00


00


00 01 01 01 01 01


01


01


01 01 00


00 00 00 00 00 00 00 00


0F 00 03 01 00 00 00 40 9C 00 00 00 FD 59 30 C0 71 4B E9 44 89 E9 20 D2 B2 A8 B7 97 33 82 41 85 38 79 42 40 83 79 2B 1B 7D 37 65 33 00 00 00 00 FF FF FF 7F FF FF FF 7F 00 01 12 00 00 00 00 00 00 00 00 00 00 00 01 03 00 00 00 00 00 00 00 00

  


00 00 00 00

  


02

  


03 00 00 00


02


31 31

  


01 00 00 00


00

  


05 00 00 00


04 7C 26 7C 30

  


01 00 00 00


00

  


C2 00 00 00


4A


0C 00


1C 00


FF FF


05 00


FF FF FF FF


51 77 BB 01


64 28 4B 01


60 AE 0A 00


60 AE 0A 00


60 AE 0A 00


60 AE 0A 00


60 AE 0A 00


60 AE 0A 00


E5 01 06 00


01


01


01


00 00 00 00 00 80 66 40


01


00


00


00 00 00 00 02


A0 86 01 00


04


00


A0 86 01 00


40 0D 03 00


40 0D 03 00


00 00 00 00


40 9C 00 00


00


00 01 01 01 01 01


01


01


01 01 00


00 00 00 00 00 00 00 00


0F 00 03 01 00 00 00 40 9C 00 00 00 FD 59 30 C0 71 4B E9 44 89 E9 20 D2 B2 A8 B7 97 33 82 41 85 38 79 42 40 83 79 2B 1B 7D 37 65 33 00 00 00 00 FF FF FF 7F FF FF FF 7F 00 01 12 00 00 00 00 00 00 00 00 00 00 00 01 03 00 00 00 00 00 00 00 00

  


00 00 00 00

  


02

  


03 00 00 00


02


31 32

  


01 00 00 00


00

  


05 00 00 00


04 7C 26 7C 30

  


01 00 00 00


00

  


C2 00 00 00


4A


0C 00


15 00


FF FF


05 00


FF FF FF FF


51 77 BB 01


A4 6A 5A 01


60 AE 0A 00


60 AE 0A 00


60 AE 0A 00


60 AE 0A 00


60 AE 0A 00


60 AE 0A 00


E5 01 06 00


01


01


01


00 00 00 00 00 80 66 40


01


00


00


00 00 00 00 02


A0 86 01 00


04


00


A0 86 01 00


40 0D 03 00


40 0D 03 00


00 00 00 00


40 9C 00 00


00


00 01 01 01 01 01


01


01


01 01 00


00 00 00 00 00 00 00 00


0F 00 03 01 00 00 00 40 9C 00 00 00 FD 59 30 C0 71 4B E9 44 89 E9 20 D2 B2 A8 B7 97 33 82 41 85 38 79 42 40 83 79 2B 1B 7D 37 65 33 00 00 00 00 FF FF FF 7F FF FF FF 7F 00 01 12 00 00 00 00 00 00 00 00 00 00 00 01 03 00 00 00 00 00 00 00 00

  


00 00 00 00

  


02

  


03 00 00 00


02


31 33

  


01 00 00 00


00

  


05 00 00 00


04 7C 26 7C 30

  


01 00 00 00


00

  


C2 00 00 00


4A


0C 00


16 00


FF FF


05 00


FF FF FF FF


51 77 BB 01


E4 AC 69 01


60 AE 0A 00


60 AE 0A 00


60 AE 0A 00


60 AE 0A 00


60 AE 0A 00


60 AE 0A 00


E5 01 06 00


01


01


01


00 00 00 00 00 80 66 40


01


00


00


00 00 00 00 02


A0 86 01 00


04


00


A0 86 01 00


40 0D 03 00


40 0D 03 00


00 00 00 00


40 9C 00 00


00


00 01 01 01 01 01


01


01


01 01 00


00 00 00 00 00 00 00 00


0F 00 03 01 00 00 00 40 9C 00 00 00 FD 59 30 C0 71 4B E9 44 89 E9 20 D2 B2 A8 B7 97 33 82 41 85 38 79 42 40 83 79 2B 1B 7D 37 65 33 00 00 00 00 FF FF FF 7F FF FF FF 7F 00 01 12 00 00 00 00 00 00 00 00 00 00 00 01 03 00 00 00 00 00 00 00 00

  


00 00 00 00

  


02

  


03 00 00 00


02


31 34

  


01 00 00 00


00

  


05 00 00 00


04 7C 26 7C 30

  


01 00 00 00


00

  


C2 00 00 00


4A


0C 00


32 00


FF FF


05 00


FF FF FF FF


51 77 BB 01


24 EF 78 01


60 AE 0A 00


60 AE 0A 00


60 AE 0A 00


60 AE 0A 00


60 AE 0A 00


60 AE 0A 00


E5 01 06 00


01


01


01


00 00 00 00 00 80 66 40


01


00


00


00 00 00 00 02


A0 86 01 00


04


00


A0 86 01 00


40 0D 03 00


40 0D 03 00


00 00 00 00


40 9C 00 00


00


00 01 01 01 01 01


01


01


01 01 00


00 00 00 00 00 00 00 00


0F 00 03 01 00 00 00 40 9C 00 00 00 FD 59 30 C0 71 4B E9 44 89 E9 20 D2 B2 A8 B7 97 33 82 41 85 38 79 42 40 83 79 2B 1B 7D 37 65 33 00 00 00 00 FF FF FF 7F FF FF FF 7F 00 01 12 00 00 00 00 00 00 00 00 00 00 00 01 03 00 00 00 00 00 00 00 00

  


00 00 00 00

  


02

  


03 00 00 00


02


31 35

  


01 00 00 00


00

  


05 00 00 00


04 7C 26 7C 30

  


01 00 00 00


00

  


C2 00 00 00


4A


0C 00


26 00


FF FF


05 00


FF FF FF FF


51 77 BB 01


64 31 88 01


60 AE 0A 00


60 AE 0A 00


60 AE 0A 00


60 AE 0A 00


60 AE 0A 00


60 AE 0A 00


E5 01 06 00


01


01


01


00 00 00 00 00 80 66 40


01


00


00


00 00 00 00 02


A0 86 01 00


04


00


A0 86 01 00


40 0D 03 00


40 0D 03 00


00 00 00 00


40 9C 00 00


00


00 01 01 01 01 01


01


01


01 01 00


00 00 00 00 00 00 00 00


0F 00 03 01 00 00 00 40 9C 00 00 00 FD 59 30 C0 71 4B E9 44 89 E9 20 D2 B2 A8 B7 97 33 82 41 85 38 79 42 40 83 79 2B 1B 7D 37 65 33 00 00 00 00 FF FF FF 7F FF FF FF 7F 00 01 12 00 00 00 00 00 00 00 00 00 00 00 01 03 00 00 00 00 00 00 00 00

  


00 00 00 00

  


02

  


03 00 00 00


02


31 36

  


01 00 00 00


00

  


05 00 00 00


04 7C 26 7C 30

  


01 00 00 00


00

  


C2 00 00 00


4A


0C 00


25 00


FF FF


05 00


FF FF FF FF


51 77 BB 01


A4 73 97 01


60 AE 0A 00


60 AE 0A 00


60 AE 0A 00


60 AE 0A 00


60 AE 0A 00


60 AE 0A 00


E5 01 06 00


01


01


01


00 00 00 00 00 80 66 40


01


00


00


00 00 00 00 02


A0 86 01 00


04


00


A0 86 01 00


40 0D 03 00


40 0D 03 00


00 00 00 00


40 9C 00 00


00


00 01 01 01 01 01


01


01


01 01 00


00 00 00 00 00 00 00 00


0F 00 03 01 00 00 00 40 9C 00 00 00 FD 59 30 C0 71 4B E9 44 89 E9 20 D2 B2 A8 B7 97 33 82 41 85 38 79 42 40 83 79 2B 1B 7D 37 65 33 00 00 00 00 FF FF FF 7F FF FF FF 7F 00 01 12 00 00 00 00 00 00 00 00 00 00 00 01 03 00 00 00 00 00 00 00 00

  


00 00 00 00

  


02

  


03 00 00 00


02


31 37

  


01 00 00 00


00

  


05 00 00 00


04 7C 26 7C 30

  


01 00 00 00


00

  


C2 00 00 00


4A


0C 00


24 00


FF FF


05 00


FF FF FF FF


51 77 BB 01


E4 B5 A6 01


60 AE 0A 00


60 AE 0A 00


60 AE 0A 00


60 AE 0A 00


60 AE 0A 00


60 AE 0A 00


E5 01 06 00


01


01


01


00 00 00 00 00 80 66 40


01


00


00


00 00 00 00 02


A0 86 01 00


04


00


A0 86 01 00


40 0D 03 00


40 0D 03 00


00 00 00 00


40 9C 00 00


00


00 01 01 01 01 01


01


01


01 01 00


00 00 00 00 00 00 00 00


0F 00 03 01 00 00 00 40 9C 00 00 00 FD 59 30 C0 71 4B E9 44 89 E9 20 D2 B2 A8 B7 97 33 82 41 85 38 79 42 40 83 79 2B 1B 7D 37 65 33 00 00 00 00 FF FF FF 7F FF FF FF 7F 00 01 12 00 00 00 00 00 00 00 00 00 00 00 01 03 00 00 00 00 00 00 00 00

  


00 00 00 00

  


02

  


03 00 00 00


02


31 38

  


01 00 00 00


00

  


05 00 00 00


04 7C 26 7C 30

  


01 00 00 00


00

  


C2 00 00 00


4A


0C 00


23 00


FF FF


05 00


FF FF FF FF


51 77 BB 01


24 F8 B5 01


60 AE 0A 00


60 AE 0A 00


60 AE 0A 00


60 AE 0A 00


60 AE 0A 00


60 AE 0A 00


E5 01 06 00


01


01


01


00 00 00 00 00 80 66 40


01


00


00


00 00 00 00 02


A0 86 01 00


04


00


A0 86 01 00


40 0D 03 00


40 0D 03 00


00 00 00 00


40 9C 00 00


00


00 01 01 01 01 01


01


01


01 01 00


00 00 00 00 00 00 00 00


0F 00 03 01 00 00 00 40 9C 00 00 00 FD 59 30 C0 71 4B E9 44 89 E9 20 D2 B2 A8 B7 97 33 82 41 85 38 79 42 40 83 79 2B 1B 7D 37 65 33 00 00 00 00 FF FF FF 7F FF FF FF 7F 00 01 12 00 00 00 00 00 00 00 00 00 00 00 01 03 00 00 00 00 00 00 00 00

  


00 00 00 00

  


02

  


03 00 00 00


02


31 39

  


01 00 00 00


00

  


05 00 00 00


04 7C 26 7C 30

  


01 00 00 00


00

  


C2 00 00 00


4A


0C 00


09 00


FF FF


05 00


FF FF FF FF


51 77 BB 01


64 3A C5 01


60 AE 0A 00


60 AE 0A 00


60 AE 0A 00


60 AE 0A 00


60 AE 0A 00


60 AE 0A 00


E5 01 06 00


01


01


01


00 00 00 00 00 80 66 40


01


00


00


00 00 00 00 02


A0 86 01 00


04


00


A0 86 01 00


40 0D 03 00


40 0D 03 00


00 00 00 00


40 9C 00 00


00


00 01 01 01 01 01


01


01


01 01 00


00 00 00 00 00 00 00 00


0F 00 03 01 00 00 00 40 9C 00 00 00 FD 59 30 C0 71 4B E9 44 89 E9 20 D2 B2 A8 B7 97 33 82 41 85 38 79 42 40 83 79 2B 1B 7D 37 65 33 00 00 00 00 FF FF FF 7F FF FF FF 7F 00 01 12 00 00 00 00 00 00 00 00 00 00 00 01 03 00 00 00 00 00 00 00 00

  


00 00 00 00

  


02

  


03 00 00 00


02


32 30

  


01 00 00 00


00

  


05 00 00 00


04 7C 26 7C 30

  


01 00 00 00


00

  


C2 00 00 00


4A


0C 00


05 00


FF FF


05 00


FF FF FF FF


51 77 BB 01


A4 7C D4 01


60 AE 0A 00


60 AE 0A 00


60 AE 0A 00


60 AE 0A 00


60 AE 0A 00


60 AE 0A 00


E5 01 06 00


01


01


01


00 00 00 00 00 80 66 40


01


00


00


00 00 00 00 02


A0 86 01 00


04


00


A0 86 01 00


40 0D 03 00


40 0D 03 00


00 00 00 00


40 9C 00 00


00


00 01 01 01 01 01


01


01


01 01 00


00 00 00 00 00 00 00 00


0F 00 03 01 00 00 00 40 9C 00 00 00 FD 59 30 C0 71 4B E9 44 89 E9 20 D2 B2 A8 B7 97 33 82 41 85 38 79 42 40 83 79 2B 1B 7D 37 65 33 00 00 00 00 FF FF FF 7F FF FF FF 7F 00 01 12 00 00 00 00 00 00 00 00 00 00 00 01 03 00 00 00 00 00 00 00 00

  


00 00 00 00

  


02

  


03 00 00 00


02


32 31

  


01 00 00 00


00

  


05 00 00 00


04 7C 26 7C 30

  


01 00 00 00


00

  


C2 00 00 00


4A


0C 00


1D 00


FF FF


05 00


FF FF FF FF


51 77 BB 01


E4 BE E3 01


60 AE 0A 00


60 AE 0A 00


60 AE 0A 00


60 AE 0A 00


60 AE 0A 00


60 AE 0A 00


E5 01 06 00


01


01


01


00 00 00 00 00 80 66 40


01


00


00


00 00 00 00 02


A0 86 01 00


04


00


A0 86 01 00


40 0D 03 00


40 0D 03 00


00 00 00 00


40 9C 00 00


00


00 01 01 01 01 01


01


01


01 01 00


00 00 00 00 00 00 00 00


0F 00 03 01 00 00 00 40 9C 00 00 00 FD 59 30 C0 71 4B E9 44 89 E9 20 D2 B2 A8 B7 97 33 82 41 85 38 79 42 40 83 79 2B 1B 7D 37 65 33 00 00 00 00 FF FF FF 7F FF FF FF 7F 00 01 12 00 00 00 00 00 00 00 00 00 00 00 01 03 00 00 00 00 00 00 00 00

  


00 00 00 00

  


02

  


03 00 00 00


02


32 32

  


01 00 00 00


00

  


05 00 00 00


04 7C 26 7C 30

  


01 00 00 00


00

  


C2 00 00 00


4A


0C 00


1A 00


FF FF


05 00


FF FF FF FF


51 77 BB 01


24 01 F3 01


60 AE 0A 00


60 AE 0A 00


60 AE 0A 00


60 AE 0A 00


60 AE 0A 00


60 AE 0A 00


E5 01 06 00


01


01


01


00 00 00 00 00 80 66 40


01


00


00


00 00 00 00 02


A0 86 01 00


04


00


A0 86 01 00


40 0D 03 00


40 0D 03 00


00 00 00 00


40 9C 00 00


00


00 01 01 01 01 01


01


01


01 01 00


00 00 00 00 00 00 00 00


0F 00 03 01 00 00 00 40 9C 00 00 00 FD 59 30 C0 71 4B E9 44 89 E9 20 D2 B2 A8 B7 97 33 82 41 85 38 79 42 40 83 79 2B 1B 7D 37 65 33 00 00 00 00 FF FF FF 7F FF FF FF 7F 00 01 12 00 00 00 00 00 00 00 00 00 00 00 01 03 00 00 00 00 00 00 00 00

  


00 00 00 00

  


02

  


03 00 00 00


02


32 33

  


01 00 00 00


00

  


05 00 00 00


04 7C 26 7C 30

  


01 00 00 00


00

  


C2 00 00 00


4A


0C 00


0F 00


FF FF


05 00


FF FF FF FF


51 77 BB 01


64 43 02 02


60 AE 0A 00


60 AE 0A 00


60 AE 0A 00


60 AE 0A 00


60 AE 0A 00


60 AE 0A 00


E5 01 06 00


01


01


01


00 00 00 00 00 80 66 40


01


00


00


00 00 00 00 02


A0 86 01 00


04


00


A0 86 01 00


40 0D 03 00


40 0D 03 00


00 00 00 00


40 9C 00 00


00


00 01 01 01 01 01


01


01


01 01 00


00 00 00 00 00 00 00 00


0F 00 03 01 00 00 00 40 9C 00 00 00 FD 59 30 C0 71 4B E9 44 89 E9 20 D2 B2 A8 B7 97 33 82 41 85 38 79 42 40 83 79 2B 1B 7D 37 65 33 00 00 00 00 FF FF FF 7F FF FF FF 7F 00 01 12 00 00 00 00 00 00 00 00 00 00 00 01 03 00 00 00 00 00 00 00 00

  


00 00 00 00

  


02

  


02 00 00 00


01


31

  


01 00 00 00


00

  


05 00 00 00


04 7C 26 7C 30

  


01 00 00 00


00

  


C2 00 00 00


20


0C 00


13 00


FF FF


0A 00


FF FF FF FF


C0 78 21 02


36 DE 2A 01


A2 E7 03 00


1B 68 05 00


A2 E7 03 00


1B 68 05 00


A2 E7 03 00


1B 68 05 00


00 00 00 00


02


02


02


00 00 00 00 00 80 56 40


01


00


00


00 00 00 00 00


A0 86 01 00


04


00


A0 86 01 00


40 0D 03 00


40 0D 03 00


00 00 00 00


40 9C 00 00


00


00 00 01 01 01 01


01


01


01 00 00


00 00 00 00 00 00 00 00


FF FF 00 01 00 00 00 40 9C 00 00 00 07 B4 DB 32 3D B8 3D 48 BC D8 78 51 5E FC 58 B1 33 82 41 85 38 79 42 40 83 79 2B 1B 7D 37 65 33 00 00 00 00 FF FF FF 7F FF FF FF 7F 00 01 12 00 00 00 00 00 00 00 00 00 00 00 01 03 00 00 00 00 00 00 00 00

  


00 00 00 00

  


02

  


02 00 00 00


01


32

  


01 00 00 00


00

  


05 00 00 00


04 7C 26 7C 30

  


01 00 00 00


00

  


C2 00 00 00


20


0C 00


19 00


FF FF


0A 00


FF FF FF FF


C0 78 21 02


36 29 25 01


A2 E7 03 00


1B 68 05 00


A2 E7 03 00


1B 68 05 00


A2 E7 03 00


1B 68 05 00


00 00 00 00


02


02


02


00 00 00 00 00 80 56 40


01


00


00


00 00 00 00 00


A0 86 01 00


04


00


A0 86 01 00


40 0D 03 00


40 0D 03 00


00 00 00 00


40 9C 00 00


00


00 00 01 01 01 01


01


01


01 00 00


00 00 00 00 00 00 00 00


FF FF 00 01 00 00 00 40 9C 00 00 00 07 B4 DB 32 3D B8 3D 48 BC D8 78 51 5E FC 58 B1 33 82 41 85 38 79 42 40 83 79 2B 1B 7D 37 65 33 00 00 00 00 FF FF FF 7F FF FF FF 7F 00 01 12 00 00 00 00 00 00 00 00 00 00 00 01 03 00 00 00 00 00 00 00 00

  


00 00 00 00

  


02

  


02 00 00 00


01


33

  


01 00 00 00


00

  


05 00 00 00


04 7C 26 7C 30

  


01 00 00 00


00

  


C2 00 00 00


20


0C 00


12 00


FF FF


0A 00


FF FF FF FF


C0 78 21 02


37 74 1F 01


A2 E7 03 00


1B 68 05 00


A2 E7 03 00


1B 68 05 00


A2 E7 03 00


1B 68 05 00


00 00 00 00


02


02


02


00 00 00 00 00 80 56 40


01


00


00


00 00 00 00 00


A0 86 01 00


04


00


A0 86 01 00


40 0D 03 00


40 0D 03 00


00 00 00 00


40 9C 00 00


00


00 00 01 01 01 01


01


01


01 00 00


00 00 00 00 00 00 00 00


FF FF 00 01 00 00 00 40 9C 00 00 00 07 B4 DB 32 3D B8 3D 48 BC D8 78 51 5E FC 58 B1 33 82 41 85 38 79 42 40 83 79 2B 1B 7D 37 65 33 00 00 00 00 FF FF FF 7F FF FF FF 7F 00 01 12 00 00 00 00 00 00 00 00 00 00 00 01 03 00 00 00 00 00 00 00 00

  


00 00 00 00

  


02

  


02 00 00 00


01


34

  


01 00 00 00


00

  


05 00 00 00


04 7C 26 7C 30

  


01 00 00 00


00

  


C2 00 00 00


20


0C 00


03 00


FF FF


0A 00


FF FF FF FF


CE 0D 13 02


37 74 1F 01


A2 E7 03 00


1B 68 05 00


A2 E7 03 00


1B 68 05 00


A2 E7 03 00


1B 68 05 00


00 00 00 00


02


02


02


00 00 00 00 00 80 56 40


01


00


00


00 00 00 00 00


A0 86 01 00


04


00


A0 86 01 00


40 0D 03 00


40 0D 03 00


00 00 00 00


40 9C 00 00


00


00 00 01 01 01 01


01


01


01 00 00


00 00 00 00 00 00 00 00


FF FF 00 01 00 00 00 40 9C 00 00 00 07 B4 DB 32 3D B8 3D 48 BC D8 78 51 5E FC 58 B1 33 82 41 85 38 79 42 40 83 79 2B 1B 7D 37 65 33 00 00 00 00 FF FF FF 7F FF FF FF 7F 00 01 12 00 00 00 00 00 00 00 00 00 00 00 01 03 00 00 00 00 00 00 00 00

  


00 00 00 00

  


02

  


02 00 00 00


01


35

  


01 00 00 00


00

  


05 00 00 00


04 7C 26 7C 30

  


01 00 00 00


00

  


C2 00 00 00


20


0C 00


31 00


FF FF


0A 00


FF FF FF FF


CE 0D 13 02


36 29 25 01


A2 E7 03 00


1B 68 05 00


A2 E7 03 00


1B 68 05 00


A2 E7 03 00


1B 68 05 00


00 00 00 00


02


02


02


00 00 00 00 00 80 56 40


01


00


00


00 00 00 00 00


A0 86 01 00


04


00


A0 86 01 00


40 0D 03 00


40 0D 03 00


00 00 00 00


40 9C 00 00


00


00 00 01 01 01 01


01


01


01 00 00


00 00 00 00 00 00 00 00


FF FF 00 01 00 00 00 40 9C 00 00 00 07 B4 DB 32 3D B8 3D 48 BC D8 78 51 5E FC 58 B1 33 82 41 85 38 79 42 40 83 79 2B 1B 7D 37 65 33 00 00 00 00 FF FF FF 7F FF FF FF 7F 00 01 12 00 00 00 00 00 00 00 00 00 00 00 01 03 00 00 00 00 00 00 00 00

  


00 00 00 00

  


02

  


02 00 00 00


01


36

  


01 00 00 00


00

  


05 00 00 00


04 7C 26 7C 30

  


01 00 00 00


00

  


C2 00 00 00


20


0C 00


01 00


FF FF


0A 00


FF FF FF FF


CE 0D 13 02


36 DE 2A 01


A2 E7 03 00


1B 68 05 00


A2 E7 03 00


1B 68 05 00


A2 E7 03 00


1B 68 05 00


00 00 00 00


02


02


02


00 00 00 00 00 80 56 40


01


00


00


00 00 00 00 00


A0 86 01 00


04


00


A0 86 01 00


40 0D 03 00


40 0D 03 00


00 00 00 00


40 9C 00 00


00


00 00 01 01 01 01


01


01


01 00 00


00 00 00 00 00 00 00 00


FF FF 00 01 00 00 00 40 9C 00 00 00 07 B4 DB 32 3D B8 3D 48 BC D8 78 51 5E FC 58 B1 33 82 41 85 38 79 42 40 83 79 2B 1B 7D 37 65 33 00 00 00 00 FF FF FF 7F FF FF FF 7F 00 01 12 00 00 00 00 00 00 00 00 00 00 00 01 03 00 00 00 00 00 00 00 00

  


00 00 00 00

  


02

  


02 00 00 00


01


31

  


01 00 00 00


00

  


05 00 00 00


04 7C 26 7C 30

  


01 00 00 00


00

  


C2 00 00 00


20


0C 00


02 00


FF FF


0B 00


FF FF FF FF


0B E7 13 02


85 94 03 01


0D 69 08 00


79 35 07 00


0D 69 08 00


79 35 07 00


0D 69 08 00


79 35 07 00


00 00 00 00


02


02


02


00 00 00 00 00 00 00 00


01


00


00


00 00 00 00 00


A0 86 01 00


04


00


A0 86 01 00


40 0D 03 00


40 0D 03 00


00 00 00 00


40 9C 00 00


00


00 00 01 01 01 01


01


01


01 00 00


00 00 00 00 00 00 00 00


FF FF 00 01 00 00 00 40 9C 00 00 00 B4 CC 36 A4 12 8B DD 48 97 18 E1 C6 BA A6 65 4C 33 82 41 85 38 79 42 40 83 79 2B 1B 7D 37 65 33 00 00 00 00 FF FF FF 7F FF FF FF 7F 00 01 12 00 00 00 00 00 00 00 00 00 00 00 01 03 00 00 00 00 00 00 00 00

  


00 00 00 00

  


02

  


02 00 00 00


01


32

  


01 00 00 00


00

  


05 00 00 00


04 7C 26 7C 30

  


01 00 00 00


00

  


C2 00 00 00


20


0C 00


12 00


FF FF


0B 00


FF FF FF FF


0B E7 13 02


BA 64 0E 01


0D 69 08 00


79 35 07 00


0D 69 08 00


79 35 07 00


0D 69 08 00


79 35 07 00


00 00 00 00


02


02


02


00 00 00 00 00 00 00 00


01


00


00


00 00 00 00 00


A0 86 01 00


04


00


A0 86 01 00


40 0D 03 00


40 0D 03 00


00 00 00 00


40 9C 00 00


00


00 00 01 01 01 01


01


01


01 00 00


00 00 00 00 00 00 00 00


FF FF 00 01 00 00 00 40 9C 00 00 00 B4 CC 36 A4 12 8B DD 48 97 18 E1 C6 BA A6 65 4C 33 82 41 85 38 79 42 40 83 79 2B 1B 7D 37 65 33 00 00 00 00 FF FF FF 7F FF FF FF 7F 00 01 12 00 00 00 00 00 00 00 00 00 00 00 01 03 00 00 00 00 00 00 00 00

  


00 00 00 00

  


02

  


02 00 00 00


01


32

  


01 00 00 00


00

  


05 00 00 00


04 7C 26 7C 30

  


01 00 00 00


00

  


C2 00 00 00


20


0C 00


19 00


FF FF


10 00


FF FF FF FF


D2 EA 1F 02


84 94 03 01


0D 69 08 00


79 35 07 00


0D 69 08 00


79 35 07 00


0D 69 08 00


79 35 07 00


00 00 00 00


02


02


02


00 00 00 00 00 80 66 40


01


00


00


00 00 00 00 00


A0 86 01 00


04


00


A0 86 01 00


40 0D 03 00


40 0D 03 00


00 00 00 00


40 9C 00 00


00


00 00 01 01 01 01


01


01


01 00 00


00 00 00 00 00 00 00 00


FF FF 00 01 00 00 00 40 9C 00 00 00 B4 CC 36 A4 12 8B DD 48 97 18 E1 C6 BA A6 65 4C 33 82 41 85 38 79 42 40 83 79 2B 1B 7D 37 65 33 00 00 00 00 FF FF FF 7F FF FF FF 7F 00 01 12 00 00 00 00 00 00 00 00 00 00 00 01 03 00 00 00 00 00 00 00 00

  


00 00 00 00

  


02

  


02 00 00 00


01


31

  


01 00 00 00


00

  


05 00 00 00


04 7C 26 7C 30

  


01 00 00 00


00

  


C2 00 00 00


20


0C 00


12 00


FF FF


10 00


FF FF FF FF


D2 EA 1F 02


B9 64 0E 01


0D 69 08 00


79 35 07 00


0D 69 08 00


79 35 07 00


0D 69 08 00


79 35 07 00


00 00 00 00


02


02


02


00 00 00 00 00 80 66 40


01


00


00


00 00 00 00 00


A0 86 01 00


04


00


A0 86 01 00


40 0D 03 00


40 0D 03 00


00 00 00 00


40 9C 00 00


00


00 00 01 01 01 01


01


01


01 00 00


00 00 00 00 00 00 00 00


FF FF 00 01 00 00 00 40 9C 00 00 00 B4 CC 36 A4 12 8B DD 48 97 18 E1 C6 BA A6 65 4C 33 82 41 85 38 79 42 40 83 79 2B 1B 7D 37 65 33 00 00 00 00 FF FF FF 7F FF FF FF 7F 00 01 12 00 00 00 00 00 00 00 00 00 00 00 01 03 00 00 00 00 00 00 00 00

  


00 00 00 00

  


02

  


02 00 00 00


01


32

  


01 00 00 00


00

  


05 00 00 00


04 7C 26 7C 30

  


01 00 00 00


00

  


C2 00 00 00


20


0C 00


13 00


FF FF


11 00


FF FF FF FF


97 A7 13 02


FF 96 3C 01


0D 69 08 00


79 35 07 00


0D 69 08 00


79 35 07 00


0D 69 08 00


79 35 07 00


00 00 00 00


02


02


02


00 00 00 00 00 80 66 40


01


00


00


00 00 00 00 00


A0 86 01 00


04


00


A0 86 01 00


40 0D 03 00


40 0D 03 00


00 00 00 00


40 9C 00 00


00


00 00 01 01 01 01


01


01


01 00 00


00 00 00 00 00 00 00 00


FF FF 00 01 00 00 00 40 9C 00 00 00 B4 CC 36 A4 12 8B DD 48 97 18 E1 C6 BA A6 65 4C 33 82 41 85 38 79 42 40 83 79 2B 1B 7D 37 65 33 00 00 00 00 FF FF FF 7F FF FF FF 7F 00 01 12 00 00 00 00 00 00 00 00 00 00 00 01 03 00 00 00 00 00 00 00 00

  


00 00 00 00

  


02

  


02 00 00 00


01


31

  


01 00 00 00


00

  


05 00 00 00


04 7C 26 7C 30

  


01 00 00 00


00

  


C2 00 00 00


20


0C 00


19 00


FF FF


11 00


FF FF FF FF


97 A7 13 02


34 67 47 01


0D 69 08 00


79 35 07 00


0D 69 08 00


79 35 07 00


0D 69 08 00


79 35 07 00


00 00 00 00


02


02


02


00 00 00 00 00 80 66 40


01


00


00


00 00 00 00 00


A0 86 01 00


04


00


A0 86 01 00


40 0D 03 00


40 0D 03 00


00 00 00 00


40 9C 00 00


00


00 00 01 01 01 01


01


01


01 00 00


00 00 00 00 00 00 00 00


FF FF 00 01 00 00 00 40 9C 00 00 00 B4 CC 36 A4 12 8B DD 48 97 18 E1 C6 BA A6 65 4C 33 82 41 85 38 79 42 40 83 79 2B 1B 7D 37 65 33 00 00 00 00 FF FF FF 7F FF FF FF 7F 00 01 12 00 00 00 00 00 00 00 00 00 00 00 01 03 00 00 00 00 00 00 00 00

  


00 00 00 00

  


02

  


02 00 00 00


01


31

  


01 00 00 00


00

  


05 00 00 00


04 7C 26 7C 30

  


01 00 00 00


00

  


C2 00 00 00


20


0C 00


18 00


FF FF


0C 00


FF FF FF FF


60 AB 1F 02


34 67 47 01


0D 69 08 00


79 35 07 00


0D 69 08 00


79 35 07 00


0D 69 08 00


79 35 07 00


00 00 00 00


02


02


02


00 00 00 00 00 80 66 40


01


00


00


00 00 00 00 00


A0 86 01 00


04


00


A0 86 01 00


40 0D 03 00


40 0D 03 00


00 00 00 00


40 9C 00 00


00


00 00 01 01 01 01


01


01


01 00 00


00 00 00 00 00 00 00 00


FF FF 00 01 00 00 00 40 9C 00 00 00 B4 CC 36 A4 12 8B DD 48 97 18 E1 C6 BA A6 65 4C 33 82 41 85 38 79 42 40 83 79 2B 1B 7D 37 65 33 00 00 00 00 FF FF FF 7F FF FF FF 7F 00 01 12 00 00 00 00 00 00 00 00 00 00 00 01 03 00 00 00 00 00 00 00 00

  


00 00 00 00

  


02

  


02 00 00 00


01


32

  


01 00 00 00


00

  


05 00 00 00


04 7C 26 7C 30

  


01 00 00 00


00

  


C2 00 00 00


20


0C 00


13 00


FF FF


0C 00


FF FF FF FF


60 AB 1F 02


FF 96 3C 01


0D 69 08 00


79 35 07 00


0D 69 08 00


79 35 07 00


0D 69 08 00


79 35 07 00


00 00 00 00


02


02


02


00 00 00 00 00 80 66 40


01


00


00


00 00 00 00 00


A0 86 01 00


04


00


A0 86 01 00


40 0D 03 00


40 0D 03 00


00 00 00 00


40 9C 00 00


00


00 00 01 01 01 01


01


01


01 00 00


00 00 00 00 00 00 00 00


FF FF 00 01 00 00 00 40 9C 00 00 00 B4 CC 36 A4 12 8B DD 48 97 18 E1 C6 BA A6 65 4C 33 82 41 85 38 79 42 40 83 79 2B 1B 7D 37 65 33 00 00 00 00 FF FF FF 7F FF FF FF 7F 00 01 12 00 00 00 00 00 00 00 00 00 00 00 01 03 00 00 00 00 00 00 00 00

  


00 00 00 00
```
